# Design, Synthesis, Biological Evaluation, and Molecular Modeling Studies of Novel 2‐Aminothiazole Derivatives as Potential FOXM1 Inhibitors for Triple‐Negative Breast Cancer Therapy and Structure‐Activity Relationship

**DOI:** 10.1002/ddr.70296

**Published:** 2026-05-03

**Authors:** Khaled A. N. Abusharkh, Venhar Çınar, Alper Onder, Merve Sıkık, Mustafa Guzel, Zuhal Hamurcu, Bulent Ozpolat, Mehmet Ay, Ferah Comert Onder

**Affiliations:** ^1^ Department of Chemistry, School of Graduate Studies Çanakkale Onsekiz Mart University Çanakkale Türkiye; ^2^ Department of Chemistry, Natural Products and Drug Research Laboratory, Faculty of Science Çanakkale Onsekiz Mart University Çanakkale Türkiye; ^3^ Department of Chemistry and Chemical Technology, Faculty of Science and Technology Al‐Quds University Jerusalem Palestine; ^4^ Department of Medical Biology, Faculty of Medicine Erciyes University Kayseri Türkiye; ^5^ Department of Medical System Biology, School of Graduate Studies Çanakkale Onsekiz Mart University Çanakkale Türkiye; ^6^ Research Institute for Health Sciences and Technologies (SABITA), Center of Drug Discovery and Development Istanbul Medipol University İstanbul Türkiye; ^7^ Department of Medical Pharmacology, International School of Medicine Istanbul Medipol University, Kavacik Campus, Kavacik‐Beykoz İstanbul Türkiye; ^8^ Department of Basic Pharmaceutical Sciences, School of Pharmacy İstanbul Medipol University Beykoz‐İstanbul Türkiye; ^9^ Department of Nanomedicine Houston Methodist Research Institute Houston Texas USA; ^10^ Stephenson School of Biomedical Engineering The University of Oklahoma Norman Oklahoma USA; ^11^ Department of Medical Biology, Faculty of Medicine Çanakkale Onsekiz Mart University Çanakkale Türkiye

**Keywords:** 2‐aminothiazole, FOXM1, inhibitor, MD simulations, TNBC

## Abstract

Triple Negative Breast Cancer (TNBC) is one of the most aggressive subtypes of breast cancer (BC), which is associated with a very poor prognosis. It is a broad category of tumors with a variety of biological, clinical, and morphological characteristics. FOXM1 is a pivotal transcription factor that modulates proliferation‐associated genes through complex protein‐DNA and protein‐protein interactions, making it a highly attractive target in cancer therapy. However, existing small‐molecule inhibitors often suffer from limited specificity and efficacy. In this study, we designed, synthesized, and evaluated novel series of 2‐aminothiazole derivatives (**C1‐C15)** as potential FOXM1 inhibitors. Molecular docking and molecular dynamics (MD) simulations were employed to investigate the binding interactions of these compounds with the FOXM1 DNA‐binding domain (FOXM1‐DBD). Structural analysis highlighted the importance of crucial residues, including Asn283, His287, and Arg286, in mediating inhibitory activity. Among the synthesized compounds, **C11** exhibited remarkable structural alignment and interaction patterns with FOXM1‐DBD, comparable to the reference inhibitor FDI‐6. In vitro studies using TNBC cell lines (MDA‐MB‐231, BT‐549, and BT‐20) demonstrated that compound **C11** significantly outperformed FDI‐6 in potency. Western blot analysis revealed that **C11** effectively suppressed FOXM1 transcriptional activity at concentrations of 10 µM in BT‐549 cells and 20 µM in MDA‐MB‐231 cells. These findings underscore the potential of **C11** as a potent FOXM1 inhibitor and highlight its promise for further development in TNBC therapy.

## 1 Introduction

Breast cancer remains the most frequently diagnosed malignancy in women and is the second leading cause of cancer‐related deaths. Among its subtypes, triple‐negative breast cancer (TNBC) is particularly aggressive, with higher recurrence rates and a poorer prognosis compared to other breast cancer forms (Narrandes et al. 2018; Bayraktar et al. 2018; Guo et al. 2017; Abba et al. 2010). The limited treatment options and absence of effective targeted therapies underscore the urgent need to identify new molecular targets and develop more potent treatment strategies for TNBC patients (Abba et al. 2010; Wang et al. 2002). Notably, FOXM1 is frequently overexpressed in TNBC, and its elevated expression correlates with increased tumor aggressiveness, metastatic potential, and reduced overall survival (Bayraktar et al. 2018; Engebraaten et al. 2013). FOXM1 promotes cancer cell proliferation, invasion, and metastasis by transcriptionally activating genes involved in the cell cycle, angiogenesis, and epithelial‐mesenchymal transition (EMT) (Pilarsky et al. 2004). In our previous studies (Hamurcu et al. 2019, 2017, 2016), we demonstrated for the first time that silencing FOXM1 using siRNA could effectively suppress TNBC tumor growth in mouse models, establishing FOXM1 as a promising therapeutic target. Moreover, we revealed that FOXM1 regulates the expression of eukaryotic elongation factor 2 kinase (eEF2K), thereby enhancing proliferation, invasion, and tumorigenesis in human TNBC cells. Our investigations further showed that FOXM1 plays a role in autophagy by transcriptionally modulating the expressions of Beclin‐1 and LC3. Additionally, FOXM1 influences the expression of critical genes such as Cyclin D1, MMP‐2, and VEGF, contributing to breast cancer progression (Radhakrishnan et al. 2006; Bhat et al. 2009).

**FIGURE 1:**
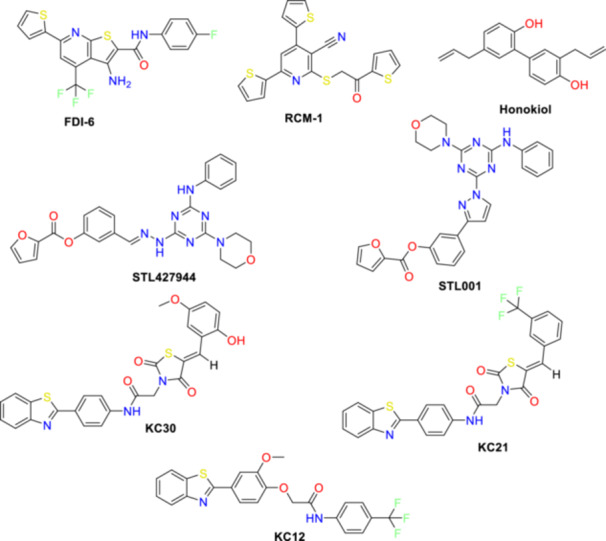
Chemical structures of some known FOXM1 inhibitors.

Despite advances in targeting FOXM1, current inhibitors have significant limitations. Proteasome inhibitors, while effective in reducing FOXM1 levels, lack specificity and raise safety concerns due to off‐target effects on multiple signaling pathways (Radhakrishnan et al. 2006; Bhat et al. 2009; Gartel 2011). Small molecules such as RCM‐1 and Honokiol suppress FOXM1 via its autoregulation loop but require high concentrations (∼50 μM) for efficacy in vitro (Halasi and Gartel 2009; Shukla et al. 2019; Tabatabaei Dakhili et al. 2020; Halasi et al. 2018). The structures of some FOXM1 inhibitors are given in Figure 1. In contrast, direct FOXM1‐DNA binding disruptors like FDI‐6 and (KC12, KC21, KC30) selectively inhibit the FOXM1 DNA‐binding domain (FOXM1‐DBD), offering mechanistic precision (Abusharkh et al. 2024a; Gormally et al. 2014; Ulhaka et al. 2021). Similarly, STL427944 and its derivative STL001 demonstrate potent suppression of FOXM1 transcriptional activity, positioning them as promising therapeutic candidates. However, the absence of a resolved FOXM1‐ligand co‐crystal structure hampers structure‐based optimization of these inhibitors, limiting their clinical development (Tabatabaei‐Dakhili et al. 2018).

**FIGURE 2:**
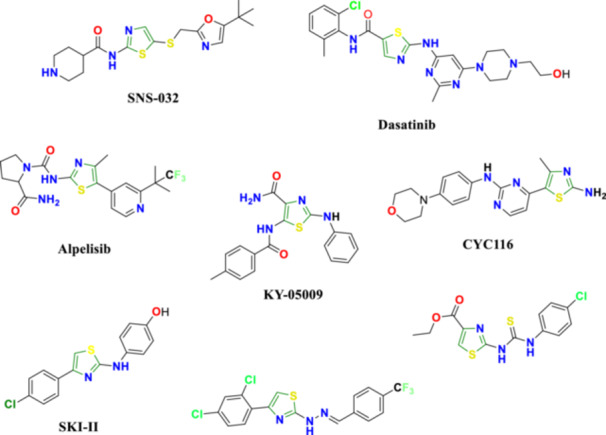
Some anticancer agents containing 2‐aminothiazole scaffold (Wan et al. 2021; Alizadeh and Hashemi 2021; Farouk Elsadek et al. 2021).

Heterocycles, particularly those containing the 2‐aminothiazole moiety, have emerged as a vital class of pharmacophores in drug discovery (Pathania et al. 2019). The 2‐aminothiazole scaffold is a key structural component in many synthetic and naturally derived compounds, contributing significantly to their diverse pharmacological activities and medicinal applications (Das et al. 2016). These derivatives exhibit a broad range of biological effects, including anti‐inflammatory (Sinha et al. 2018), anti‐diabetic (Guo et al. 2020), anti‐microbial (Amrutha et al. 2025), and antitumor properties (Ilyas et al. 2021) (Figure 2), and have shown remarkable efficacy against human cancers such as breast, lung, colon, and ovarian (Wan et al. 2021; Özbek and Gürdere 2021).

On the other hand, thiazole derivatives have garnered significant attention in cancer research due to their ability to inhibit the FOXM1 transcription factor as a critical regulator of cell proliferation and survival (Bhat et al. 2009). Recent studies indicate that thiazole‐containing compounds can effectively target the FOXM1 DNA‐binding domain, disrupting its interaction with DNA and attenuating its transcriptional activity (Abusharkh et al. 2024a; Raghuwanshi and Gartel 2023). This targeted inhibition not only suppresses tumor growth but also enhances the sensitivity of cancer cells to conventional chemotherapeutic agents (Halasi 2012). Given their capacity to modulate key molecular targets involved in cancer progression, such as kinases and transcription factors, 2‐aminothiazole derivatives are considered promising candidates for the development of novel anticancer therapeutics. Their structural versatility and potent biological activity make them particularly attractive for targeting aggressive cancers like triple‐negative breast cancer (Costa et al. 2023; Alizadeh and Hashemi 2021). Consequently, thiazole derivatives represent a promising class of molecules for developing innovative anticancer therapies aimed at mitigating FOXM1‐driven oncogenic processes.

**FIGURE 3:**
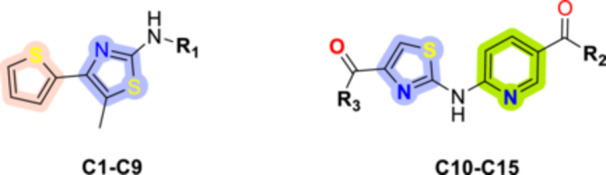
General structure of the synthesized compounds **(C1‐C15)**.

The molecular design of the target compounds (**C1‐C15**) was guided by three key structural considerations. First, the thiazole scaffold has been established as a privileged pharmacophore for FOXM1 inhibition; Bhat et al. demonstrated that thiazole‐containing antibiotics, Siomycin A and thiostrepton, selectively suppress FOXM1 transcriptional activity and expression in human cancer cells without affecting other Forkhead family members (Bhat et al. 2009). This selectivity provided a strong rationale for employing the 2‐aminothiazole core as the central scaffold in our design. Second, the 2‐aminothiazole moiety possesses both hydrogen bond donor (N‐H) and acceptor (ring N) functionalities ideally suited for interaction with the critical FOXM1‐DBD residues Asn283, Arg286, and His287. Third, structural diversification was pursued through two sub‐series: compounds **C1‐C9**, incorporating a thiophene ring to promote hydrophobic and π‐π interactions within the FOXM1‐DBD binding pocket, and compounds **C10‐C15**, featuring a pyridine–nicotinic acid linker to provide additional hydrogen bonding capacity and improved aqueous solubility. Amine‐containing side chains (morpholine, piperidine, piperazine) were systematically varied across the series to explore the role of basicity and steric bulk in modulating FOXM1 inhibitory potency. The general structure of the designed compounds is illustrated in Figure 3.

These findings collectively highlight FOXM1's central role in driving TNBC pathogenesis and validate its therapeutic relevance in this aggressive breast cancer subtype. Elucidating the molecular mechanisms underpinning FOXM1's transcriptional regulation, particularly its interaction with downstream effectors, is critical for designing precision therapies to disrupt TNBC progression. The translational potential of FOXM1 inhibition warrants further investigation, including structure‐activity optimization of lead compounds and preclinical validation in patient‐derived models. Such efforts may unlock novel combination regimens targeting FOXM1‐dependent oncogenic networks, ultimately addressing the unmet clinical needs in TNBC management.

## 2 Result and Discussion

### 2.1 Chemistry

**SCHEME 1:**
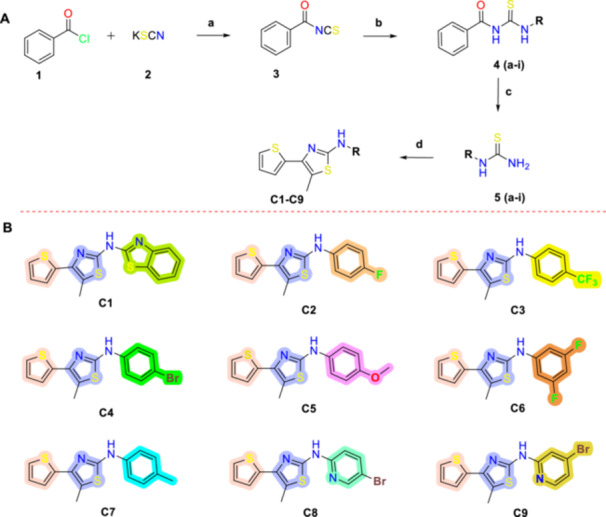
(A) Synthetic route for the preparation of the target compounds C1‐C9. Reagents and conditions: (a) Acetone, rt., 2 h, 65%; (b) aniline derivatives, DCM, rt, 6–12 h, 45%–67%; (c) MeOH, rt., 6–10 h, 65%–97%; (d) 2‐Bromo‐1‐(thiophen‐2‐yl)propan‐1‐one, EtOH, microwave (MW), 20–60 min, 51%–91%. (B) The chemical structures of the synthesized target compounds C1–C9.

**SCHEME 2:**
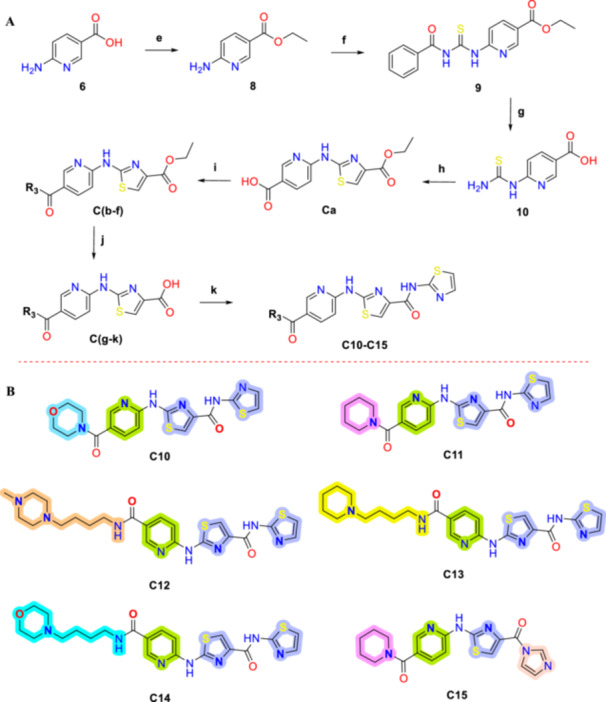
(A) Synthetic route for the preparation of the target compounds **C10‐C15**. Reagents and conditions: (e) Ethanol, thionyl chloride, 60°C, 18 h, 93%; (f) Benzoyl isothiocyanate, acetone, reflux, 5 h, 86%; (g) 1. NaOH (2 M), reflux, 1 h; 2. HCl (1 N), 68%; (h) Ethyl bromopyruvate, EtOH, under microwave irradiation, 30 min, 71%; (i) Amine derivatives, EDCI, DMAP, 0°C to rt., 24 h, 21%–75%; (j) 1. THF: EtOH (1:1); 2. KOH (10%), rt, 3 h; (k) 1. DCM, CDI, DMAP, 0°C, 1 h; 2. Thiazole‐2‐amine, rt (C15) or reflux (**C10‐C14**), 12‐24 h, 51%–90%. (B) Chemical structures of the synthesized compounds **C10‐C15**.

The synthetic approach for the preparation of compounds **C1–C15**, based on the general structure shown in Figure 2, is outlined in Schemes 1 and 2.

The synthesis of compounds **C1‐C9** was carried out in four steps, as illustrated in Scheme 1. In the first step, benzoic isothiocyanate (C(a)) was synthesized by reacting potassium isothiocyanate with benzoyl chloride in acetone at room temperature. In the second step, *N*‐Aril‐*N*‐benzoyl thiourea‐containing compounds (C1‐9(b)) were obtained by the condensation of suitable aniline derivatives with benzoic isothiocyanate (C(a)). In the third step, these *N*‐Aril‐*N*‐benzoyl thiourea containing compounds were subjected to an esterification reaction with methanol (MeOH) and potassium carbonate (K_2_CO_3_), resulting in the substitution of the benzoyl group and the formation of thiourea derivatives (C1‐9(c)). Finally, 2‐aminothiazole derivatives were synthesized in high yields through a microwave‐assisted Hantzsch thiazole synthesis, by reacting *N*‐substituted thioureas (C1‐9(c)) with 2‐bromo‐1‐(thiophen‐2‐yl)propan‐1‐one (α‐bromo ketones).

The general procedure for the synthesis of the designated compounds, **C10‐C15**, was achieved through the versatile and efficient synthetic route outlined in (Scheme 2).

The **C10–C15** compounds were synthesized through an eight‐step procedure. In the first step, the isothiocyanate intermediate **(3)** was prepared using the same method previously employed for **C1‐C9**. For further two steps, 6‐aminonicotinic acid **(6)** was transformed into ethyl 6‐aminonicotinate, designated as **(8)** via an esterification reaction catalyzed by thionyl chloride. In step three, the reaction between ethyl 6‐aminonicotinate **(8)** and benzoic isothiocyanate **(3)** produced ethyl 6‐(3‐benzoilthioureido)nicotinate **(9)**. The fourth step involved hydrolyzing ethyl 6‐(3‐benzoilthioureido)nicotinate **(9)** in the presence of sodium hydroxide (2 M), which removed the benzoyl and ethyl group and yielded 6‐thioureidonicotinic acid **(10)**.

**SCHEME 3:**
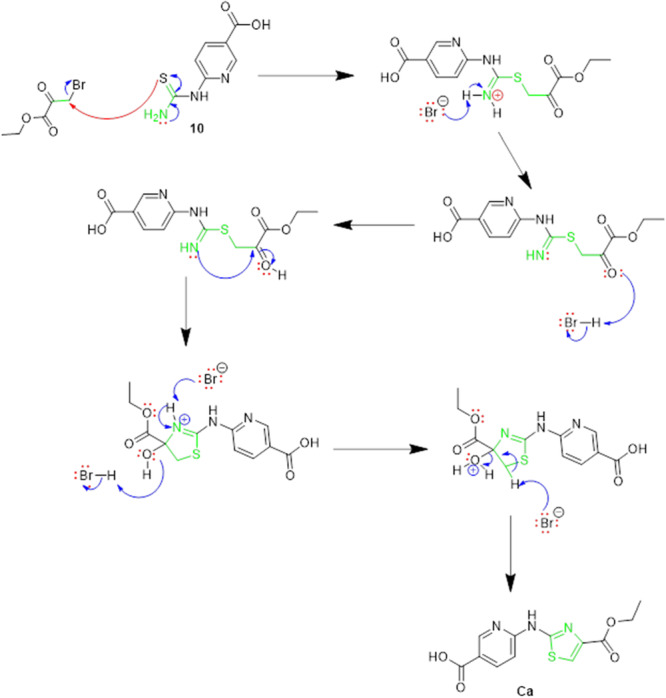
The proposed reaction mechanism of the intermediate 6‐((4‐ethoxycarbonyl)thiazole‐2‐yl)aminonicotinic acid (**Ca**).

Subsequently, a microwave‐assisted Hantzsch thiazole synthesis was employed. Here, the reaction of ethyl bromopyruvate with 6‐thioureidonicotinic acid **(10)** afforded a novel compound 6‐((4‐ethoxycarbonyl)thiazole‐2‐yl)aminonicotinic acid (**Ca**) in a high yield. The detailed reaction mechanism is illustrated in Scheme 3.

**SCHEME 4:**
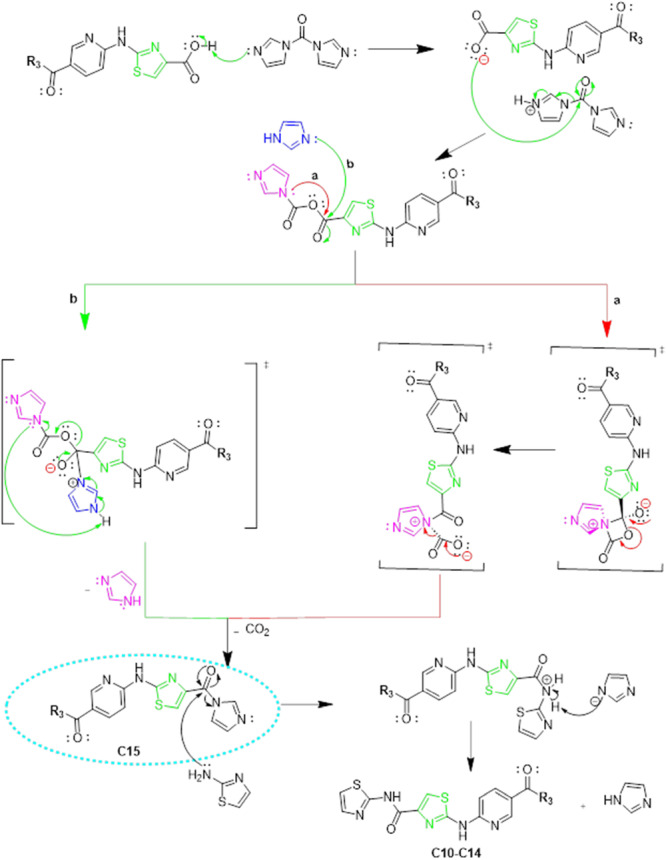
Proposed reaction mechanism for the formation of the 2‐aminothiazole derivatives (**C10–C14**) and **C15**.

This new intermediate (**Ca**) was then reacted with various aliphatic amines to form a series of ester derivatives (**Cb‐Cf**). These ester derivatives underwent basic hydrolysis to yield the corresponding carboxylic acid derivatives (**Cg‐Ck**), which were ultimately coupled through base‐catalyzed reactions to produce the final FOXM1‐targeting compounds **C10–C15**. The mechanisms illustrated in Schemes 3 and 4 are described as follows. Scheme 3 depicts the proposed mechanism for the formation of the key common intermediate **Ca** [6‐((4‐ethoxycarbonyl)thiazole‐2‐yl)aminonicotinic acid] via microwave‐assisted Hantzsch thiazole synthesis, which serves as the shared precursor for all target compounds **C10‐C15**.

In the final coupling step (Scheme 2, step **k**), the carboxylic acid intermediates (**Cg‐Ck)** were activated by CDI (DCM, DMAP, 0°C, 1 h) to generate reactive acyl imidazolide intermediates. The progress of the reaction was monitored by thin‐layer chromatography (TLC). When the reaction was initially conducted at room temperature, a new product was observed by TLC, which was subsequently isolated, purified, and characterized by ^1^H NMR, ^13^C NMR, and LC‐MS/MS analyses. Spectroscopic data revealed that the isolated product was not the intended target compound but rather a stable acyl imidazolide intermediate, designated as compound **C15**, in which the 1H‐imidazole moiety was retained. This outcome was attributed to the inherently weak nucleophilicity of Thiazole‐2‐amine as an aromatic amine, which proved insufficient to displace the imidazole leaving group at room temperature. Based on this observation, a new reaction was performed under heating to reflux conditions (18 h), which successfully afforded the intended compound **C11**. Applying the same reflux conditions to the remaining substrates yielded compounds **C10**, **C12**, **C13**, and **C14** in good yields. The proposed temperature‐dependent mechanism is illustrated in Scheme 4. Notably, this serendipitous finding opens a promising synthetic avenue for the selective preparation of novel *1H*‐imidazole‐containing analogues by conducting the reaction at room temperature, which will be explored in future studies. The synthetic route for the preparation of compounds **C10‐C15** is shown in Scheme 2.

The chemical structures of all synthesized compounds (**C1‐C15**) were unequivocally confirmed through a combination of spectroscopic techniques, including FT‐IR, ^1^H NMR, ^13^C NMR, and LC‐MS/MS analyses. The comprehensive spectroscopic data collectively substantiated the successful synthesis of the designed 2‐aminothiazole derivatives (Supporting Information S1: Figures 1–98).

The FT‐IR spectra of compounds **C1‐C9** displayed characteristic absorption bands consistent with the presence of the 2‐aminothiazole scaffold. The N‐H stretching vibrations were observed at 3189–3434 cm^−1^, indicative of secondary amine functionalities. Aromatic C‐H stretching bands appeared at 3000–3087 cm^‐1^, whereas aliphatic C‐H stretches of methyl substituents were recorded at 2810–2966 cm^‐1^. The diagnostic C = N stretching vibration of the thiazole ring appeared at 1597–1633 cm^‐1^, confirming the successful formation of the heterocyclic core. The N‐H bending vibrations were observed between 1532 and 1599 cm^‐1^, while aromatic C = C stretching bands occurred in the range of 1410–1503 cm^‐1^. Moreover, C‐N stretching vibrations (1113–1290 cm^‐1^) further supported the formation of the thiazole framework.

For compounds **C10‐C15**, additional absorption bands corresponding to carbonyl functionalities were observed. Dual C = O stretching bands were identified, with ester carbonyls resonating at higher frequencies (1698–1727 cm^‐1^) and amide carbonyls at lower frequencies (1631–1702 cm^‐1^). Multiple N‐H stretching bands (3148–3410 cm^‐1^) were indicative of both thiazole amino and amide groups. The C‐O stretching vibrations associated with ester and morpholine/piperidine moieties appeared between 1010 and 1327 cm^‐1^. In all derivatives, the thiazole C‐S stretching was consistently detected at 843–904 cm^‐1^, confirming the integrity of the heterocyclic system.

The ^1^H NMR spectra provided detailed information about the proton environments of all synthesized compounds. For **C1‐C9**, the N‐H proton appeared as a distinct singlet in the downfield region (δ 10.54–12.50 ppm in DMSO‐d_6_ or δ 7.06–7.47 ppm in CDCl_3_), suggesting involvement in hydrogen bonding. Aromatic protons of the thiophene and substituted phenyl rings resonated as multiplets between δ 6.76–8.30 ppm, showing characteristic coupling patterns for thiophene 2‐H, 3‐H, and 4‐H protons. The methyl group at position 5 of the thiazole ring consistently appeared as a sharp singlet at δ 2.45–2.56 ppm. Compounds bearing electron‐withdrawing substituents, such as CF_3_ (**C3**) and Br (**C4, C8, C9**), exhibited downfield aromatic shifts, reflecting their inductive effects. The methoxy‐substituted derivative **C5** displayed a singlet at δ 3.79 ppm, integrating for three protons.

In the spectrum of **C10‐C15**, additional resonances corresponded to aliphatic protons from morpholine, piperidine, and extended side chains. Morpholine protons in **C10** appeared as multiples at δ 3.60–3.68 ppm, whereas piperidine protons in **C11** were observed between δ 1.09–3.47 ppm. Ethyl ester protons exhibited characteristic quartet and triplet patterns (δ 4.21 and δ 1.20 ppm, *J* = 6.9 Hz). Amide N‐H protons appeared as broad singlets at δ 8.12–12.00 ppm, occasionally displaying concentration‐dependent chemical shifts due to intermolecular hydrogen bonding.

The ¹³C NMR spectra provided insight into the carbon skeletons of the target molecules. For **C1–C9**, the imine carbon (C‐2) of the thiazole ring appeared at δ 157.71–162.19 ppm, confirming its linkage to nitrogen atoms. The C‐4 and C‐5 thiazole carbons resonated at δ 115.34–116.94 ppm and δ 135.03–140.43 ppm, respectively. Aromatic carbons from the thiophene and phenyl rings were observed between δ 118.76–143.73 ppm, while the thiazole‐methyl carbon consistently appeared in the up‐field region (δ 11.13–12.49 ppm). Fluorinated derivatives (**C2, C3, C6**) exhibited characteristic ^13^C‐^19^F coupling patterns. In **C2**, the carbon directly bonded to fluorine showed a one‐bond coupling constant (^*1*^ *J*_*CF*_ = 179.5 Hz), while ortho and meta carbons displayed ^*2*^ *J*_*CF*_ = 22.9 Hz and ^*3*^ *JCF* = 5.5 Hz, respectively. For **C3**, the CF_3_ carbon exhibited a quartet with ^*1*^ *J*_*CF*_ = 270.6 Hz, confirming trifluoromethyl substitution.

For **C10‐C15**, carbonyl carbons appeared at δ 159.78–177.21 ppm, with ester carbonyls generally more deshielded than amide ones. Morpholine C‐O carbons were detected at δ 66.68 ppm (**C14**), while piperidine carbons resonated between δ 24.47–56.06 ppm. Aliphatic carbons from extended chains appeared between δ 21.35–58.43 ppm, verifying the presence of *N*‐methylpiperazine, piperidine, and morpholine substituents.

LC‐MS/MS analyses provided molecular ion confirmation for all synthesized derivatives. The spectra consistently displayed [M + H]^+^ pseudomolecular ions as base peaks, verifying the expected molecular formulas. For **C1‐C9**, the observed m/z values (286‐353) agreed with calculated molecular weights. Bromine‐containing compounds (**C4, C8, C9**) exhibited the characteristic 1:1 isotopic pattern of ^79^Br/^81^Br, with [M + H]^+^ and [M + 2]^+^ peaks (e.g., **C4**: m/z 351/353; C8: m/z 352.05/353.95), confirming monobrominated structures. The more complex derivatives **C10‐C15** displayed higher molecular ions (m/z 383–501). Compound **C11** identified as the most potent FOXM1 inhibitor exhibited a strong [M + H]^+^ peak at m/z 415.15 (calcd. for C_20_H_23_N_4_O_2_S_2_ = 415.13). Nitrogen‐rich derivatives (**C12‐C14**) containing piperazine or piperidine fragments showed characteristic fragmentation profiles consistent with retention of the thiazole‐nicotinic acid core. The combined evidence from NMR and MS analyses unambiguously confirmed the proposed molecular structures and purity of all synthesized 2‐aminothiazole derivatives, validating them for subsequent biological evaluation.

Spectroscopic trends also provided valuable insights into the electronic properties of the synthesized library. The downfield shift of N‐H signals in derivatives bearing electron‐withdrawing substituents (e.g., CF_3_, Br) indicated reduced electron density at the nitrogen center, which could influence FOXM1‐DBD binding interactions. Conversely, electron‐donating groups such as methoxy induced shielding effects, suggesting enhanced electron density. These variations in electronic distribution are likely responsible for the differences in biological activity across the compound series, with **C11** achieving a favorable balance between electronic and steric factors for potent FOXM1 inhibition. The persistence of diagnostic thiazole signals across all spectra highlights the structural robustness of the thiazole core under diverse reaction conditions, reinforcing its role as a privileged scaffold for FOXM1 inhibitor development.

### 2.2 In Vitro Activity

#### 2.2.1 Cell Viability Assay

The cytotoxic effects of all 15 synthesized compounds (**C1‐C15**) were evaluated against three TNBC cell lines (MDA‐MB‐231, BT‐549, and BT‐20) using the 3‐(4,5‐dimethylthiazol‐2‐yl)‐2,5‐diphenyltetrazolium bromide (MTT) assay over a 96h treatment period. The IC_50_ values of all compounds are summarized in Table 1.

**TABLE 1:** Cytotoxicity of the compounds **C1‐C15** in TNBC cell lines (MDA‐MB‐231, BT‐20, and BT‐549).

	Human TNBC cell lines (IC_50_, μM)		
Compounds	MDA‐MB‐231	BT‐549	BT‐20
**C1**	> 40	> 40	> 40
**C2**	> 40	34.81 ± 1.41	28.06 ± 4.91
**C3**	20.77 ± 0.79	14.19 ± 1.35	18.10 ± 5.4
**C4**	25.27 ± 1.38	> 20	17.41 ± 1.78
**C5**	> 40	> 40	38.50 ± 5.86
**C6**	26.87 ± 1.48	16.90 ± 1.27	9.21 ± 0.82
**C7**	> 40	29.45 ± 2.34	21.56 ± 2.2
**C8**	29.65 ± 6.38	27.93 ± 4.56	28.65 ± 4.63
**C9**	> 40	> 40	35.45 ± 5.7
**C10**	> 40	19.45 ± 2.31	> 40
**C11**	**2.54** ± **1.27**	**3.70** ± **2.34**	**13.31** ± **1.32**
**C12**	> 40	26.21 ± 1.23	23.35 ± 2.08
**C13**	> 40	29.24 ± 1.8	22.28 ± 1.42
**C14**	40.67 ± 6.67	34.35 ± 1.88	26.56 ± 1.67
**C15**	> 40	7.67 ± 1.03	14.41 ± 1.95
**FDI‐6**	20.79 ± 4.73^a^	13.39 ± 1.58	18.55 ± 1.4

*Note:* ± indicates standard deviation (SD).

^a^ indicates ref. Abusharkh et al. (2024a).

Among compounds **C1‐C9**, bearing the thiophene‐based 2‐aminothiazole scaffold, compounds **C3**, **C4**, **C6**, and **C8** exhibited moderate cytotoxicity with IC_50_ values ranging from 9.21 to 29.65 μM across the tested cell lines (Table 1). Notably, **C6** showed the highest potency within this sub‐series against BT‐20 cells (IC_50_ = 9.21 ± 0.82 μM). In contrast, compounds **C1**, **C5**, **C7**, and **C9** showed weak or negligible cytotoxic activity (IC_50_ > 40 μM; Table 1).

Among compounds **C10‐C15**, bearing the pyridine–nicotinic acid scaffold, compound **C11** emerged as the most potent derivative across all three cell lines, with IC_50_ values of 2.54 ± 1.27 μM (MDA‐MB‐231), 3.70 ± 2.34 μM (BT‐549), and 13.31 ± 1.32 μM (BT‐20) (Table 1). Compound **C15** also showed notable activity against BT‐549 and BT‐20 cell lines (IC_50_ = 7.67 ± 1.03 and 14.41 ± 1.95 μM, respectively), while compounds **C10**, **C12**, **C13**, and **C14** displayed moderate cytotoxicity (Table 1).

Notably, **C11** demonstrated a superior cytotoxic profile compared to the known FOXM1 inhibitor FDI‐6. While FDI‐6 inhibited MDA‐MB‐231, BT‐549, and BT‐20 cells at 20.79 ± 4.73 (Abusharkh et al. 2024a), 13.39 ± 1.58, and 18.55 ± 1.40 μM, respectively, **C11** achieved comparable or greater inhibition at substantially lower concentrations across all three tested cell lines (Table 1). These findings suggest that **C11** exerts a more potent anticancer effect, potentially through enhanced inhibition of FOXM1, a transcription factor critically implicated in cancer progression, highlighting **C11** as a promising candidate for further preclinical development.

#### 2.2.2 Western Blotting

**FIGURE 4:**
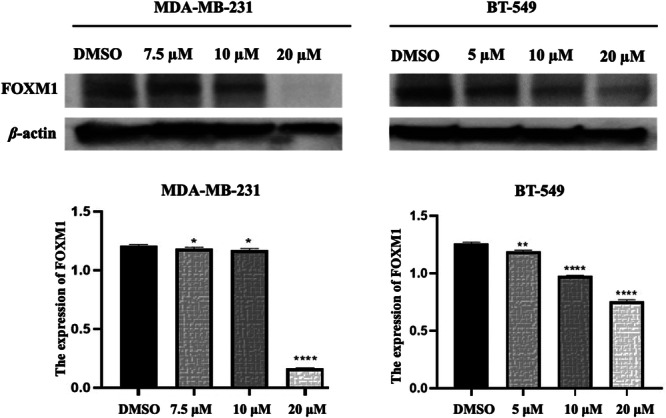
Evaluation of FOXM1 protein expression levels and the dose‐dependent inhibitory effects of compound **C11** on TNBC cell lines (MDA‐MB‐231 and BT‐549). Statistically significance was determined using one‐way ANOVA, with significance levels indicated as follows: ** = *p* ≤ 0.01, *** = *p* ≤ 0.001 **** = *p* ≤ 0.0001 compared to the DMSO control.

FOXM1 protein expression levels in MDA‐MB‐231 and BT‐549 cell lines were assessed following the treatment with compound **C11**. The FOXM1 protein expression levels were inhibited by the varying concentrations of the compound **C11** at 10 μM and 20 μM for MDA‐MB‐231 cells and 5 μM, 10 μM, and 20 μM for BT‐549 cells (Figure 4). The results demonstrated a dose‐dependent reduction in FOXM1 expression in both cell lines. These findings suggest that **C11** effectively inhibits FOXM1 expression in TNBC cells, highlighting their potential as therapeutic agents.

### 2.3 ADMET/RO5 and Toxicity Predictions

The Absorption, Distribution, Metabolism, Excretion, and Toxicity (ADMET)/Lipinski's Rule of Five (RO5) and in silico toxicity predictions for all synthesized compounds are provided in the Supporting Information (Supporting Information S1: Tables 1, 2). Based on the results in (Supporting Information S1: Table 1), the solubility levels of the compounds range from 1 to 3. Except for **C1, C3, C4**, and **C7**, the AlogP98 values fall within the acceptable range, and none of the compounds act as cytochrome P450 inhibitors. Furthermore, except for **C1, C13**, and **C14**, the remaining compounds are predicted to function as hepatotoxicity inhibitors, while compounds **C1‐C9** exhibit strong blood‐brain barrier penetration. The molecular weights (MW) of the synthesized compounds range from 286 to 499 g/mol, which is within the accepted range. Most of these compounds are predicted to be non‐carcinogenic (Supporting Information S1: Table 2).

### 2.4 Molecular Docking

AutoDock Vina was employed to investigate the binding interactions of synthesized compounds with the FOXM1 DNA‐binding domain (FOXM1‐DBD, PDB: 3G73). Computational docking analysis was performed to assess the capacity of compounds **C1‐C15** to interact with critical binding sites and exert potential anticancer effects. The calculated binding affinities spanned from ‐6.6 to ‐4.6 kcal/mol, with derivatives **C10‐C15** demonstrating superior energetic favorability relative to **C1‐C9** (Supporting Information S1: Table 3).

As a pivotal regulator of cellular proliferation, FOXM1 plays a central role in oncogenic processes (Littler et al. 2010). Its DBD contains a highly conserved structural motif that facilitates sequence‐specific recognition of promoter elements in target genes (Littler et al. 2010). Interference with FOXM1‐DNA recognition represents a validated anti‐neoplastic approach, as demonstrated in experimental models (Xiang et al. 2016). Nevertheless, substantial obstacles remain in clinical translation due to the structural properties of FOXM1. The absence of well‐formed ligand‐binding cavities and the extensive protein‐DNA contact surface of the DBD (Fontaine et al. 2015) impose constraints on inhibitor development. Literature evidence underscores the functional importance of the DBD in mediating DNA recognition. Key amino acids Asn‐283, Arg‐286, and His‐287‐are evolutionarily conserved and play indispensable roles in stabilizing protein‐DNA complexes (Halasi et al. 2018; Abusharkh et al. 2024a; Tabatabaei‐Dakhili et al. 2018; Zhang et al. 2022; Luo et al. 2021; Tabatabaei Dakhili et al. 2019). Rational design strategies focused on these residues may facilitate the development of next‐generation FOXM1‐targeted therapeutics.

**FIGURE 5:**
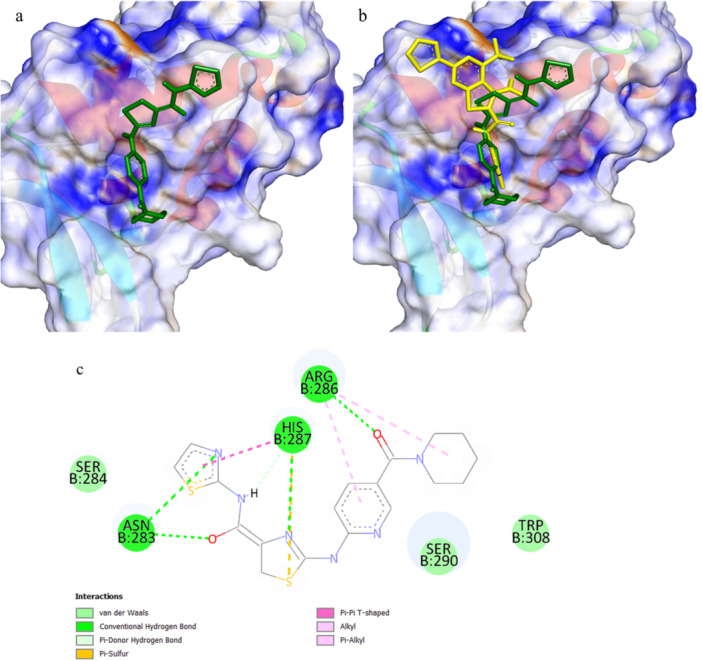
Molecular docking analysis of **C11** in the FOXM1‐DBD binding pocket (PDB ID: 3G73). (a) Three‐dimensional (3D) of the docked FOXM1‐DBD‐**C11** complex. (b) Overlay of predicted **C11** (green) and standard FDI‐6 (yellow) binding modes in FOXM1‐DBD active site. (c) Two‐dimensional (2D) illustration of **C11**‐FOXM1‐DBD interactions.

Detailed structural examination of the FOXM1‐DBD‐**C11** assembly identified Asn283, His287, and Arg286 as principal interaction sites (Figure 5). Hydrogen bonding networks were established through Asn283, Arg286, and His287, while Arg286 additionally engaged in hydrophobic pi‐alkyl and alkyl contacts. His287 contributed further stabilization via pi‐pi T‐shaped and pi‐sulfur interactions. Comparative analysis with the reference inhibitor FDI‐6 revealed that **C11** adopts an alternative binding orientation (Figure 5), which may confer enhanced inhibitory capacity against FOXM1‐DBD.

Computational binding studies indicate that the molecular architecture of **C11** enables favorable interactions with functionally important residues in FOXM1‐DBD, potentially accounting for its superior inhibitory performance. Experimental validation supports the efficacy of **C11**'s binding configuration (Figure 5) in suppressing FOXM1‐DBD activity. These structural insights may guide the rational optimization of FOXM1 inhibitors with improved therapeutic potential.

### 2.5 Molecular Dynamics (MD) Simulation Analysis of C11

**FIGURE 6:**
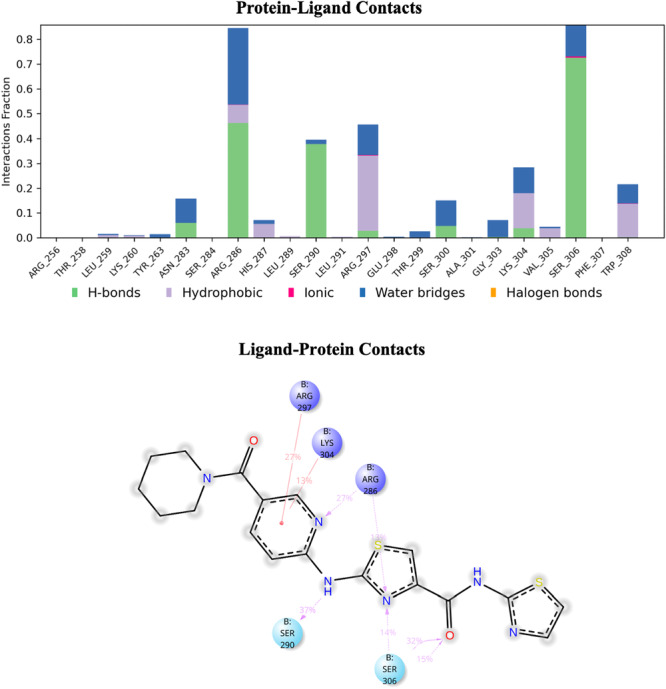
Temporal evolution of interactions between FOXM1‐DBD and compound **C11** during 100 ns MD simulation. The interactions are classified into distinct types, including charge (positive), hydrophobic, π‐π stacking, polar, ionic, π‐cation, and water bridge interactions. The “Interactions Fraction” parameter represents the proportion of simulation time during which each interaction remains stable.

Molecular dynamics (MD) simulations were conducted to investigate the stability and binding interactions of compound **C11** within the target protein's active site (Figure 6). The interaction profile, depicted in the bar graph, reveals that hydrogen bonding (H‐bonds) interaction plays a predominant role in stabilizing the ligand‐protein complex. Specifically, Arg286, Ser284, and Ser306 exhibit strong hydrogen bonding interaction. Hydrophobic interactions, primarily with residues such as Leu289, Leu291, and Trp308, contribute to ligand stabilization within the binding pocket. Additionally, ionic interactions are observed at minimal levels, while water bridge interactions further reinforce ligand binding, particularly through Ser306, Ser290, and Arg297 (Figure 6).

The 2D interaction diagram highlights critical binding interactions, including π‐π stacking with aromatic residues and π‐cation interactions, particularly involving Arg286 and Lys304. The presence of multiple hydrogen bonds and hydrophobic interactions suggests that compound **C11** forms a stable complex with the target protein, which may contribute to its anticancer activity (Figure 6).

Overall, the MD simulation results indicate that compound **C11** exhibits strong binding affinity and stability within the active site, supporting its potential as an effective anticancer agent.

## 3 Structure‐Activity Relationship

**FIGURE 7:**
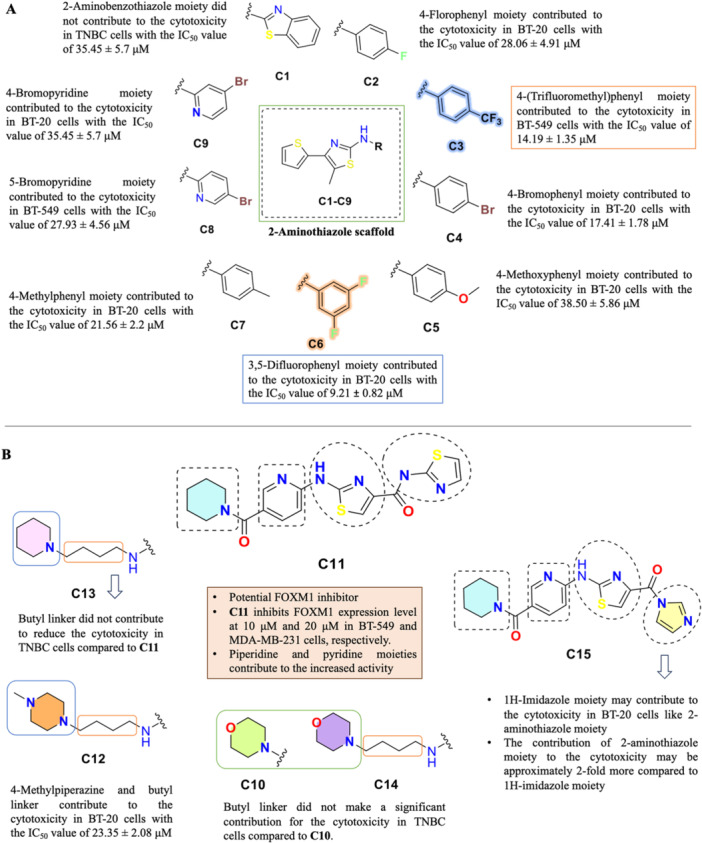
A representative illustration and the effect on the cell cytotoxicity of the synthesized **C1‐C15**. (A) Compounds C1–C9. (B) Compounds C10–C15.

A schematic illustration of the structure‐activity relationship (SAR) for the synthesized compounds **C1‐C15** is given in Figure 7. The SAR analysis on the functional groups of the synthesized compounds was based on the cytotoxic activity data (IC_50_) obtained from the MTT assay against three TNBC cell lines (MDA‐MB‐231, BT‐549, and BT‐20), as summarized in Table 1. It should be noted that FOXM1 inhibitory activity was experimentally confirmed only for compound **C11** by Western blot analysis; therefore, the following SAR discussion is based on cytotoxic potency as the primary readout.

Among the compounds **C1‐C9**, bearing a thiophene‐substituted 2‐aminothiazole scaffold, moderate cytotoxicity was observed for **C3**, **C4**, **C6**, and **C8**, while compounds **C1**, **C5**, **C7**, and **C9** were largely inactive (IC_50_ > 40 μM). These results suggest that the thiophene‐based sub‐series alone is insufficient to confer high cytotoxic potency against TNBC cell lines. Among compounds **C10‐C15**, bearing the pyridine‐nicotinic acid‐linked 2‐aminothiazole scaffold, compound **C11** (piperidine substituent) emerged as the most potent derivative. Replacement of piperidine with morpholine (**C10**), 4‐methylpiperazine with butyl chain (**C12**, **C14**), or other amine substituents resulted in a marked reduction in cytotoxic potency, highlighting the critical role of the piperidine moiety in optimizing biological activity. These findings collectively suggest that the pyridine–nicotinic acid linker combined with the piperidine substituent represents the optimal structural combination for cytotoxic potency in this series. Compound **C11** was further confirmed to suppress FOXM1 expression in a dose‐dependent manner in MDA‐MB‐231 and BT‐549 cells, establishing it as the lead FOXM1 inhibitor candidate within this library.

## 4 Conclusion

In this study, novel 2‐aminothiazole derivatives (**C1‐C15**) were synthesized and structurally characterized via spectroscopic techniques (e.g., ^1^H NMR, ^13^C NMR, and MS). The compounds were evaluated for their cytotoxic activity against TNBC cell lines (BT‐549, BT‐20, and MDA‐MB‐231), with compound **C11** emerging as the most potent candidate. **C11** significantly suppressed FOXM1 activity across all tested cell lines and demonstrated notable inhibition at 10 µM (BT‐549) and 20 µM (MDA‐MB‐231). FOXM1, a transcription factor implicated in tumor progression and chemoresistance, represents a promising therapeutic target, and the ability of 2‐aminothiazole‐containing **C11** to concurrently disrupt cancer cell proliferation and FOXM1 activity underscores its mechanistic novelty. These findings position 2‐aminothiazole derivatives, particularly **C11**, as promising anticancer candidates for further preclinical studies.

## 5 Materials and Methods

### 5.1 Chemicals

Chemicals and solvents were purchased from Merck, Apollo Scientific, and Sigma‐Aldrich and used without further purification. Melting points (°C) were determined using an X‐4 melting point apparatus. Infrared (IR) spectra were recorded on a PerkinElmer Spectrum 100 FT‐IR spectrometer. ^1^H and ^13^C nuclear magnetic resonance (NMR) spectra were acquired using JEOL ECX‐400, Varian Mercury 500 MHz, and Agilent 600 MHz NMR spectrometers. Chemical shifts (δ) are reported in parts per million (ppm), with coupling constants (*J*) given in Hertz (Hz) to indicate signal multiplicity. The NMR signals are reported with the following abbreviations: (s) singlet, (d) doublet, (t) triplet, (q) quartet, (m) multiplet, and (br) broad. Mass spectrometric analyses were performed using a Shimadzu LC‐MS/MS 8040 system. The purity of all final target compounds (**C1‐C15**) was assessed through a combination of ^1^H NMR, ^13^C NMR, and LC‐MS/MS analyses. The ^1^H NMR spectra showed no extraneous signals inconsistent with the proposed structures, and LC‐MS/MS confirmed the molecular ion [M + H]^+^ as the predominant species with no significant impurity ions detected, collectively confirming the purity of all synthesized compounds.

### 5.2 Preparation of the Target Compounds C1–C9

#### 5.2.1 Synthesis of Benzoyl Isothiocyanate (3)

A suspension of potassium isothiocyanate **(2)** (0.486 g, 5 mmol) in acetone (7 mL) was prepared, and benzoyl chloride **(1)** (540 µL, 5 mmol) was added dropwise with continuous stirring. The reaction was allowed to proceed at room temperature for 2 h. Upon completion, the precipitate was filtered, and the organic solvent was removed under reduced pressure. The resulting product, intermediate (**3)**, was obtained and used in the subsequent step without further purification (Lin et al. 2009). Yellow liquid; yield 65%; FT‐IR υ (cm^−1^): 3063 (Ar‐CH), 1962, 1925 (N = C = S), 1687 (C = O), 1597 (C = N), 1489, 1480 (Ar C = C), 1232 (N‐C), 1173 (C = N), 847 (C = S thiourea) (Lin et al. 2009) (Supporting Information S1: Figure 1).

#### 5.2.2 General Method for the Synthesis of N‐aryl‐N’‐benzoylthiourea 4(a‐i)

Benzoyl isothiocyanate **(3)** (0.82 g, 5 mmol) was dissolved in DCM (10 mL), and a solution of aniline derivatives (5 mmol) in DCM (5 mL) was added dropwise. The resulting mixture was stirred at room temperature for 6–12 h and subsequently washed with water (20 mL) and brine (20 mL). The organic phase was dried over Na_2_SO_4_, filtered, and the DCM solvent was removed under reduced pressure. The solid product was washed with diethyl ether and obtained in pure form.

##### 5.2.2.1 N‐(Benzo[*d*]thiazole‐2‐ylcarbamothioyl)benzamide (4a)

Light yellow solid; yield, 56%; m.p. 123°C–126°C; lit m.p. 97°C–98°C (Saeed and Rafique 2013); FT‐IR υ (cm^‐1^): 3324 (N‐H), 3084 (N‐H‐thiourea), 3028–2921 (Ar C‐H), 1674 (C = O), 1596 (N‐H), 1533 (C = N), 1516 (S = C‐N, thiocarbonyl), 1492–1436 (Ar C = C), 1275 (C‐N), 1241 (N‐C), 1163 (C‐N) (Supporting Information S1: Figure 2).

##### 5.2.2.2 N‐((4‐Fluorophenyl)carbamothioyl)benzamide (4b)

White solid; yield 47%; m.p. 124°C–126°C; lit. m.p. 126°C–127°C (Rauf et al. 2013). FT‐IR υ (cm^‐1^) 3364 (N‐H), 3088 (N‐H‐thiourea), 2974 (Ar C‐H), 1672 (C = O), 1610, 1568 (N‐H), 1533 (C = N), 1505 (S = C‐N, thiocarbonyl), 1489–1408 (Ar C = C), 1359 (C‐F), 1259, 1083 (C‐N), 1232 (N‐C thiourea), 1140 (C‐N) (Supporting Information S1: Figure 3).

##### 5.2.2.3 N‐((4‐(Trifluoromethyl)phenyl)carbamothioyl)benzamide (4c)

Yellow crystal; yield 65%; m.p. 133°C–135°C; lit. m.p. 138°C–139°C (Rauf et al. 2013). FT‐IR υ (cm^‐1^): 3395 (N‐H amide), 3181 (N‐H thiourea), 3030 (Ar C‐H), 1668 (C = O), 1600, 1578 (N‐H), 1520 (S = C‐N, thiocarbonyl), 1487–1410 (Ar C = C), 1316 (C‐F), 1264 (N‐C thiourea), 1148 (C‐N, carbonyl) (Supporting Information S1: Figure 4).

##### 5.2.2.4 N‐((4‐Bromophenyl)carbamothioyl)benzamide (4d)

White crystal; yield 58%; m.p. 152°C–154°C; lit. m.p. 144°C (Brito et al. 2015). FT‐IR υ (cm^‐1^): 3221 (N‐H amide str.), 3101 (N‐H thiourea), 3019 (Ar CH), 1658 (C = O), 1587, 1548 (N‐H), 1516 (S = C‐N, thiocarbonyl), 1488–1400 (Ar C = C), 1260 (N‐C thiourea), 1151 (C‐N) (Supporting Information S1: Figure 5).

##### 5.2.2.5 N‐((4‐Methoxyphenyl)carbamothioyl)benzamide (4e)

Gray crystalline solid; yield 57%; m.p. 154°C–155°C; lit. m.p. 145°C (Brito et al. 2015). FT‐IR υ (cm^‐1^): 3226 (N‐H), 3119 (N‐H thiourea), 3032–2966 (Ar C‐H), 2936–2843 (aliphatic C‐H, OCH₃), 1668 (C = O), 1596, 1532 (N‐H), 1509 (S = C‐N, thiocarbonyl), 1488–1441 (Ar C = C), 1338 (aliphatic C‐H, ‐CH_3_), 1247 (N‐C thiourea), 1152 (C‐N) (Supporting Information S1: Figure 6).

##### 5.2.2.6 N‐((3,5‐Difluorophenyl)carbamothioyl)benzamide (4f)

White crystalline solid; yield 66%; m.p. 148°C–149°C. FT‐IR υ (cm^‐1^): 3238 (N‐H amide), 3109 (N‐H thiourea), 3079–2938 (Ar C‐H), 1671 (C = O), 1611, 1571 (N‐H), 1527 (S = C‐N, thiocarbonyl), 1477–1437 (Ar C = C), 1321 (C‐F), 1261 (N‐C thiourea), 1170 (C‐N) (Supporting Information S1: Figure 7).

##### 5.2.2.7 N‐(p‐Tolylcarbamothioyl)benzamide (4g)

White crystalline solid; yield 58%; m.p. 162°C–164°C; lit. m.p. 160°C–162°C (Rauf et al. 2013). FT‐IR υ (cm^‐1^): 3240 (N‐H amide), 3121 (N‐H thiourea), 3038–3000 (Ar C‐H), 2926 (aliphatic C‐H, ‐CH_3_), 1669 (C = O), 1598, 1553 (N‐H), 1508 (S = C‐N, thiocarbonyl), 1488–1447 (Ar C = C), 1354 (aliphatic C‐H, ‐CH_3_), 1256 (N‐C thiourea), 1144 (C‐N, carbonyl) (Supporting Information S1: Figure 8).

##### 5.2.2.8 N‐((5‐Bromopyridin‐2‐yl)carbamothioyl)benzamide (4h)

White solid; yield 73%; m.p. 166°C–168°C; lit. m.p. 164°C (Jouve and Bergman 2003). FT‐IR υ (cm^‐1^): 3453 (N‐H), 3287 (N‐H thiourea), 3050–3006 (Ar C‐H), 1674 (C = O), 1623 (C = N), 1579, 1532 (N‐H), 1512 (S = C‐N, thiocarbonyl), 1487–1454 (Ar C = C), 1244 (N‐C thiourea), 1142 (C‐N, carbonyl) (Supporting Information S1: Figure 9).

##### 5.2.2.9 N‐((4‐Bromopyridin‐2‐yl)carbamothioyl)benzamide (4i)

Light pink crystalline solid; yield 45%; m.p. 158°C–160°C. FT‐IR υ (cm^‐1^): 3334 (N‐H), 3095 (N‐H thiourea), 3060–2957 (Ar C‐H), 1670 (C = O), 1599 (C = N), 1576, 1557 (N‐H), 1509 (S = C‐N, thiocarbonyl), 1488–1446 (Ar C = C), 1260 (N‐C thiourea), 1146 (C‐N) (Supporting Information S1: Figure 10).

#### 5.2.3 General Method for the Synthesis of Thiourea Derivatives 5 (a‐i)

*N*‐Aryl‐*N’*‐benzoylthiourea 4(a‐i) (2 mmol) was dissolved in methanol (5 mL), and potassium carbonate (K_2_CO_3_) (0.553 g, 4 mmol) was added. The mixture was stirred at room temperature for 6–10 h. After the evaporation of the organic solvent under reduced pressure, the resulting solid was washed with water and dried. The product was then purified by washing with toluene and diethyl ether (Et_2_O).

##### 5.2.3.1 1‐(Benzo[d]thiazole‐2‐yl)thiourea (5a)

Light yellow solid; yield 74%; m.p. 208°C–210°C; lit. m.p. 215°C–220°C (Eshkil et al. 2017). FT‐IR υ (cm^‐1^): 3243, 3183 (N‐H), 3123 (N‐H), 3021 (Ar C‐H), 1614, 1567, 1556 (N‐H), 1604 (C = N), 1456–1432 (Ar C = C), 1313, 1185 (C‐N) (Supporting Information S1: Figure 11).

##### 5.2.3.2 1‐(Benzo[d]thiazole‐2‐yl)thiourea (5b)

Cream solid; yield 65%; m.p. 167°C–168°C; lit. m.p. 146°C–148°C (Diwakar et al. 2016). FT‐IR υ (cm^‐1^): 3437, 3271 (N‐H), 3174 (N‐H), 3065, 2990 (Ar C‐H), 1622, 1605, 1541 (N‐H), 1475, 1410 (Ar C = C), 1312 (C‐F), 1262, 1152 (C‐N) (Supporting Information S1: Figure 12).

##### 5.2.3.3 1‐(4‐(Trifluoromethyl)phenyl)thiourea (5c)

Cream solid; yield 68%; m.p. 111°C–113°C; lit. m.p. 119°C–122°C (Ahdenov et al. 2022). FT‐IR υ (cm^‐1^): 3325, 3243 (N‐H), 3139 (N‐H), 3060 (Ar C‐H), 1623, 1604, 1555 (N‐H), 1410 (Ar C = C), 1316 (C‐F), 1332, 1164 (C‐N) (Supporting Information S1: Figure 13).

##### 5.2.3.4 1‐(4‐Bromophenyl)thiourea (5d)

Cream solid; yield 87%; m.p. 190°C–191°C; lit. m.p. 186°C (Ahdenov et al. 2022). FT‐IR υ (cm^‐1^): 3402, 3255 (N‐H), 3185 (N‐H), 3085–2968 (Ar C‐H), 1616, 1576 (N‐H), 1490–1449 (Ar C = C), 1296, 1009 (C‐N) (Supporting Information S1: Figure 14).

##### 5.2.3.5 1‐(4‐Bromophenyl)thiourea (5e)

Light gray solid; yield 96%; m.p. 215°C–216°C; lit. m.p. 207°C (Ahdenov et al. 2022), 198°C–200°C (Goodyer 2003). FT‐IR υ (cm^‐1^): 3400, 3274 (N‐H), 3148 (N‐H), 3011‐2982 (Ar C‐H), 2840 (aliphatic C‐H, OCH_3_), 1623, 1589, 1537 (N‐H), 1467–1443 (Ar C = C), 1299 (aliphatic C‐H, ‐CH_3_), 1241, 1018 (C‐N) (Supporting Information S1: Figure 15).

##### 5.2.3.6 1‐(3,5‐Difluorophenyl)thiourea (5f)

Cream solid; yield 96%; m.p. 107°C–109°C. FT‐IR υ (cm^‐1^): 3354, 3298 (N‐H), 3173 (N‐H), 3075–2952 (Ar C‐H), 1634, 1603, 1537 (N‐H), 1471–1428 (Ar C = C), 1281 (C‐F), 1119, 1070 (C‐N) (Supporting Information S1: Figure 16).

##### 5.2.3.7 1‐(p‐Tolyl)thiourea (5g)

White solid; yield 97%; m.p. 187°C–188°C; lit. m.p. 179°C (Ahdenov et al. 2022), 160°C–162°C (Diwakar et al. 2016). FT‐IR υ (cm^‐1^): 3435, 3267 (N‐H), 3157 (N‐H), 3078–3001 (Ar C‐H), 2945 (aliphatic C‐H), 1611, 1583, 1532 (N‐H), 1464–1408 (Ar C = C), 1387 (aliphatic C‐H, ‐CH_3_), 1310, 1236 (C‐N) (Supporting Information S1: Figure 17).

##### 5.2.3.8 1‐(5‐Bromopyridin‐2‐yl)thiourea (5h)

Cream solid; yield 92%; m.p. 233°C–235°C. FT‐IR υ (cm^‐1^): 3316, 3264 (N‐H), 3217 (N‐H), 3154, 3090 (Ar C‐H), 1611 (C = N), 1582 (N‐H), 1474–1427 (Ar C = C), 1319, 1053 (C‐N) (Supporting Information S1: Figure 18).

##### 5.2.3.9 1‐(4‐Bromopyridin‐2‐yl)thiourea (5i)

Cream solid; yield 97%; m.p. 273°C–275°C. FT‐IR υ (cm^‐1^): 3271, 3210 (N‐H, NH_2_), 3244 (N‐H), 3051, 3010 (Ar C‐H), 1608 (C = N), 1589, 1533 (N‐H), 1467‐1434 (Ar C = C), 1327, 1076 (C‐N) (Supporting Information S1: Figure 19).

#### 5.2.4 General Procedure for the Synthesis of Target Compounds (C1‐C9)

2‐Aminothiazole derivatives **(C1‐C9)** were synthesized via the Hantzsch thiazole reaction. 2‐Bromo‐1‐(thiophen‐2‐yl)propan‐1‐one (0.11 g, 0.5 mmol), thiourea derivatives 5(a‐i) (0.5 mmol), and diisopropylethylamine (DIPEA) (65 µL, 0.55 mmol) were dissolved in anhydrous ethanol (EtOH) and stirred under microwave irradiation at 80°C for 20–60 min. The resulting precipitate was collected and washed with water and cold ethanol. The product was obtained in pure form. The progress of the reaction and the purity of the compound were monitored by thin‐layer chromatography (TLC) using a mobile phase of toluene:EtOAc:formic acid (TEF) (5:4:1).

##### 5.2.4.1 N‐(5‐Methyl‐4‐(thiophen‐2‐yl)thiazole‐2‐yl)benzo[d]thiazole‐2‐amine (C1)

Light yellow solid; yield 67%; m.p. 252°C–253°C. FT‐IR υ (cm^‐1^): 3360 (N–H str.), 3072–2962 (Ar CH str.), 2914, 2850 (aliphatic C‐H, –CH_3_), 1603 (C = N), 1548 (N–H), 1481–1458 (Ar C = C), 1364 (aliphatic C–H, ‐CH_3_), 1225 (C–N) (Supporting Information S1: Figure 20). ^1^H NMR (500 MHz, DMSO*‐d6*): δ 12.50 (s, 1H), 7.93–7.86 (m, 1H), 7.56 (d, *J* = 5.2 Hz, 1H), 7.45–7.36 (m, 3H), 7.23 (t, *J* = 7.5 Hz, 1H), 7.17 (t, *J* = 4.4 Hz, 1H), 2.54 (s, 3H) (Supporting Information S1: Figure 21). ^13^C NMR (125 MHz, DMSO‐*d6*): 138.81, 128.41, 126.81, 125.91, 125.14, 123.21, 122.29, 119.49, 12.49 (Supporting Information S1: Figure 22). LC‐MS/MS (m/z): 330.00 ([M + 1]^+^; 40%) (Supporting Information S1: Figure 23).

##### 5.2.4.2 N‐(4‐Fluorophenyl)‐5‐methyl‐4‐(thiophen‐2‐yl)thiazole‐2‐amine (C2)

Light yellow solid; yield, 90%; m.p. 154°C–155°C. FT‐IR υ (cm^‐1^): 3243 (N–H), 3071–2958 (Ar C‐H), 2920, 2855 (aliphatic C‐H, –CH_3_), 1616 (C = N), 1597 (N–H), 1502 (S–C–N), 1432–1410 (Ar C = C str.), 1311 (aliphatic C–H, –CH_3_), 1233 (C–N), 1212 (C–F) (Supporting Information S1: Figure 24). ^1^H NMR (400 MHz, CDCl_3_): δ 7.29 (d, *J* = 4.3 Hz, 1H), 7.28–7.25 (m, 3H), 7.06 (t, *J* = 4.5 Hz, 2H), 7.03–6.95 (m, 2H), 2.47 (s, 3H) (Supporting Information S1: Figure 25). ^13^C NMR (125 MHz, CDCl_3_): δ 160.49 (d, ^1^ *J*_*CF*_ = 179.5 Hz), 157.85, 140.43, 138.41, 136.60 (d, ^4^ *J*_*CF*_ = 2.7 Hz), 127.41, 124.79 (d, ^3^ *J*_*CF*_ = 5.5 Hz), 120.49, 120.43, 116.07 (d, ^2^ *J*_*CF*_ = 22.9 Hz), 115.66, 12.37 (Supporting Information S1: Figure 26). LC‐MS/MS (m/z): 291.05 ([M + 1]^+^; 100.00%) (Supporting Information S1: Figure 27).

##### 5.2.4.3 5‐Methyl‐4‐(thiophen‐2‐yl)‐N‐(4‐(trifluoromethyl)phenyl)thiazole‐2‐amine (C3)

Yellow solid; yield, 74%; m.p. 130°C–131.5°C. FT‐IR υ (cm^‐1^): 3383 (N–H str.), 3052–2982 (Ar C‐H), 2938 (aliphatic C‐H, –CH_3_), 1633 (C = N), 1599 (N–H), 1455–1414 (Ar C = C), 1361 (aliphatic C–H, –CH_3_), 1321 (C–F), 1115, 1104 (C–N) (Supporting Information S1: Figure 28). ^1^H NMR (400 MHz, DMSO‐*d6*): δ 10.54 (s, 1H), 7.81 (d, *J* = 8.6 Hz, 1H), 7.63 (d, *J* = 8.5 Hz, 1H), 7.50 (d, *J* = 5.1 Hz, 1H), 7.34 (d, *J* = 3.6 Hz, 1H), 7.14–7.10 (m, 1H), 2.45 (s, 3H) (Supporting Information S1: Figure 29). ^13^C NMR (125 MHz, DMSO‐*d6*): 157.71, 143.73, 139.13, 137.75, 127.30, 125.71 (q, ^3^ *J*_*CF*_ = 3.9 Hz), 124.72, 124.05 (q, ^1^ *J*_*CF*_ = 270.6 Hz),123.98, 122.98, 120.21 (q, ^2^ *J*_*CF*_ = 31.7 Hz), 115.74, 115.63, 11.20 (Supporting Information S1: Figure 30). LC‐MS/MS (m/z): 341 ([M + 1]^+^; 100.00%) (Supporting Information S1: Figure 31).

##### 5.2.4.4 N‐(4‐Bromophenyl)‐5‐methyl‐4‐(thiophen‐2‐yl)thiazole‐2‐amine (C4)

White solid; yield, 91%; m.p. 153°C–155°C. FT‐IR υ (cm^‐1^): 3191 (N–H str.), 3087–2996 (Ar C‐H), 2937 (aliphatic CH, –CH_3_), 1615 (C = N), 1574 (N–H), 1488–1412 (Ar C = C), 1364 (aliphatic C–H, –CH_3_), 1239, 1172 (C–N) (Supporting Information S1: Figure 32). ^1^H NMR (400 MHz, CDCl_3_): δ 7.47 (d, *J* = 8.4 Hz, 3H), 7.37 (d, *J* = 5.0 Hz, 1H), 7.25 (d, *J* = 2.7 Hz, 2H), 7.11 (t, *J* = 4.2 Hz, 1H), 2.47 (s, 3H) (Supporting Information S1: Figure 33). ^13^C NMR (125 MHz, CDCl_3_): 162.19, 137.99, 135.03, 133.74, 132.51, 127.76, 126.56, 126.36, 120.78, 116.94, 113.93, 12.30 (Supporting Information S1: Figure 34). LC‐MS/MS (m/z): 351 and 353 ([M + 1]^+^, [M + 2]^+^; 100.00%, 95.18% (Supporting Information S1: Figure 35).

##### 5.2.4.5 N‐(4‐Methoxyphenyl)‐5‐methyl‐4‐(thiophen‐2‐yl)thiazole‐2‐amine (C5)

Brown solid; yield, 86%; m.p. 145°C–147°C. FT‐IR υ (cm^‐1^): 3232 (N–H), 3078–2993 (Ar C‐H), 2940–2835 (aliphatic CH, –CH_3_), 1626 (C = N), 1568 (N–H), 1444–1410 (Ar C = C), 1369, 1298 (aliphatic C–H, –CH_3_), 1240, 1180 (C–N) (Supporting Information S1: Figure 36). ^1^H NMR (400 MHz, CDCl_3_): δ 7.28–7.23 (m, 4H), 7.22 (d, *J* = 3.1 Hz, 1H), 7.05 (t, *J* = 5.3 Hz, 1H), 6.86 (d, *J* = 9.2 Hz, 2H), 3.79 (s, 3H), 2.45 (s, 3H) (Supporting Information S1: Figure 37). ^13^C NMR (125 MHz, CDCl_3_): 160.21, 154.50, 139.93, 139.26, 135.16, 128.28, 125.45, 124.61, 118.99, 114.69, 114.60. 55.68, 12.29 (Supporting Information S1: Figure 38). LC‐MS/MS (m/z): 303.05 ([M + 1]^+^; 82.36%) (Supporting Information S1: Figure 39).

##### 5.2.4.6 N‐(3,5‐Difluorophenyl)‐5‐methyl‐4‐(thiophen‐2‐yl)thiazole‐2‐amine (C6)

Cream solid; yield, 58%; m.p. 124°C–125°C. FT‐IR υ (cm^‐1^): 3189 (N–H), 3067–3023 (Ar C‐H), 2965 (aliphatic C‐H, –CH_3_), 1625 (C = N), 1604 (N–H), 1504–1411 (Ar C = C), 1363 (aliphatic C–H, –CH_3_), 1229 (C–F), 1290, 1113 (C–N) (Supporting Information S1: Figure 40). ^1^H NMR (400 MHz, CDCl_3_): δ 10.58 (s, 1H), 7.54 (d, *J* = 5.1 Hz, 1H), 7.49–7.25 (m. 3H), 7.15 (t, *J* = 4.2 Hz, 1H), 6.76 (t, *J* = 10.5 Hz, 1H), 5.51 (s, 3H) (Supporting Information S1: Figure 41). ^13^C NMR (125 MHz, CDCl_3_): 163.26 (d, ^1^ *J*_*CF*_ = 242.4 Hz), 163.26 (d, ^1^ *J*_*CF*_ = 242.9 Hz), 158.71, 143.73 (t, ^3^ *J*_*CF*_ = 14.2 Hz), 140.10, 138.85, 128.46, 125.83, 124.84, 116.66, 100.00, 96.38 (t, ^2^ *J*_*CF*_ = 26.5 Hz), 12.22 (Supporting Information S1: Figure 42). LC‐MS/MS (m/z): 309.05 ([M + 1]^+^; 100.00%) (Supporting Information S1: Figure 43).

##### 5.2.4.7 5‐Methyl‐4‐(thiophen‐2‐yl)‐N‐(p‐tolyl)thiazole‐2‐amine (C7)

White solid; yield, 91%; m.p. 153°C–155°C. FT‐IR υ (cm^‐1^): 3434 (N–H), 3069–2979 (Ar C‐H), 2966, 2938 (aliphatic CH, –CH_3_), 1611 (C = N str.), 1599 (N–H), 1503–1434 (Ar C = C), 1390, 1306 (aliphatic C–H, –CH_3_), 1290, 1170 (C–N str.) (Supporting Information S1: Figure 44). ^1^H NMR (400 MHz, CDCl_3_): δ 7.29 – 7.26 (m, 2H), 7.19 (d, *J* = 8.4 Hz, 2H), 7.13 (d, *J* = 8.4 Hz, 2H), 7.07 (t, *J* = 4.9 Hz, 1H), 2.48 (s, 3H), 2.32 (s, 3H) (Supporting Information S1: Figure 45). ^13^C NMR (125 MHz, CDCl_3_): 161.31, 140.07, 138.38, 137.82, 132.94, 129.99, 127.42, 124.75, 124.70, 118.76, 115.34, 20.79, 12.41 (Supporting Information S1: Figure 46). LC‐MS/MS (m/z): 287 ([M + 1]^+^; 100.00%) (Supporting Information S1: Figure 47).

##### 5.2.4.8 N‐(5‐Bromopyridin‐2‐yl)‐5‐methyl‐4‐(thiophen‐2‐yl)thiazole‐2‐amine (C8)

White solid; yield, 51%; m.p. 207‐208C. FT‐IR υ (cm^‐1^): 3222 (N–H), 3151–3001 (Ar CH str.), 2916, 2810 (aliphatic C‐H, –CH_3_), 1597, 1589 (C = N), 1535 (N–H), 1474–1430 (Ar C = C), 1361 (aliphatic C–H, –CH_3_), 1289, 1169 (C–N) (Supporting Information S1: Figure 48). ^1^H NMR (400 MHz, CDCl_3_): δ 10.76 (s, 1H), 8.30 (d, *J* = 2.4 Hz, 1H), 7.40–7.29 (m, 2H), 7.28–7.20 (m, 1H), 7.05 (t, *J* = 4.7 Hz, 1H), 6.15 (d, *J* = 9.2 Hz, 1H), 2.56 (s, 3H) (Supporting Information S1: Figure 49). ^13^C NMR (125 MHz, CDCl_3_): 154.53, 149.98, 146.12, 139.78, 138.31, 137.83, 127.29, 124.40, 123.57, 117.24, 112.08, 109.36, 11.15 (Supporting Information S1: Figure 50). LC‐MS/MS (m/z): 352.05, 353.95 ([M + 1]^+^, [M + 2]^+^; 62.71%, 74.90%) (Supporting Information S1: Figure 51).

##### 5.2.4.9 N‐(4‐Bromopyridin‐2‐yl)‐5‐methyl‐4‐(thiophen‐2‐yl)thiazole‐2‐amine (C9)

White solid; yield, 62%; m.p. 215°C–217°C. FT‐IR υ (cm^‐1^): 3233 (N–H), 3155–3022 (Ar C‐H), 2940, 2810 (aliphatic CH, –CH_3_), 1604, 1575 (C = N), 1534 (N–H), 1472–1431 (Ar C = C), 1366 (aliphatic C–H, –CH_3_), 1299, 1162 (C–N) (Supporting Information S1: Figure 52). ^1^H NMR (500 MHz, DMSO‐*d6*): δ 11.47 (s, 1H), 8.18 (d, *J* = 5.6 Hz, 1H), 7.51 (dd, *J* = 5.0, 1.2 Hz, 1H), 7.33 (dd, *J* = 3.7, 1.2 Hz, 1H), 7.27 (d, *J* = 1.7 Hz, 1H), 7.16–7.12 (m, 2H), 2,50 (s, 3H) (Supporting Information S1: Figure 53). ^13^C NMR (125 MHz, DMSO‐*d6*): 154.45, 152.02, 147.34, 138.29, 137.81, 131.90, 127.31, 124.41, 123.56, 118.17, 117.48, 112.29, 11.13 (Supporting Information S1: Figure 54). LC‐MS/MS (m/z): 352.00, 353.95 ([M + 1]^+^, [M + 2]^+^; 76.78%, 89.86%) (Supporting Information S1: Figure 55).

### 5.3 Preparation Procedure of the Target Compounds C10–C15

#### 5.3.1 Synthesis of ethyl 6‐aminonicotinate (8)

Under an inert atmosphere with continuous stirring, 6‑aminonicotinic acid **(7)** (1.0 mg, 7.23 mmol) was dissolved in 20 mL of ethanol (EtOH) at 60°C, and thionyl chloride (1.6 mL, 21.68 mmol) was added dropwise. The reaction mixture was then allowed to cool gradually over 18 h. Volatile components were removed under reduced pressure, and the residue was diluted with ethyl acetate. The organic phase was washed sequentially with a sodium bicarbonate solution (NaHCO_3_), water, and brine, dried over sodium sulfate (Na_2_SO_4_), and concentrated by evaporation. The ester obtained in spectroscopically pure form was used in the subsequent step without further purification. White solid; yield, 92%; m.p. 152°C–153°C. FT‐IR υ (cm^‐1^): 3410, 3319 (N–H), 3069 (Ar C–H), 2978–2907 (aliphatic C–H), 1687 (C = O), 1598 (C = N), 1514–1478 (Ar C = C), 1394, 1352 (aliphatic C–H, –CH_3_ and –CH_2_), 1368, 1027 (C–O), 1270, 1135 (C–N) (Supporting Information S1: Figure 56).

#### 5.3.2 Synthesis of ethyl 6‐(3‐benzoylthioureido)nicotinate (9)

Ethyl 6‑aminonicotinate **(8)** (830 mg, 5 mmol) and benzoyl isothiocyanate **(3)** (830 mg, 5.1 mmol) were dissolved in acetone and refluxed for 6 h. Subsequently, methanol was added to induce precipitation. The resulting solid was isolated by filtration, washed with methanol (MeOH), and dried to yield ethyl 6‑(3‑benzoyl‑thiourea)nicotinate **(9)** (Borzilleri et al. 2004). Light yellow solid; Yield, 86%; m.p.: 154°C–156°C; lit. m.p.: 150°C–152°C (Marzi et al. 2019). FT‐IR υ (cm^‐1^): 3275, 3092 (N–H), 3074–3000 (Ar C–H), 2981–2874 (aliphatic C–H), 1699 and 1669 (C = O), 1599 (C = N), 1509–1449 (Ar C = C), 1398, 1364 (aliphatic C–H, –CH_3_ and –CH_2_), 1320, 1019 (C–O), 1250 (C = S), 1241, 1149, 1113 (C–N) (Supporting Information S1: Figure 57).

#### 5.3.3 Synthesis of 6‐thioureidonicotinic acid (10)

Ethyl 6‐(3‐benzoyl‐thioureo)nicotinate **(9)** (1.35 g, 4.1 mmol) was refluxed in 5 mL of 2 N NaOH for 2 h. After completion of the reaction, the mixture was cooled to room temperature and acidified with 1 N HCl until the pH reached 4. The precipitated solid was collected by filtration, washed successively with water and methanol (MeOH), and dried to obtain 6‐thioureanicotinic acid **(10)**. White solid; yield, 68%; m.p.: 241°C–244°C; lit. m.p.: 245°C (Marzi et al. 2019). FT‐IR υ (cm^‐1^): 3420–3166 (N–H), 3221–2547 (C–O), 3091–3033 (Ar C–H), 1691 (C = O), 1588 (C = N), 1530–1419 (Ar C = C), 1307, 1056 (C–O), 1279 (C = S), 1231 (C–N) (Supporting Information S1: Figure 58).

#### 5.3.4 Synthesis of 6‐((4‐(ethoxycarbonyl)thiazole‐2‐yl)amino)nicotinic acid (Ca)

6‐((4‐(Ethoxycarbonyl)thiazole‐2‐yl)amino)nicotinic acid (**Ca**) was synthesized via the Hantzsch thiazole reaction. A mixture of 6‐thioureanicotinic acid **(10)** (550 mg, 2.8 mmol) and ethyl 3‐bromo‐2‐oxopropanoate (420 µL, 3.4 mmol) was stirred in anhydrous EtOH (3 mL) under microwave irradiation at 100°C for 70 min. Upon completion, the precipitate was collected by filtration, washed thoroughly with water and cold EtOH, and dried to afford the pure product. The reaction progress and product purity were monitored using thin‐layer chromatography (TLC) with TEF (5:4:1) as the mobile phase. White solid; yield, 71%; m.p. 331°C–332°C. FT‐IR υ (cm^‐1^): 3283–2547 (O–H), 3283 (N–H), 3116–3088 (Ar C–H), 2959–2824 (aliphatic C–H), 1724, 1683 (C = O), 1603 (C = N), 1525–1420 (Ar C = C), 1470, 1359 (aliphatic C–H, –CH_3_ and –CH_2_), 1371, 1293, 1097, 1021 (C–O), 1278, 1208, 1128 (C–N), 850 (C–S) (Supporting Information S1: Figure 59). ^1^H‐NMR (500 MHz, DMSO‐*d6*): δ 12.17 (s, 1H), 8.79 (d, *J* = 1.3 Hz, 1H), 8.12 (dd, *J* = 8.7, 2.0 Hz, 1H), 7.90 (s, 1H), 7,00 (d, *J* = 8.7 Hz, 1H), 4,21 (d, *J* = 7.1 Hz, 2H), 1.24 (t, *J* = 7.1 Hz, 3H) (Supporting Information S1: Figure 60). LC‐MS/MS (m/z): 294.150, [M + 1]^+^, 100.00%) (Supporting Information S1: Figure 61).

#### 5.3.5 General Method for the Synthesis of Intermediate C(b‐f)

6‐((4‐(Ethoxycarbonyl)thiazole‐2‐yl)amino)nicotinic acid (**Ca**) (210 mg, 0.7 mmol) and the corresponding amine (1.05 mmol) were dissolved in DCM (5 mL). Subsequently, 1‐ethyl‐3‐(3‐dimethylaminopropyl)carbodiimide hydrochloride (EDCI·HCl) (210 mg, 1.05 mmol) and 4‐dimethylaminopyridine (DMAP) (10 mg, 0.07 mmol) were added at 0°C, and the reaction mixture was stirred. The mixture was then allowed to warm to room temperature and subsequently washed with 1 N HCl (2 eq.) and brine (2 eq.). The organic phase was dried over MgSO_4_, filtered, and the solvent was removed under reduced pressure to yield the solid product.

##### 5.3.5.1 Ethyl‐2‐((5‐(morpholine‐4‐carbonyl)pyridin‐2‐yl)amino)thiazole‐4‐carboxylate (Cb)

Light yellow solid; yield, 70%; m.p. 207°C–209°C. FT‐IR υ (cm^‐1^): 3251 (N–H), 3119–3023 (Ar C–H), 2989–2858 (aliphatic C–H), 1725 and 1637 (C = O), 1599 (C = N), 1522–1429 (Ar C = C), 1499, 1361 (aliphatic C–H, –CH_3_ and –CH_2_), 1300, 1274, 1095, 1010 (C–O), 1227–1113 (C–N), 844 (C–S) (Supporting Information S1: Figure 62). LC‐MS/MS (m/z): 363.100, [M + 1]^+^, 100.00%) (Supporting Information S1: Figure 63).

##### 5.3.5.2 Ethyl‐2‐((5‐(piperidine‐1‐carbonyl)pyridin‐2‐yl)amino)thiazole‐4‐carboxylate (Cc)

White solid; yield, 75%; m.p. 100°C–101°C. FT‐IR υ (cm^‐1^): 3485, 3247 (N–H), 3108–2988 (Ar C–H), 2988–2864 (aliphatic C–H), 1694 and 1649 (C = O), 1591 (C = N), 1536–1468 (Ar C = C), 1468, 1381 (aliphatic C–H, –CH_3_ and –CH_2_), 1327, 1094 (C–O), 1278, 1246, 1140, 1122 (C–N), 846 (C–S) (Supporting Information S1: Figure 64). LC‐MS/MS (m/z): 361.100, [M + 1]^+^, 100.00%) (Supporting Information S1: Figure 65).

##### 5.3.5.3 Ethyl‐2‐((5‐((4‐(4‐methylpiperazin‐1‐yl)butyl)carbamoyl)pyridin‐2‐yl)amino)thiazole‐4‐carboxylate (Cd)

White solid; yield, 67%; m.p. 188°C–189°C. FT‐IR υ (cm^‐1^): 1726 and 1635 (C = O), 1602 (C = N), 1522–1490 (Ar C = C), 1438, 1371 (aliphatic C–H, –CH_3_ and –CH_2_), 1285, 1094 (C–O), 1227, 1198, 1163 (C–N), 843 (C–S) (Supporting Information S1: Figure 66). LC‐MS/MS (m/z): 447.200, [M + 1]^+^, 100.00%) (Supporting Information S1: Figure 67).

##### 5.3.5.4 Ethyl‐2‐((5‐((4‐(piperidin‐1‐yl)butyl)carbamoyl)pyridin‐2‐yl)amino)thiazole‐4‐carboxylate (Ce)

White solid; yield, 63%; m.p. 199°C–201°C. FT‐IR υ (cm^‐1^): 3250, 3172 (N–H), 3119–2805 (aromatic and aliphatic C–H), 1727 and 1636 (C = O), 1602 (C = N), 1522–1490 (aromatic C = C), 1438, 1369 (aliphatic C–H, –CH_3_ and –CH_2_), 1293, 1094 (C–O), 1225, 1197, 1154 (C–N), 843 (C–S) (Supporting Information S1: Figure 68). LC‐MS/MS (m/z): 432.250, [M + 1]^+^, 100.00%) (Supporting Information S1: Figure 69).

##### 5.3.5.5 Ethyl‐2‐((5‐((4‐morpholinobutyl)carbamoyl)pyridin‐2‐yl)amino)thiazole‐4‐carboxylate (Cf)

White solid; yield, 59%; m.p. 207°C–208°C. FT‐IR υ (cm^‐1^): 3268, 3172 (N–H), 3119–2812 (aromatic and aliphatic C–H), 1723 and 1631 (C = O), 1602 (C = N), 1521–1490 (aromatic C = C), 1438, 1368 (aliphatic C–H, –CH_3_ and –CH_2_), 1294, 1094 (C–O), 1225, 1203, 1116 (C–N), 844 (C–S) (Supporting Information S1: Figure 70). LC‐MS/MS (m/z): 434.200, [M + 1]^+^, 100.00%) (Supporting Information S1: Figure 71).

#### 5.3.6 General Method for the Synthesis of Intermediates C(g‐k)

**Method a**. A 10% w/v aqueous KOH solution (5 mL) was added to a solution of intermediate C(g and h) (0.5 mmol) in a 10 mL mixture of THF:EtOH (1:1). The reaction mixture was stirred at room temperature until the starting material was completely consumed, as monitored by TLC on a SiO_2_ plate. The mixture was then acidified with 1 N HCl to adjust the pH to 4, and the resulting solid was filtered. Finally, the acid was recrystallized from acetone to yield the desired product.

**Method b**. A 10% w/v aqueous KOH solution (5 mL) was added to a solution of intermediate C(i‐k) (0.5 mmol) in 10 mL of a THF:EtOH (1:1) mixture. The reaction mixture was stirred at room temperature until the starting material was completely consumed, as monitored by thin‐layer chromatography (TLC) on silica gel plates. The mixture was then acidified with 1 N HCl to adjust the pH to 4, and the aqueous phase was extracted with ethyl acetate. The organic phase was washed three times with water, dried over Na_2_SO_4_, and concentrated using a rotary evaporator to remove the ethyl acetate. impure, viscous liquid was obtained and carried forward to the next step.

##### 5.3.6.1 2‐((5‐(Morpholine‐4‐carbonyl)pyridin‐2‐yl)amino)thiazole‐4‐carboxylic acid (Cg)

Light yellow solid; yield, 75%; m.p. 288°C–289°C. FT‐IR υ (cm^‐1^): 3246 (N–H), 3424–2500 (O–H), 3088 (Ar C–H), 2961–2851 (aliphatic C–H), 1716, 1677 (C = O), 1599 (C = N), 1542–1428 (aromatic C = C), 1383 (aliphatic C–H, –CH_2_), 1306, 1276, 1099, 1010 (C–O), 1237, 1212, 1163 (C–N), 900 (C–S) (Supporting Information S1: Figure 72). LC‐MS/MS (m/z): 335.150, [M + 1]^+^, 100.00% (Supporting Information S1: Figure 73).

##### 5.3.6.2 2‐((5‐(Piperidine‐1‐carbonyl)pyridin‐2‐yl)amino)thiazole‐4‐carboxylic acid (Ch)

White solid; yield, 90%; m.p. 289°C–290°C. FT‐IR υ (cm^‐1^): 3248 (N–H), 3636–2516 (O–H), 3087 (Ar C–H), 2937–2854 (aliphatic C–H), 1716, 1678 (C = O), 1600 (C = N), 1534–1431 (aromatic C = C), 1378 (aliphatic C–H, –CH_2_), 1311, 1099 (C–O), 1271, 1235, 1213 (C–N), 901 (C–S) (Supporting Information S1: Figure 74). LC‐MS/MS (m/z): 333.050, [M + 1]^+^, 100.00% (Supporting Information S1: Figure 75).

#### 5.3.7 General Procedure for the Synthesis of Target Compounds (C10‐C15)

**Method a.** In a solution of DMF (4 mL) and DCM (5 mL), intermediate compounds (Cg and Ch) (0.3 mmol) was dissolved at room temperature, and then 1,1’‐carbonyldiimidazole (CDI) (81 mg, 0.5 mmol) and thiazol‐2‐amine (40 mg, 0.4 mmol) were added. The reaction mixture was stirred at room temperature for 30 min and then heated at 70°C for 3 h. The progress of the reaction and the purity of the compound were monitored by TLC on a SiO_2_ plate using TEF (5:4:1) as the mobile phase. Upon completion, the reaction mixture was poured into a saturated ammonium chloride solution. The mixture was extracted with DCM (3 × 20 mL), and the combined organic layers were dried over Na_2_SO_4_ and concentrated under reduced pressure. The resulting product was crystallized from methanol and washed with diethyl ether (Et_2_O) to afford **(C10** and **C11)**. Compound **C15** was synthesized following the same preparation and purification procedures; however, the reaction was conducted at room temperature for 24 h. Under these modified conditions, compound **C15** was obtained as the final product.

**Method b**. In a solution of DMF (7 mL) and DCM (10 mL), intermediate compounds C(i‐k) were dissolved at room temperature. To this solution, CDI (200 mg, 1.2 mmol) and thiazol‐2‐amine (100 mg, 1 mmol) were added. The reaction mixture was stirred at room temperature for 30 min and then heated at 70°C for 3 h. The progress of the reaction and the purity of the compound were monitored by TLC on silica gel plates using TEF (5:4:1) as the mobile phase. After the reaction, the DCM was removed under reduced pressure using a rotary evaporator. The reaction mixture was then poured into ice‐cold water, and the resulting solid was collected by filtration. The crude product was recrystallized from methanol and washed with diethyl ether to afford **C12‑C14**.

##### 5.3.7.1 2‐((5‐(Morpholine‐4‐carbonyl)pyridin‐2‐yl)amino)‐N‐(thiazole‐2‐yl)thiazole‐4‐carboxamide (C10)

**Method a.** Cream solid; yield, 44%; m.p. 286°C–287°C. FT‐IR υ (cm^‐1^): 3349, 3245 (N–H), 3109–3034 (Ar C–H str.), 2968–2870 (aliphatic C–H), 1702, 1685 (C = O), 1600 (C = N), 1525–1447 (Ar C = C), 1373 (aliphatic C–H, –CH_2_), 1302, 1028 (C–O), 1268–1105 (C–N), 884 (C–S) (Supporting Information S1: Figure 76). ^1^H NMR (500 MHz, DMSO‐*d6*): δ 11.91 (s, 1H), 11.61 (s, 1H), 8.43 (d, *J* = 2.4 Hz, 1H), 8.09 (s, 1H), 7.84 (dd, *J* = 8.5, 2.4 Hz, 1H), 7.55 (d, *J* = 3.4 Hz, 1H), 7.31 (d, *J* = 3.5 Hz, 1H), 7.16 (d, *J* = 8.5 Hz, 1H), 7.15 (d, *J* = 8.6 Hz, 1H), 3.68–3.60 (m, 4H), 3.55–3.47 (m, 4H) (Supporting Information S1: Figure 77). No dominant C NMR peaks were observed. LC‐MS/MS (m/z): 417.100 ([M + 1]^+^; 100.00%) (Supporting Information S1: Figure 78).

##### 5.3.7.2 2‐((5‐(Piperidine‐1‐carbonyl)pyridin‐2‐yl)amino)‐N‐(thiazole‐2‐yl)thiazole‐4‐carboxamide (C11)

**Method a.** White solid; yield, 36%; m.p. 172°C–173°C. FT‐IR υ (cm^‐1^): 3490, 3279 (N–H), 3100–2988 (Ar C–H), 2941–2857 (aliphatic C–H), 1655 (C = O str.), 1595 (C = N str.), 1541–1438 (Ar C = C), 1382 (aliphatic C–H, –CH_2_), 1281–1160 (C–N str.), 885 (C–S) (Supporting Information S1: Figure 79). ^1^H NMR (400 MHz, CDCl_3_): δ 10.59 (s, 1H), 9.25 (s, 1H), 8.51 (d, *J* = 2.3 Hz, 1H), 7.84 (s, 1H), 7.77 (dd, *J* = 8.7, 2.3 Hz, 1H), 7.52 (d, *J* = 3.7 Hz, 1H), 7.08 (d, *J* = 8.2 Hz, 1H), 7.03 (d, *J* = 3.2 Hz, 1H), 3.47 (q, *J* = 6.9 Hz, 4H), 1.20 (t, *J* = 6.9 Hz, 4H), 1.09 (s, 2H) (Supporting Information S1: Figure 80). ^13^C NMR (100 MHz, CDCl_3_): δ 167.99, 160.04, 159.06, 158.38, 151.92, 146.38, 142.51, 137.74, 137.63, 125.06, 119.29, 114.13, 110.24, 65.98, 24.61, 15.39 (Supporting Information S1: Figure 81). LC‐MS/MS (m/z): 383.150 ([M + 1]^+^; 100.00%) (Supporting Information S1: Figure 82).

##### 5.3.7.3 2‐((5‐((4‐(4‐Methylpiperazin‐1‐yl)butyl)carbamoyl)pyridin‐2‐yl)amino)‐N‐(thiazole‐2‐yl)thiazole‐4‐carboxamide (C12)

**Method b.** Cream solid; m.p.: 262°C–264°C. FT‐IR υ (cm^‐1^): 3410, 3224 (N–H str.), 3095 (Ar C–H), 2989–2945 (aliphatic C–H), 1667 and 1631 (C = O), 1600 (C = N), 1531–1480 (Ar C = C), 1376 (aliphatic C–H, –CH_2_), 1319, 1278, 1259, 1208, 1163 (C–N), 845 (C–S) (Supporting Information S1: Figure 83). ^1^H NMR (500 MHz, DMSO‐*d6*): δ 11.94 (s, 1H), 11.55 (s, 1H), 8.82 (d, *J* = 2.4 Hz, 1H), 8.46 (s, 1H), 8.15 (dd, *J* = 8.6, 2.4 Hz, 1H), 8.12 (s, 1H), 7.55 (d, *J* = 3.5 Hz, 1H), 7.31 (d, *J* = 3.5 Hz, 1H), 7.14 (d, *J* = 8.7 Hz, 1H), 3.31–3.23 (m, 4H), 2.40–2.20 (m, 8H), 2.14 (s, 3H), 1.65–1.34 (m, 4H) (Supporting Information S1: Figure 84). ^13^C NMR (125 MHz, DMSO‐*d6*): δ 164.80, 159.81, 159.38, 158.04, 153.32, 146.63, 142.70, 138.35, 137.72, 123.43, 119.33, 114.61, 110.66, 57.99, 55.20, 53.12, 39.40, 46.19, 27.54, 24.32 (Supporting Information S1: Figure 85). LC‐MS/MS (m/z): 501.250 ([M + 1]^+^; 100.00% (Supporting Information S1: Figure 86).

##### 5.3.7.4 2‐((5‐((4‐(Piperidin‐1‐yl)butyl)carbamoyl)pyridin‐2‐yl)amino)‐N‐(thiazole‐2‐yl)thiazole‐4‐carboxamide (C13)

**Method b.** White solid; m.p. 155°C–157°C. FT‐IR υ (cm^‐1^): 3376, 3226, 3152 (N–H str.), 3079 (Ar C–H), 2983–2831 (aliphatic C–H), 1672 and 1632 (C = O), 1603 (C = N), 1532–1476 (Ar C = C), 1376 (aliphatic C–H, –CH_2_), 1309, 1283, 1272, 1205, 1153 (C–N), 846 (C–S) (Supporting Information S1: Figure 87). ^1^H NMR (500 MHz, DMSO‐*d6*): δ 11.97 (s, 1H), 11.55 (s, 1H), (d, *J* = 2.3 Hz, 1H), 8.58 (s, 1H), 8.17 (d, *J* = 8.6 Hz, 1H), 8.13 (s, 1H), 7.55 (d, *J* = 3.6 Hz, 1H), 7.31 (d, *J* = 3.5 Hz, 1H), 7.15 (d, *J* = 8.7 Hz, 1H), 3.30 (s, 4H), 3.03–2.82 (m, 4H), 1.83–1.48 (m, 10H) (Supporting Information S1: Figure 88). ^13^C NMR (125 MHz, DMSO‐*d6*): δ 164.97, 159.78, 159.37, 153.41, 146.70, 142.72, 138.43, 137.75, 123.27, 119.31, 114.62, 110.71, 56.06, 52.54, 38.86, 26.82, 23.02, 21.92, 21.35 (Supporting Information S1: Figure 89). LC‐MS/MS (m/z): 486.200, 487.250 ([M + 1]^+^, [M + 2]^+^; 100.00%, 100.00% (Supporting Information S1: Figure 90).

##### 5.3.7.5 2‐((5‐((4‐Morpholinobutyl)carbamoyl)pyridin‐2‐yl)amino)‐N‐(thiazole‐2‐yl)thiazole‐4‐carboxamide (C14)

**Method b.** Cream solid; m.p. 301°C–303°C. FT‐IR υ (cm^‐1^): 3387, 3233, 3160 (N–H), 3079–3017 (Ar C–H str.), 2950–2835 (aliphatic C–H), 1685 and 1634 (C = O), 1605 (C = N), 1531–1477 (Ar C = C), 1376 (aliphatic C–H, –CH_2_), 1322, 1112 (C–O), 1272, 1259, 1190, 1122, 1107 (C–N), 852 (C–S) (Supporting Information S1: Figure 91). ^1^H NMR (500 MHz, DMSO‐*d6*): δ 12.00 (s, 2H), 8.88 (d, *J* = 2.4 Hz, 1H), 8.55–8.52 (m, 1H), 8.21 (dd, *J* = 8.6, 2.4 Hz, 1H), 8.18 (s, 1H), 7.62 (d, *J* = 3.5 Hz, 1H), 7.37 (d, *J* = 3.5 Hz, 1H), 7.20 (d, *J* = 8.5 Hz, 1H), 3.62 (t, *J* = 4.7 Hz, 4H), 3.34 (q, *J* = 6.9 Hz, 4H), 2.45–2.40 (m, 2H), 2.34 (t, *J* = 7.2 Hz, 2H), 1.64–1.50 (m, 4H) (Supporting Information S1: Figure 92). ^13^C NMR (125 MHz, DMSO‐*d6*): δ 177.21, 159.82, 153.33, 146.63, 142.72, 137.72, 129.46, 126.84, 123.42, 114.60, 110.66, 101.86, 66.68, 58.43, 53.84, 29.17, 27.48, 23.94 (Supporting Information S1: Figure 93). LC‐MS/MS (m/z): 488.200 [M + 1]^+^, 100.00% (Supporting Information S1: Figure 94).

##### 5.3.7.6 (6‐((4‐(1H‐Imidazol‐1‐carbonyl)thiazole‐2‐yl)amino)pyridin‐3‐yl)(piperidin‐1‐yl)methanone (C15)

**Method a.** Cream solid; yield, 55%; m.p. 180°C–181.5°C. FT‐IR υ (cm^‐1^): 3229, 3148 (N–H), 3105, 3015 (Ar C–H), 2939–2862 (aliphatic C–H), 1698 (C = O), 1610 (C = N), 1538–1428 (Ar C = C str.), 1369 (aliphatic C–H, –CH_2_), 1271, 1221, 1135 (C–N), 904 (C–S) (Supporting Information S1: Figure 95). ^1^H NMR (500 MHz, DMSO‐*d6*): δ 11.91 (s, 1H), 9.02 (s, 1H), 8.40 (sd, *J* = 2.0 Hz, 1H), 8.32 (s, 1H), 8.04 (s, 1H), 7.81 (dd, *J* = 8.5, 2.2 Hz, 1H), 7.15–7.11 (m, 2H), 3.30 (s, 4H), 1.65–1.46 (m, 6H) (Supporting Information S1: Figure 96). ^13^C NMR (125 MHz, DMSO‐*d6*): δ 167.19, 160.05, 158.43, 152,18. 145.72, 141.70, 139.64, 137.96, 130.25, 126.14, 125.30, 118.67, 110.88, 40.03, 24.47 (Supporting Information S1: Figure 97). LC‐MS/MS (m/z): 383.150 ([M + 1]^+^; 100.00%) (Supporting Information S1: Figure 98).

### 5.4 Computational Studies

#### 5.4.1 ADMET/RO5 and Toxicity Predictions

Pharmacokinetic and druggability properties of the synthesized compounds were evaluated using the Discovery Studio (DS). ADMET (Absorption, Distribution, Metabolism, Excretion, and Toxicity) predictions were performed using the ADMET descriptor module. Additionally, drug‐likeness was assessed based on Lipinski's rule of five (RO5) and Veber's rules to ensure compliance with established criteria for orally active compounds (Kalin and Comert Onder 2025; Abusharkh et al. 2024b).

#### 5.4.2 Molecular Docking

The structures of the synthesized compounds **(C1–C15)** were drawn and subsequently converted to SDF format using Chem3D. These files were then converted into PDB format utilizing OpenBabel to facilitate molecular docking studies (O'Boyle et al. 2011). Ligand preparation was conducted using AutoDock Tools (ADT) from the MGL software suite (Abusharkh et al. 2024a; Morris et al. 2009). Gasteiger partial charges were assigned to each ligand, and the structures were saved in a rigid format. To ensure compatibility with docking simulations, all ligand structures were converted into PDBQT format for further computational analyses (Morris et al. 2009; Sirakanyan et al. 2015; Trott and Olson 2010). The crystal structure of FOXM1 (PDB ID: 3G73) was retrieved from the Protein Data Bank (PDB) (www.pdb.org) and preprocessed to optimize it for molecular docking studies (Abusharkh et al. 2024a; Zhang et al. 2022; Luo et al. 2021). Initially, all heteroatoms, water molecules, and chains A, C, and D were removed from the crystal structure. Subsequently, polar hydrogen atoms were added to the remaining protein structure. The processed structure was then saved in PDBQT format to ensure compatibility with docking protocols (Abusharkh et al. 2024a; O'Boyle et al. 2011; Jorgensen and Tirado‐Rives 1988). Grid box preparation was performed using AutoDock Tools version 1.5.7, following standard protocols (Abusharkh et al. 2024a, 2024b; Sirakanyan et al. 2015; Morris et al. 2009). The binding site residues of the FOXM1 DNA‐binding domain (DBD) were identified to define the docking region. Crucial residues, including Asn283, Arg286, and His287, were found to play a pivotal role in facilitating the interaction between FOXM1 and DNA (Abusharkh et al. 2024a, 2024b; Littler et al. 2010; Zhang et al. 2022; Luo et al. 2021; Trott and Olson 2010). Accordingly, the grid was centered on the ligand‐binding region within the FOXM1‐DBD domain.

Molecular docking studies were executed using AutoDock Vina to predict the binding conformations and interaction affinities of the synthesized compounds. Following docking, receptor‐ligand interaction analyses were conducted using the DS software package to visualize and interpret binding interactions.

#### 5.4.3 MD Simulation

The dynamic behavior of the protein‐ligand complex was examined using the Desmond molecular dynamics (MD) simulation package integrated within Schrödinger Maestro. Prior to simulation, the FOXM1 protein structure was prepared using the Protein Preparation Wizard to ensure structural integrity and optimization. To mimic physiological conditions, the protein‐ligand complex was solved in an orthorhombic periodic box filled with simple point charge (SPC) water molecules. System neutralization was achieved by adding a 150 mM NaCl solution to maintain an appropriate ionic environment (Kalin and Comert Onder 2025).

The system was subjected to energy minimization using the OPLS2005 force field to remove steric clashes and optimize atomic interactions (Jorgensen and Tirado‐Rives 1988). Following minimization, the system was equilibrated and subsequently restrained to its initial position. MD simulations were carried out for 100 ns under constant temperature (300 K) and pressure (1 atm) using the NPT ensemble (Abusharkh et al. 2024a; 2024b; Sirakanyan et al. 2015; Morris et al. 2009). The trajectory data obtained from the simulations were analyzed to assess the stability and conformational changes of the protein‐ligand complex over time. Additionally, key molecular interactions, including hydrogen bonds, hydrophobic interactions, ionic interactions, and water bridges, were examined to elucidate the binding mechanism.

### 5.5 In Vitro Activity Studies

#### 5.5.1 Cell Lines and Culture Conditions

The human TNBC cell lines (BT‐549, BT‐20 and MDA‐MB‐231) from the American Type Culture Collection (ATCC, Manassas, VA, USA) were used. The cells were maintained in a Dulbecco's Modified Eagle's Medium (DMEM)/F12 mixture, supplemented with 10% fetal bovine serum (FBS) (Sigma‐Aldrich, St. Louis, MO). To ensure optimal growth conditions, the cells were cultured under standard conditions at 37°C with 5% CO₂ in a humidified incubator (Sirakanyan et al. 2015). The culture medium (DMEM/F12, 1:1) was further supplemented with 10% FBS and 1% penicillin/streptomycin (PS) to support cell viability (Abusharkh et al. 2024a, 2024b; Cınar et al. 2022; Comert Onder et al. 2021b).

#### 5.5.2 Cell Cytotoxicity Assay

To assess the cytotoxic effects of the synthesized compounds, the cells were treated with the compounds at the increasing concentrations ranging from 5 µM to 100 µM for 96 h. DMSO was used as a control group. After the incubation period, MTT solution (3‐(4,5‐dimethylthiazole‐2‐yl)‐2,5‐diphenyltetrazolium bromide) was added into each well, followed by 3 h incubation at 37°C in a CO_2_ incubator, (Abusharkh et al. 2024a; Cınar et al. 2022; Comert Onder et al. 2021b). The resulting formazan crystals were solubilized in DMSO, and the absorbance was measured at 570 nm using a Thermo Spectrophotometer. The experimental data was processed and analyzed using GraphPad Prism 7 software to determine the percentage of cell viability.

#### 5.5.3 Western Blotting

Western blot analysis was performed according to the method described in our previous publications (Abusharkh et al. 2024a, 2024b; Cınar et al. 2022; Comert Onder et al. 2021a). MDA‐MB‐231 and BT‐549 cells were treated with varying concentrations of the potent compound **C11** (7.5 µM, 10 µM, and 20 µM for MDA‐MB‐231 cells; 5 µM, 10 µM, and 20 µM for BT‐549 cells) over a 96h period. Following treatment, cells were collected and washed with phosphate‐buffered saline (PBS). Protein lysates were extracted using a lysis buffer containing phosphatase and protease inhibitors, as well as 1% NaCl. Protein concentrations were determined using the BCA assay kit. After SDS‐PAGE, proteins were transferred to a membrane and probed with primary antibodies (FOXM1 and *β*‐actin (Proteintech)). The membranes were then washed with TBS‐T, incubated with the corresponding secondary antibodies (anti‐rabbit and anti‐mouse), and the protein signals were visualized (Cınar et al. 2022).

#### 5.5.4 Statistical Analysis

Statistical analyses of the repeated experiments were performed using Welch's t‐test in GraphPad Prism 8.0. Results with *P*‐values less than 0.05 were considered statistically significant and are indicated with an asterisk.

## Author Contributions

**Khaled A. N. Abusharkh:** conceptualization, methodology, investigation, validation, formal analysis, writing – original draft. **Venhar Cinar:** investigation, validation. **Alper Onder:** investigation, methodology, writing – original draft. **Merve Sıkık:** investigation, validation. **Mustafa Guzel:** writing – review and editing. **Zuhal Hamurcu:** investigation, methodology, resources, writing – review and editing. **Bulent Ozpolat:** writing – review and editing. **Mehmet Ay:** supervision, conceptualization, investigation, methodology, resources, writing – review and editing. **Ferah Comert Onder:** supervision, conceptualization, investigation, methodology, resources, software, project administration, writing – review and editing.

## Conflicts of Interest

The authors declare no conflicts of interest.

## Supporting information

Supporting File

## Data Availability

The data that support the findings of this study are available in the Supporting material of this article.
