# Supplementary material for: Design, Synthesis, Biological Evaluation, and Molecular Modeling Studies of Novel 2‐Aminothiazole Derivatives as Potential FOXM1 Inhibitors for Triple‐Negative Breast Cancer Therapy and Structure‐Activity Relationship
Source: Drug Dev Res. 2026 May 3;87:e70296. doi: 10.1002/ddr.70296 (PMC13135722; doi:10.1002/ddr.70296)
Supplement: Supplementary file 1 — Supporting File [file DDR-87-e70296-s001.pdf]

Supporting Information

Design, synthesis, biological evaluation, and molecular modeling studies of novel 2-aminothiazole derivatives as potential FOXM1 inhibitors for triple-negative breast cancer therapy and structure-activity relationship

Khaled A.N. Abusharkh[1-3], Venhar Çınar[4], Alper Onder[2], Merve Sıkık[5], Mustafa Guzel[6-8], Zuhal Hamurcu[4,*], Bulent Ozpolat[9,10,*], Mehmet Ay[2,*], Ferah Comert Onder[11,*]

[1]Çanakkale Onsekiz Mart University, School of Graduate Studies, Department of Chemistry, Çanakkale, Türkiye

[2]Çanakkale Onsekiz Mart University, Faculty of Science, Department of Chemistry, Natural Products and Drug Research Laboratory, Çanakkale, Türkiye

[3]Al-Quds University, Faculty of Science and Technology, Department of Chemistry and Chemical Technology, East Jerusalem, Palestine

[4] Erciyes University, Faculty of Medicine, Department of Medical Biology, Kayseri, Türkiye

[5] Çanakkale Onsekiz Mart University, School of Graduate Studies, Department of Medical System Biology, Türkiye

[6]Research Institute for Health Sciences and Technologies (SABITA), Center of Drug Discovery and Development, Istanbul Medipol University, Kavacik-Beykoz, İstanbul, 34810, Türkiye

[7]Department of Medical Pharmacology, International School of Medicine, Istanbul Medipol University, Kavacik Campus, Kavacik-Beykoz, İstanbul, 34810, Türkiye

[8]Department of Basic Pharmaceutical Sciences, School of Pharmacy, İstanbul Medipol University, 34810 Beykoz-İstanbul, Türkiye

[9]Department of Nanomedicine, Houston Methodist Research Institute, Houston, TX, USA

[10]Stephenson School of Biomedical Engineering, The University of Oklahoma, Norman, OK 73019, USA

[11]Çanakkale Onsekiz Mart University, Faculty of Medicine, Department of Medical Biology, Çanakkale, Türkiye

Corresponding authors. E-mail addresses ferahcomertonder@comu.edu.tr (FCO); mehmetay06@comu.edu.tr (MA); zhamurcu@erciyes.edu.tr (ZH), bozpolat@ou.edu (BO).

Table of Contents

Absorption, Distribution, Metabolism, Excretion, and Toxicity (ADMET)/Lipinski's Rule of Five (RO5) and Toxicity Predictions…………………………………………………….…………..3-4

Table S3. Docking scores (kcal/mol) and 2D interaction diagrams of the synthesized 2-aminothiazole derivatives (**C1-C15**) with FOXM1-DBD..5-8

Spectroscopic Characterization Data

Figure S1-S98 9-59

Absorption, Distribution, Metabolism, Excretion, and Toxicity (ADMET)/Lipinski's Rule of Five (RO5) and Toxicity Predictions

Table S1. Summarizes the ADMET/RO5 analyses for the synthesized compounds **C1-C15**.

No	Compound	Molecular Weight (g/mol)	AlogP98	Solubility	CYP2D6	Hepato-toxicity	BBB	HIA	PPB
**1**	**C1**	329.463	5.481	1	False	False	0	0	True
**2**	**C2**	290.379	4.731	2	False	True	0	0	True
**3**	**C3**	340.386	5.468	1	True	True	0	0	True
**4**	**C4**	351.285	5.274	1	False	True	0	0	True
**5**	**C5**	302.414	4.509	2	False	True	0	0	True
**6**	**C6**	308.369	4.936	1	False	True	0	0	True
**7**	**C7**	286.415	5.012	2	False	True	0	0	True
**8**	**C8**	352.273	4.662	2	False	True	0	0	True
**9**	**C9**	352.273	4.662	2	False	True	0	0	True
**10**	**C10**	416.477	1.464	3	False	True	3	0	False
**12**	**C11**	414.505	2.693	3	False	True	3	0	False
**11**	**C12**	499.652	3.56	2	False	True	4	0	False
**13**	**C13**	485.625	3.268	2	False	False	4	0	False
**14**	**C14**	487.598	2.039	3	False	False	4	0	False
**15**	**C15**	382.440	1.733	2	False	True	3	0	False

Solubility is rated on a scale where 0 denotes very low solubility, 1 indicates very low but feasible solubility, 2 represents low solubility, and 3 signifies good solubility. Blood–Brain Barrier (BBB) permeability is classified as 0 for very high permeability, 1 for high, 2 for moderate, 3 for low, and 4 for undefined. The Cytochrome P450 2D6 status is indicated as “True” for inhibitors and “False” for non-inhibitors, while hepatotoxicity is similarly marked (“True” for inhibitors, “False” for non-inhibitors). Human Intestinal Absorption (HIA) is evaluated on a scale from 0 (good) to 3 (very low), and Plasma Protein Binding (PPB) is reported as “False” for weak binding and “True” for strong binding. Finally, an AlogP98 value below 5 is considered indicative of ideal cell permeability.

Table S2. *In silico* toxicity predictions.

No	Compound	RAT NTP		MOUSE NTP	
		**MALE**	**FEMALE**	**MALE**	**FEMALE**
1	**C1**	Carcinogen	Carcinogen	Carcinogen	Non-carcinogen
2	**C2**	Non-carcinogen	Non-carcinogen	Non-carcinogen	Carcinogen
3	**C3**	Non-carcinogen	Non-carcinogen	Non-carcinogen	Non-carcinogen
4	**C4**	Carcinogen	Non-carcinogen	Carcinogen	Non-carcinogen
5	**C5**	Non-carcinogen	Non-carcinogen	Non-carcinogen	Non-carcinogen
6	**C6**	Non-carcinogen	Non-carcinogen	Non-carcinogen	Non-carcinogen
7	**C7**	Non-carcinogen	Non-carcinogen	Non-carcinogen	Non-carcinogen
8	**C8**	Non-carcinogen	Non-carcinogen	Non-carcinogen	Non-carcinogen
9	**C9**	Non-carcinogen	Non-carcinogen	Non-carcinogen	Non-carcinogen
10	**C10**	Non-carcinogen	Non-carcinogen	Non-carcinogen	Non-carcinogen
11	**C11**	Non-carcinogen	Non-carcinogen	Non-carcinogen	Non-carcinogen
12	**C12**	Non-carcinogen	Non-carcinogen	Non-carcinogen	Non-carcinogen
13	**C13**	Non-carcinogen	Non-carcinogen	Non-carcinogen	Non-carcinogen
14	**C14**	Non-carcinogen	Non-carcinogen	Non-carcinogen	Non-carcinogen
15	**C15**	Non-carcinogen	Non-carcinogen	Non-carcinogen	Non-carcinogen

Table S3. Docking scores (kcal/mol) and 2D interaction diagrams of the synthesized 2-aminothiazole derivatives (C1-C15) with FOXM1-DBD

No.	Compound Code	2D interaction diagram	Docking score (kcal/mol)
		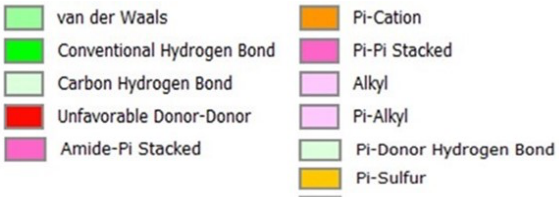	
1	C1	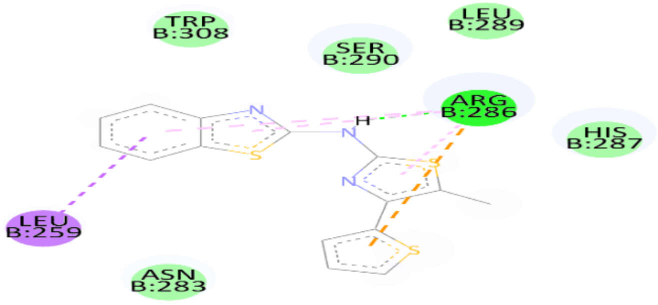	-5.1
2	C2	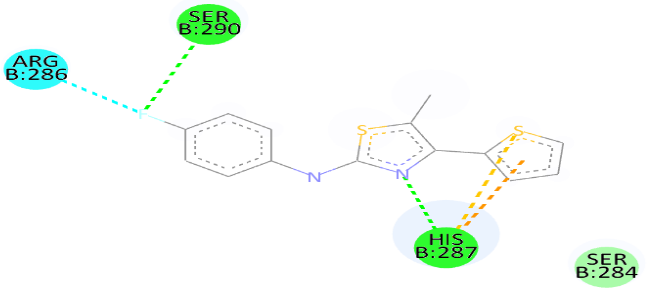	-4.9
3	C3	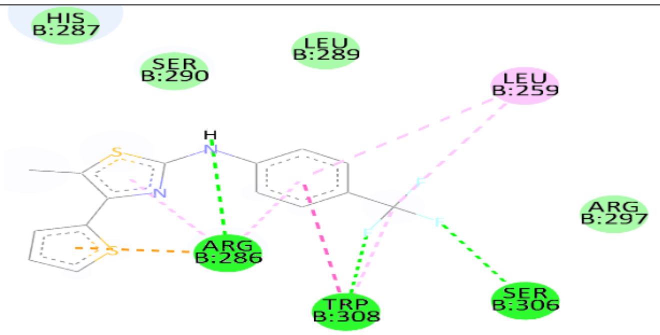	-5.4

**4**	**C4**	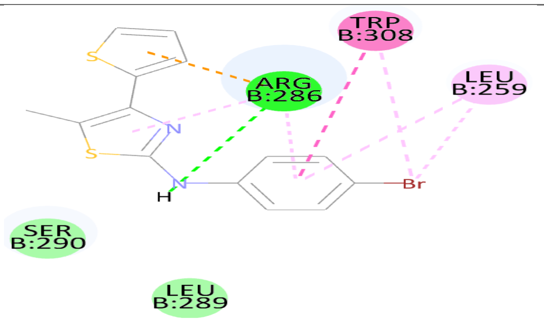	**-4.8**
**5**	**C5**	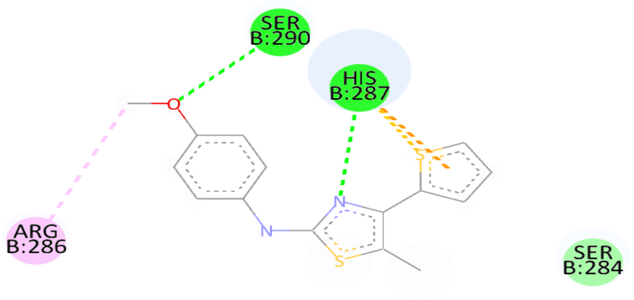	**-4.9**
**6**	**C6**	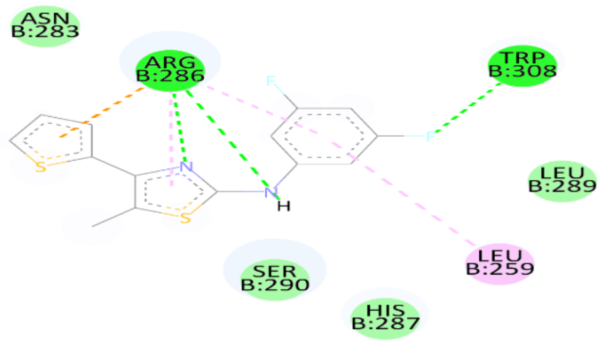	**-5.1**
**7**	**C7**	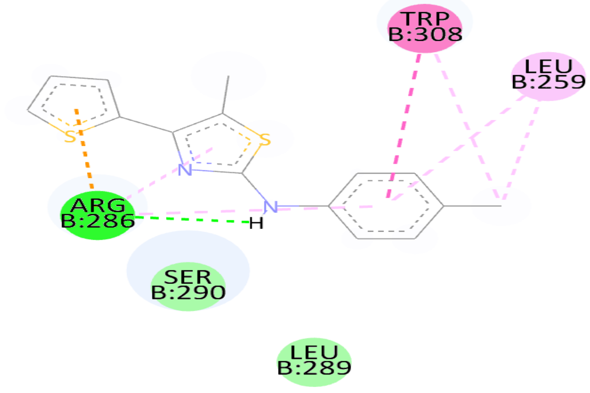	**-5.0**

**8**	**C8**	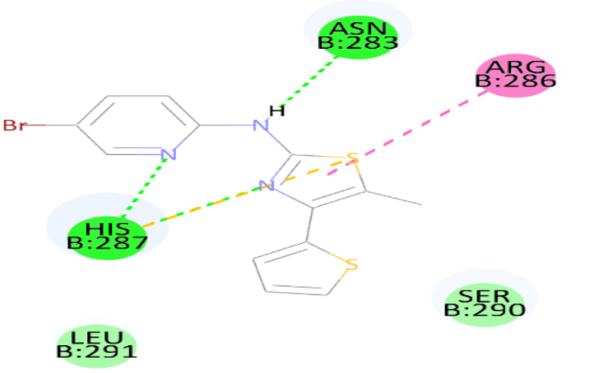	**-4.6**
**9**	**C9**	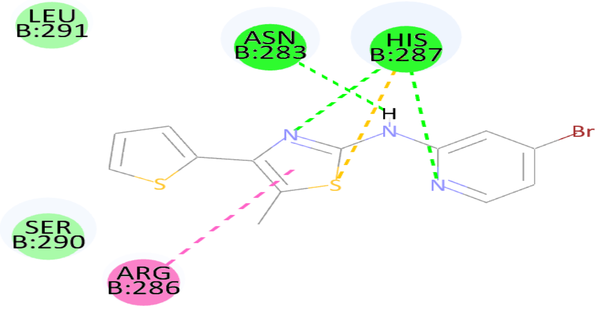	**-4.7**
**10**	**C10**	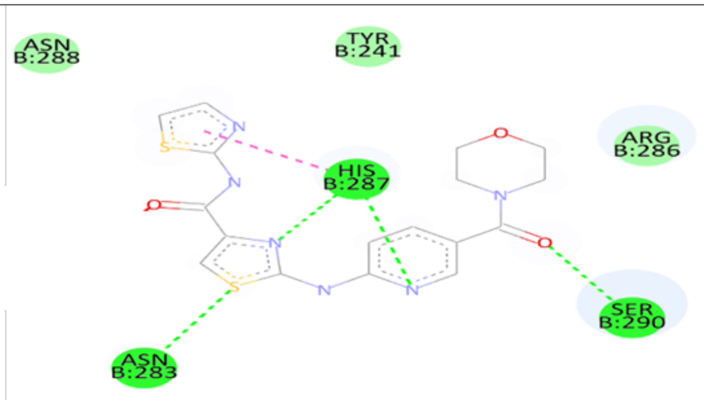	**-6.0**
**11**	**C11**	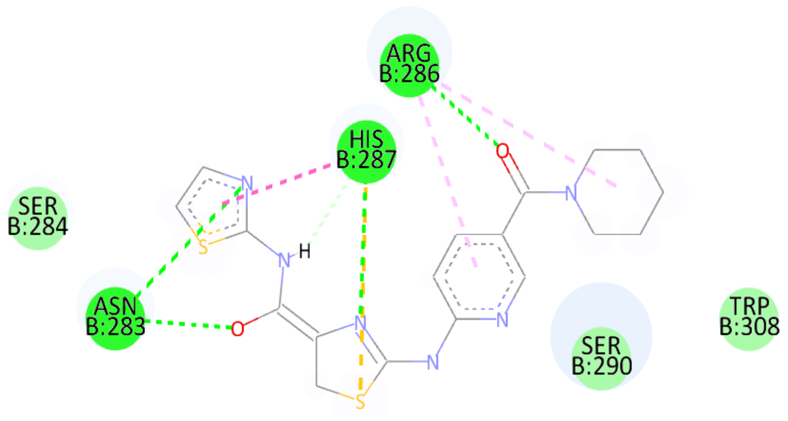	**-6.1**

**12**	**C12**	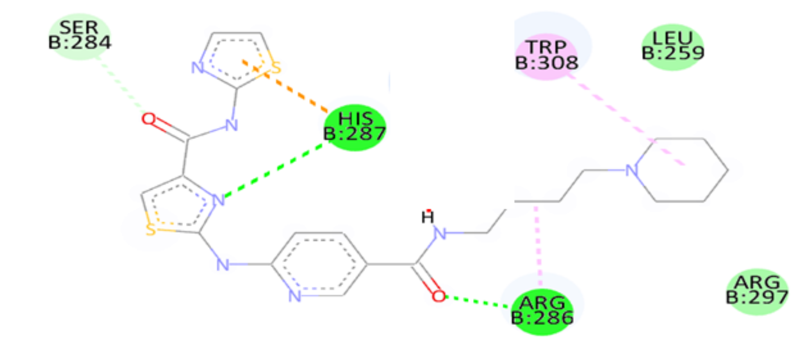	**-6.4**
**13**	**C13**	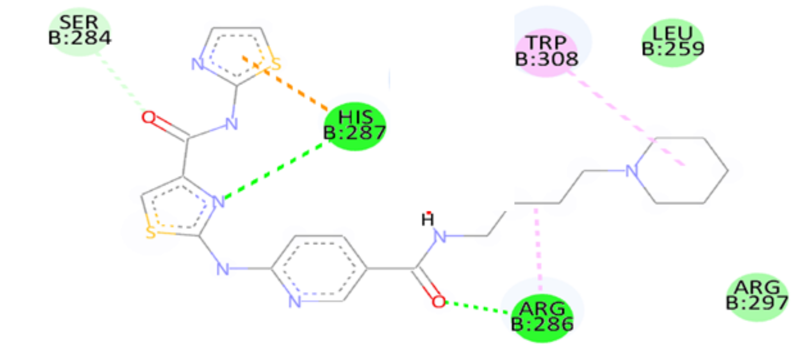	**-6.6**
**14**	**C14**	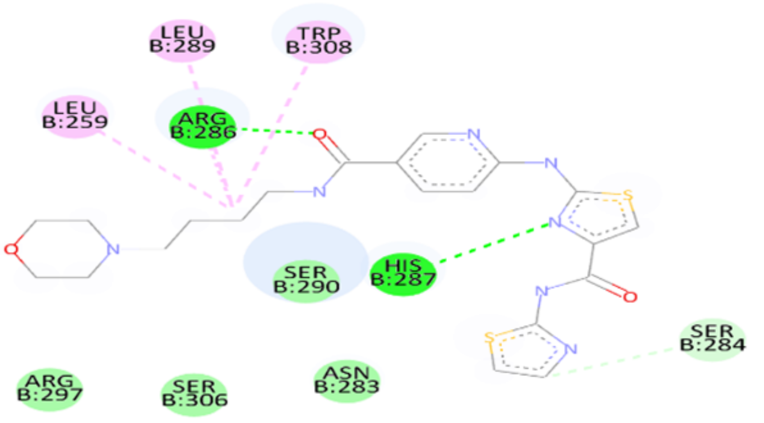	**-6.4**
**15**	**C15**	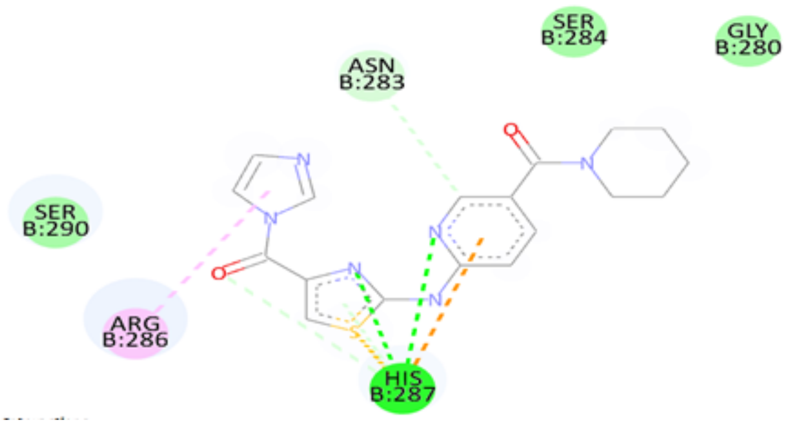	**-5.6**

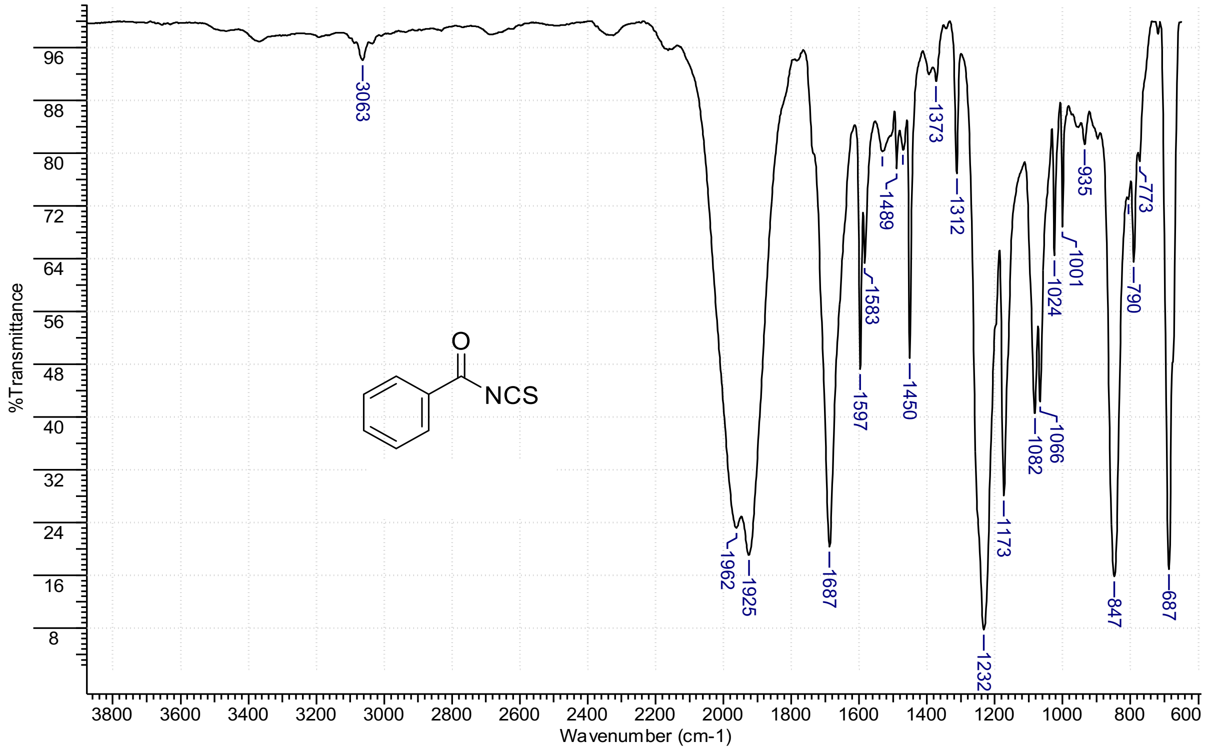

Figure S1. FT-IR spectrum of intermediate 3 (Benzoyl isothiocyanate)

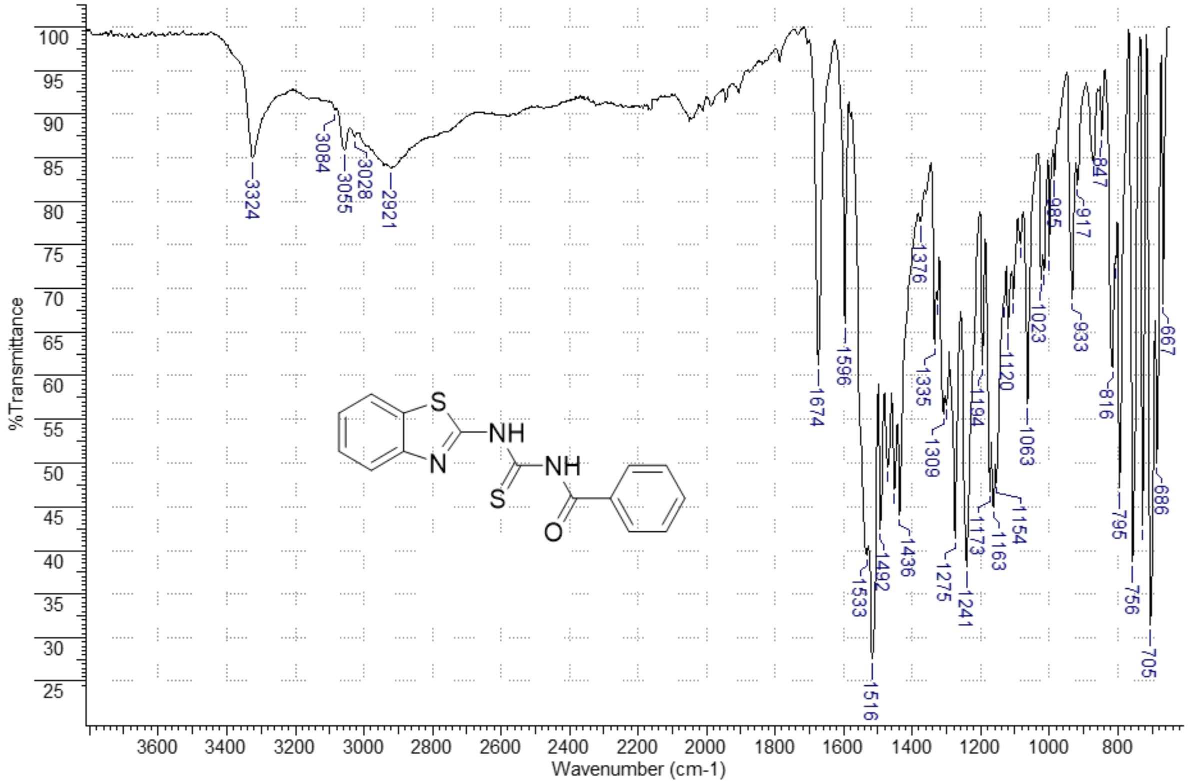

Figure S2. FT-IR spectrum of intermediate 4a

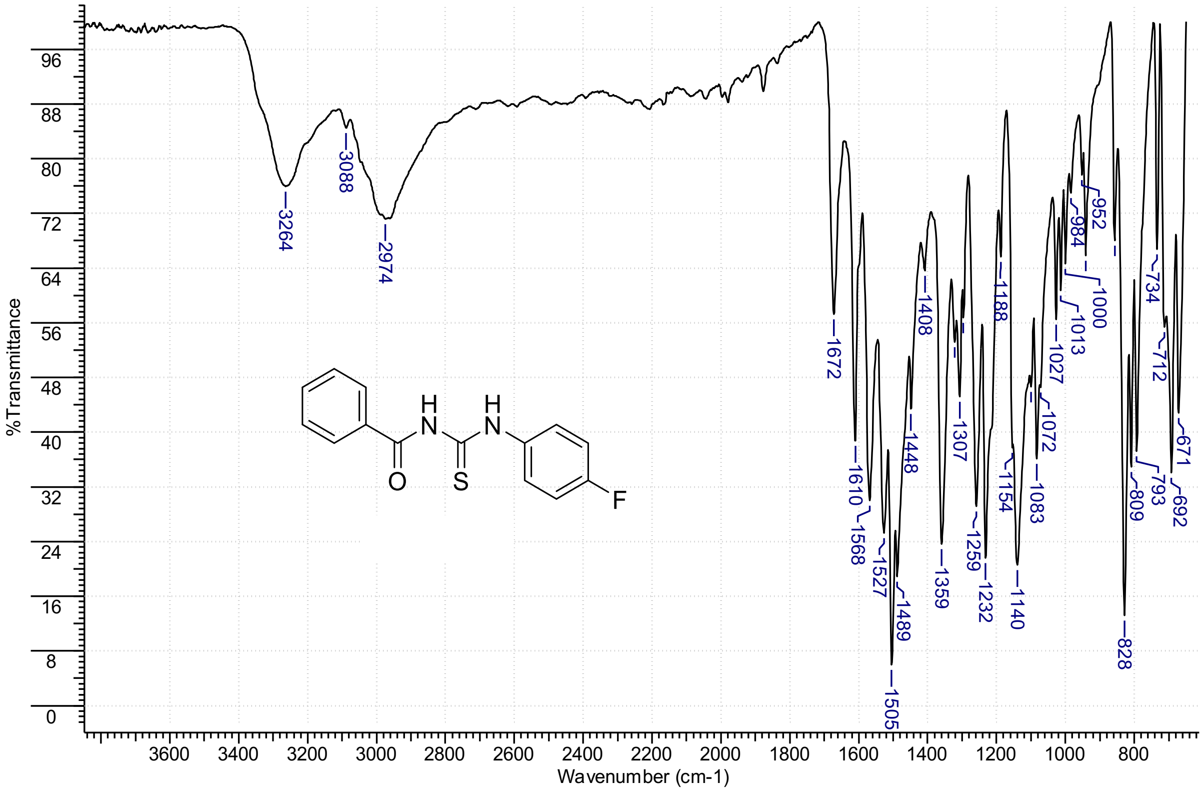

Figure S3. FT-IR spectrum of intermediate 4b

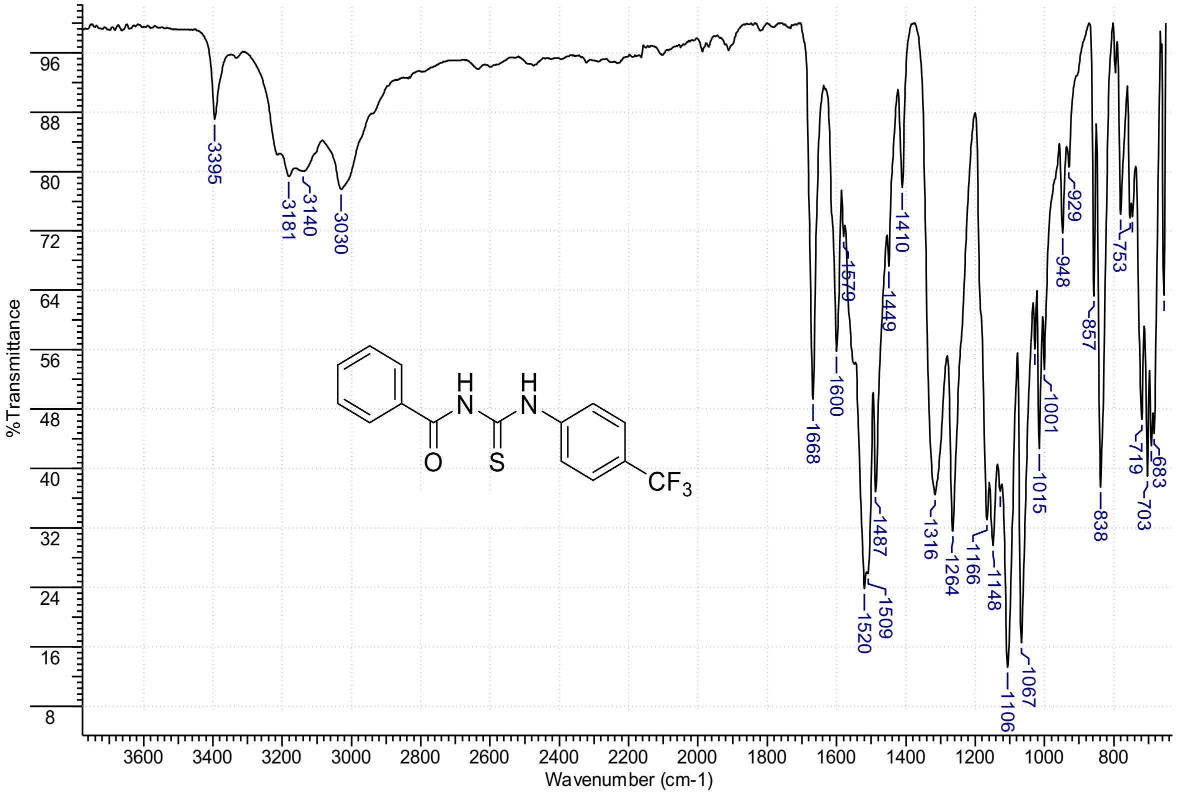

Figure S4. FT-IR spectrum of intermediate 4c

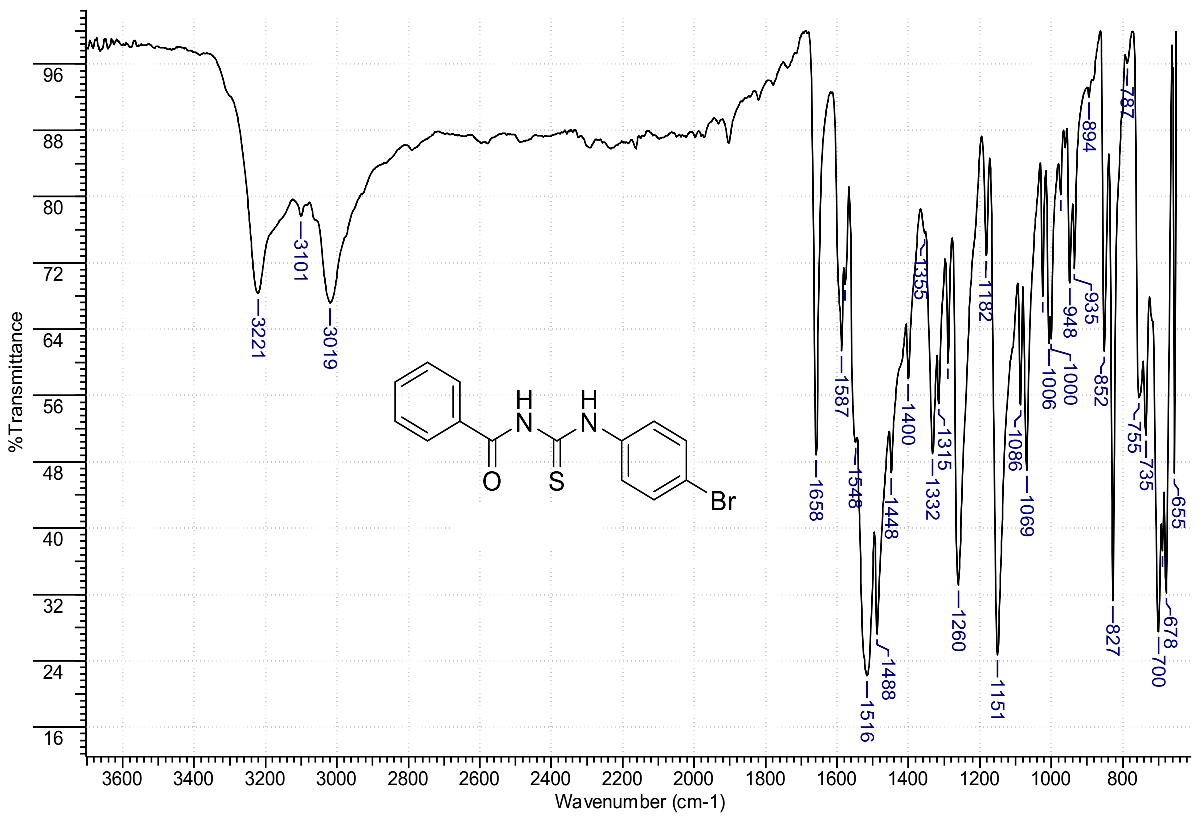

Figure S5. FT-IR spectrum of intermediate 4d

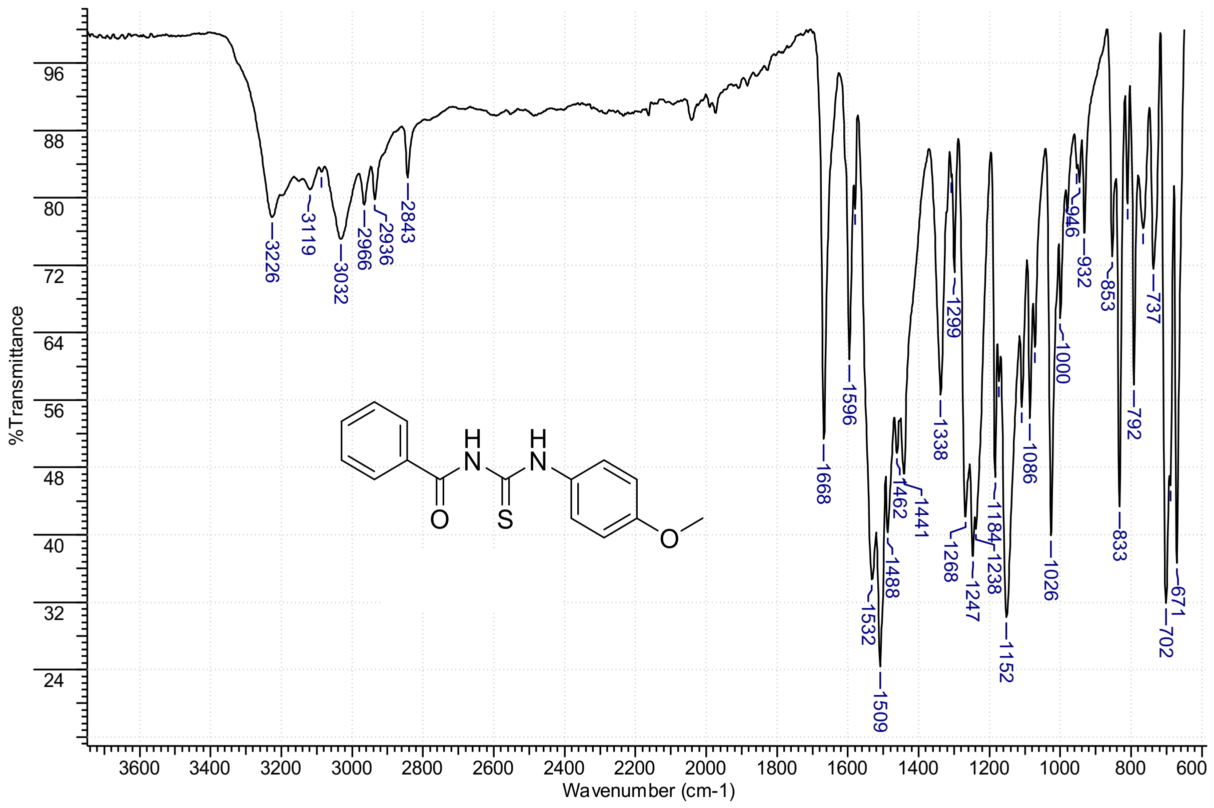

Figure S6. FT-IR spectrum of intermediate 4e

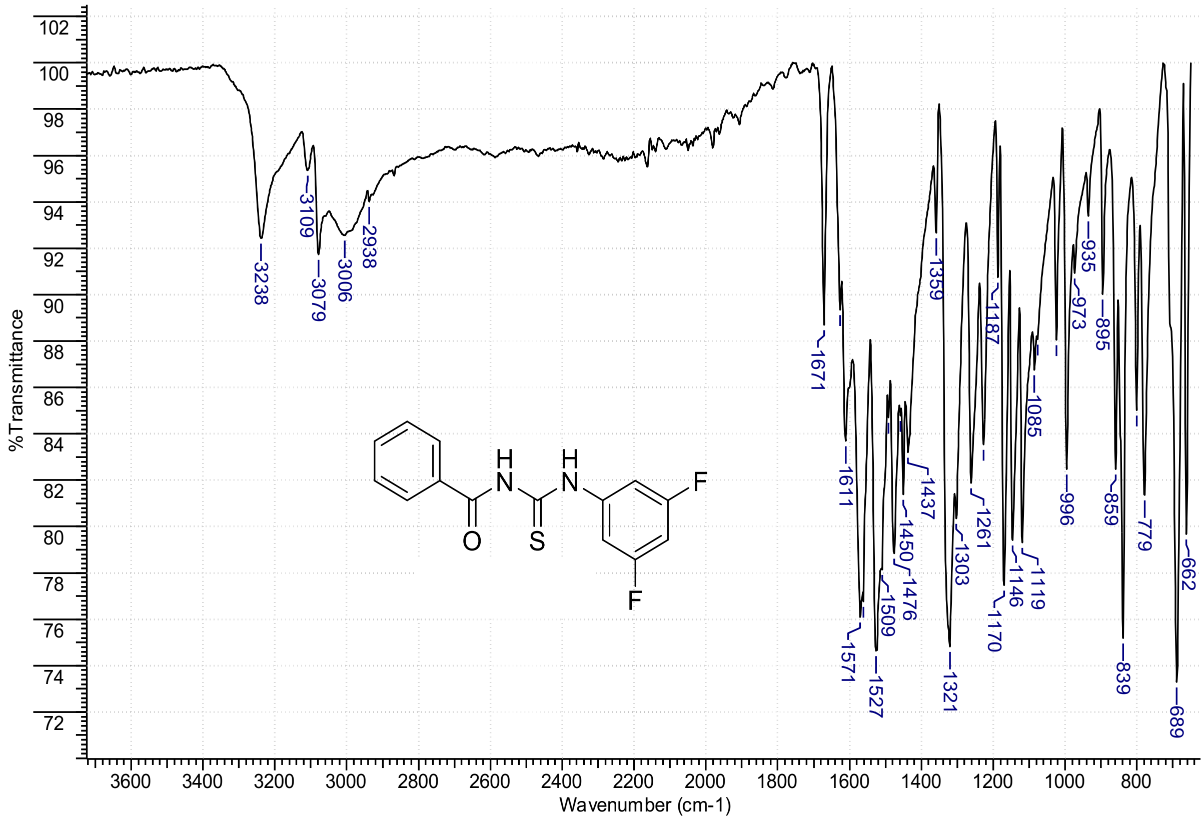

Figure S7. FT-IR spectrum of intermediate 4f

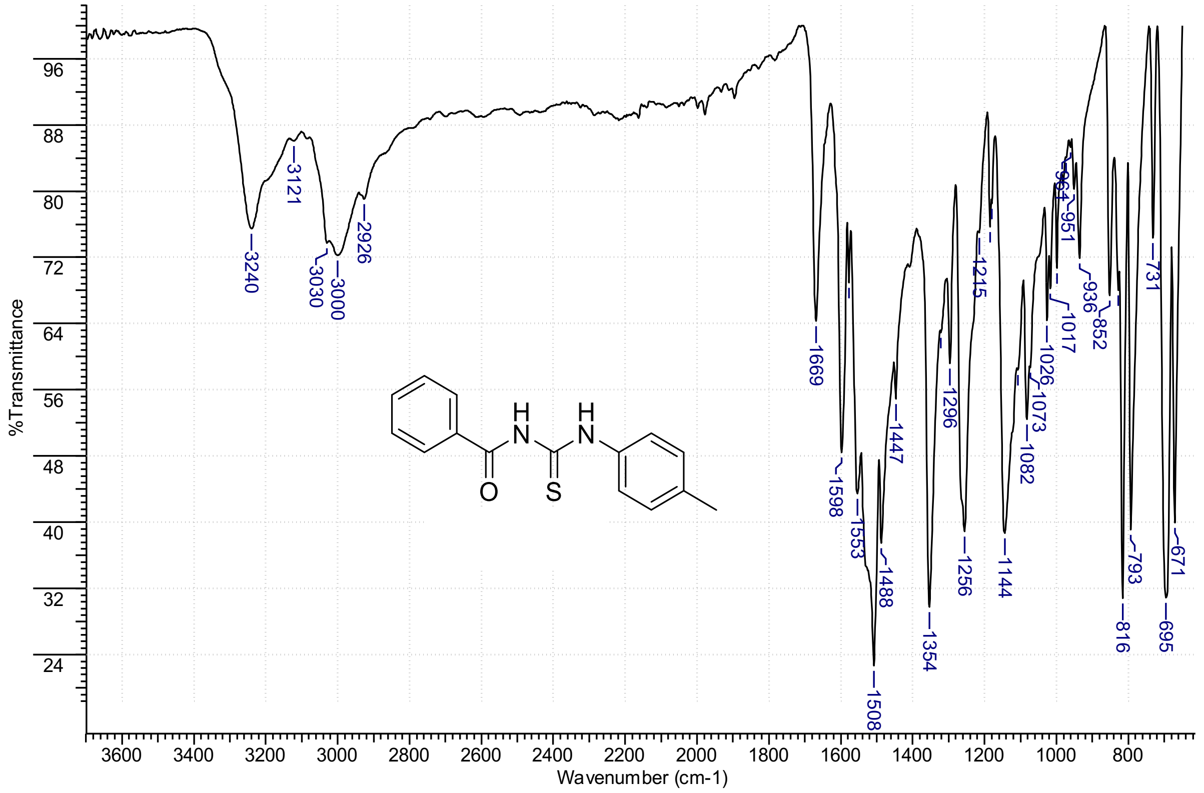

Figure S8. FT-IR spectrum of intermediate 4g

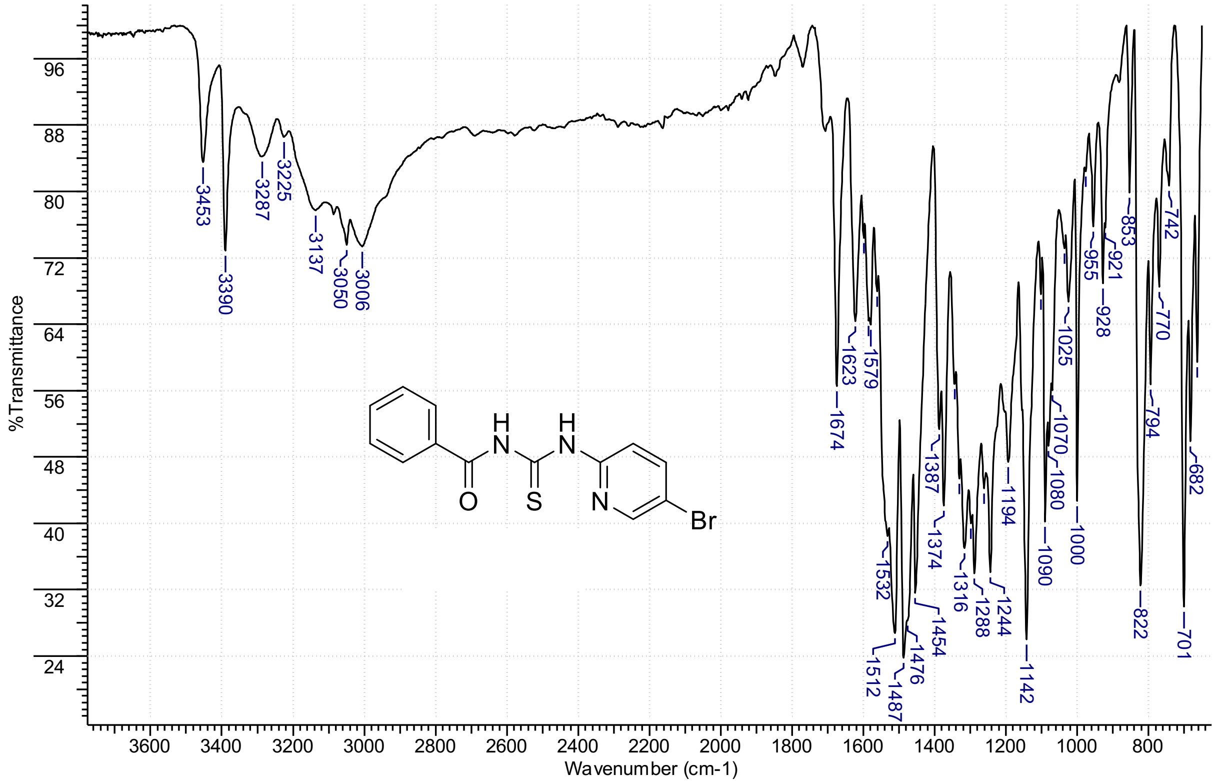

Figure S9. FT-IR spectrum of intermediate 4h

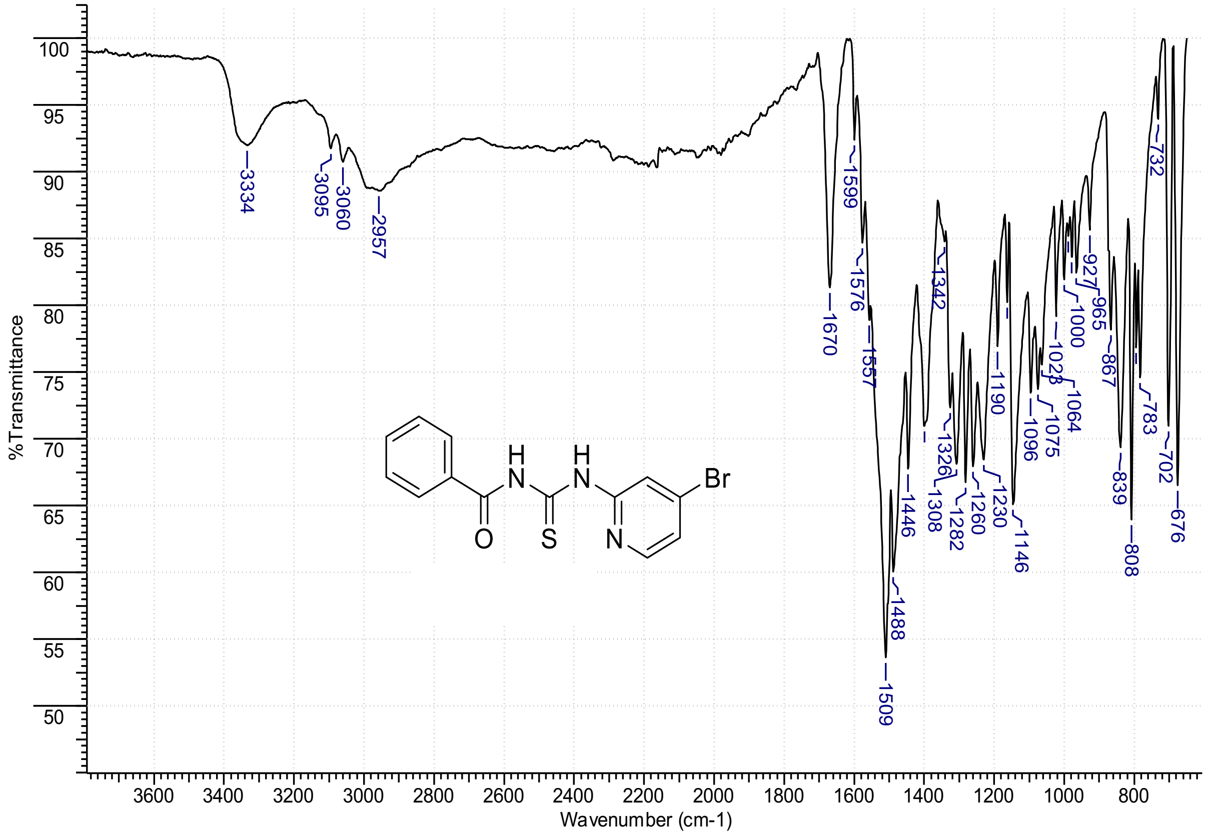

Figure S10. FT-IR spectrum of intermediate 4i

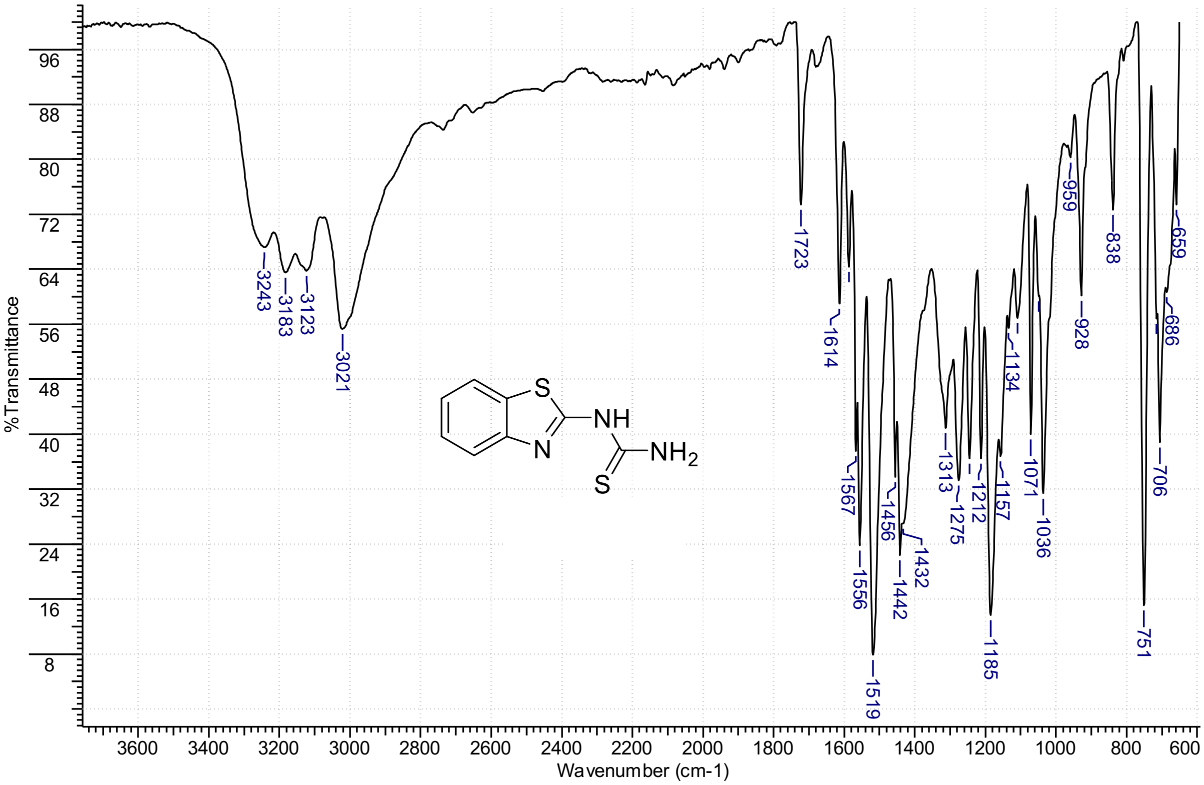

Figure S11. FT-IR spectrum of intermediate 5a

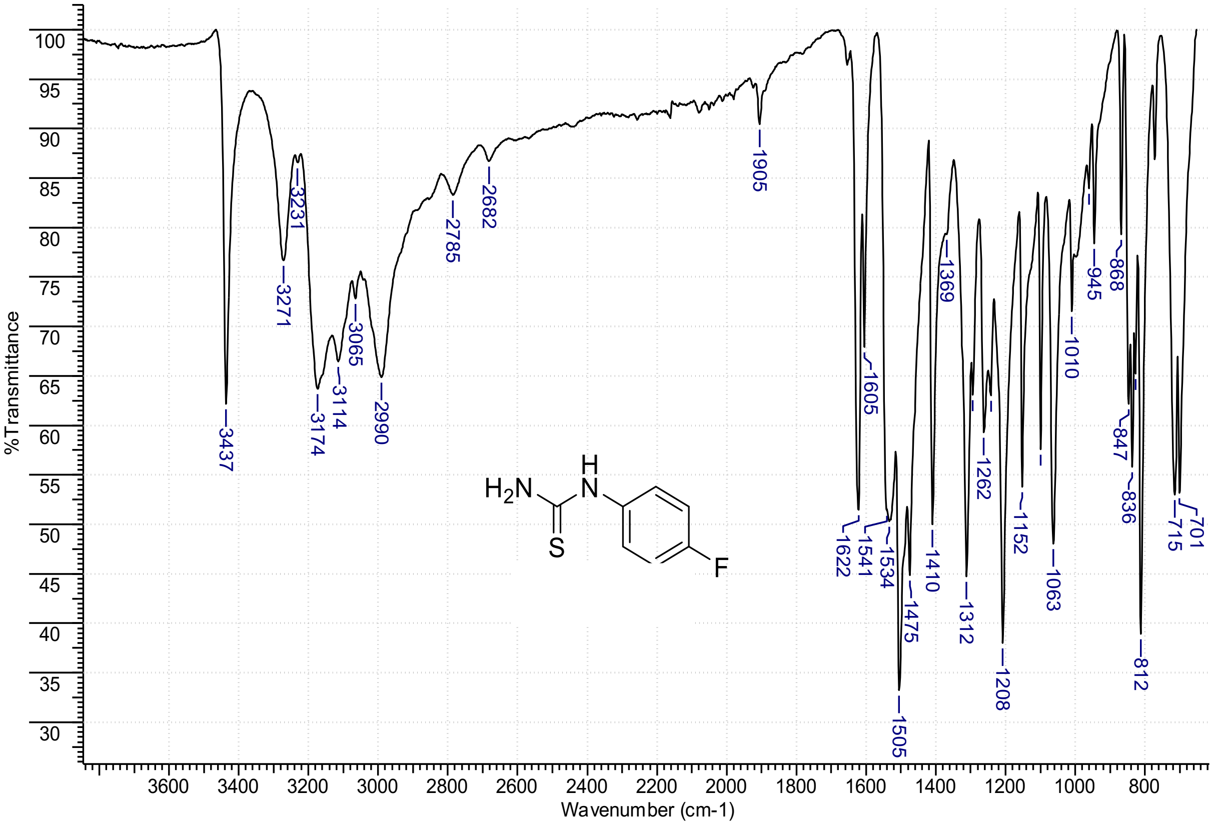

Figure S12. FT-IR spectrum of intermediate 5b

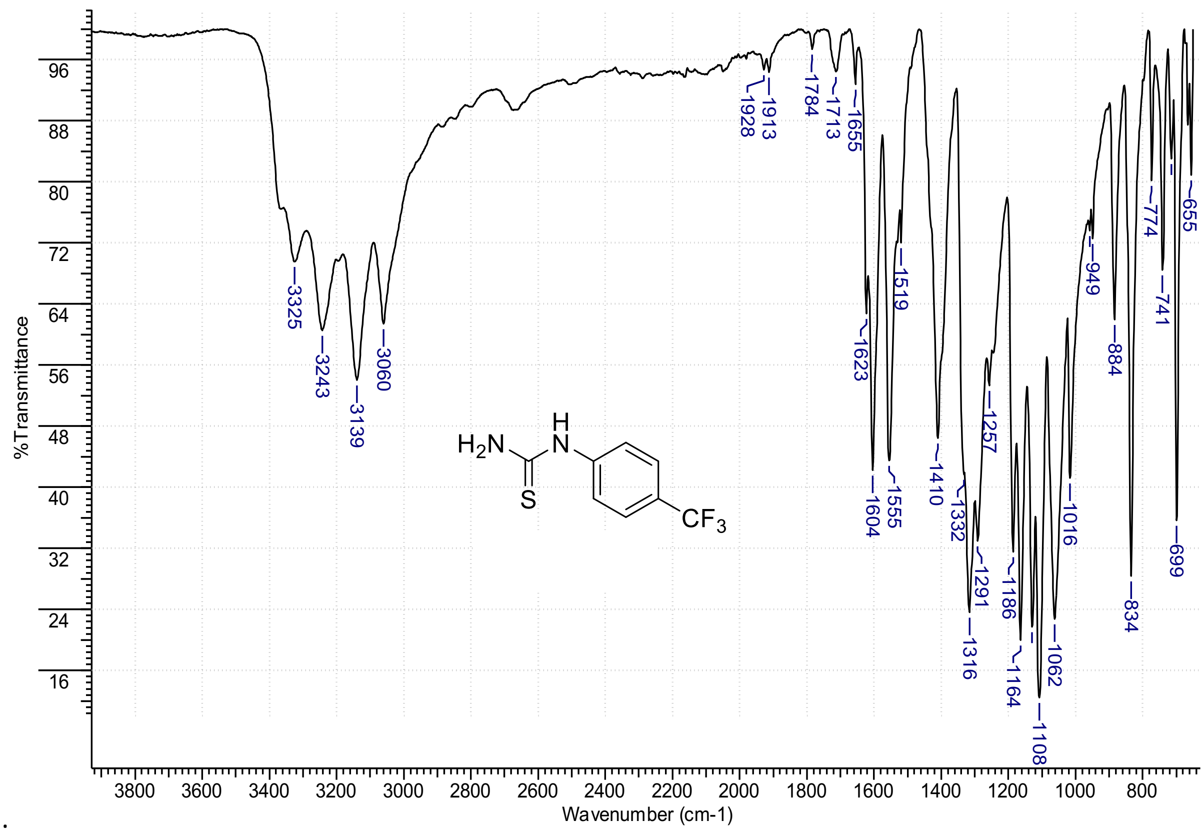

Figure S13. FT-IR spectrum of intermediate 5c

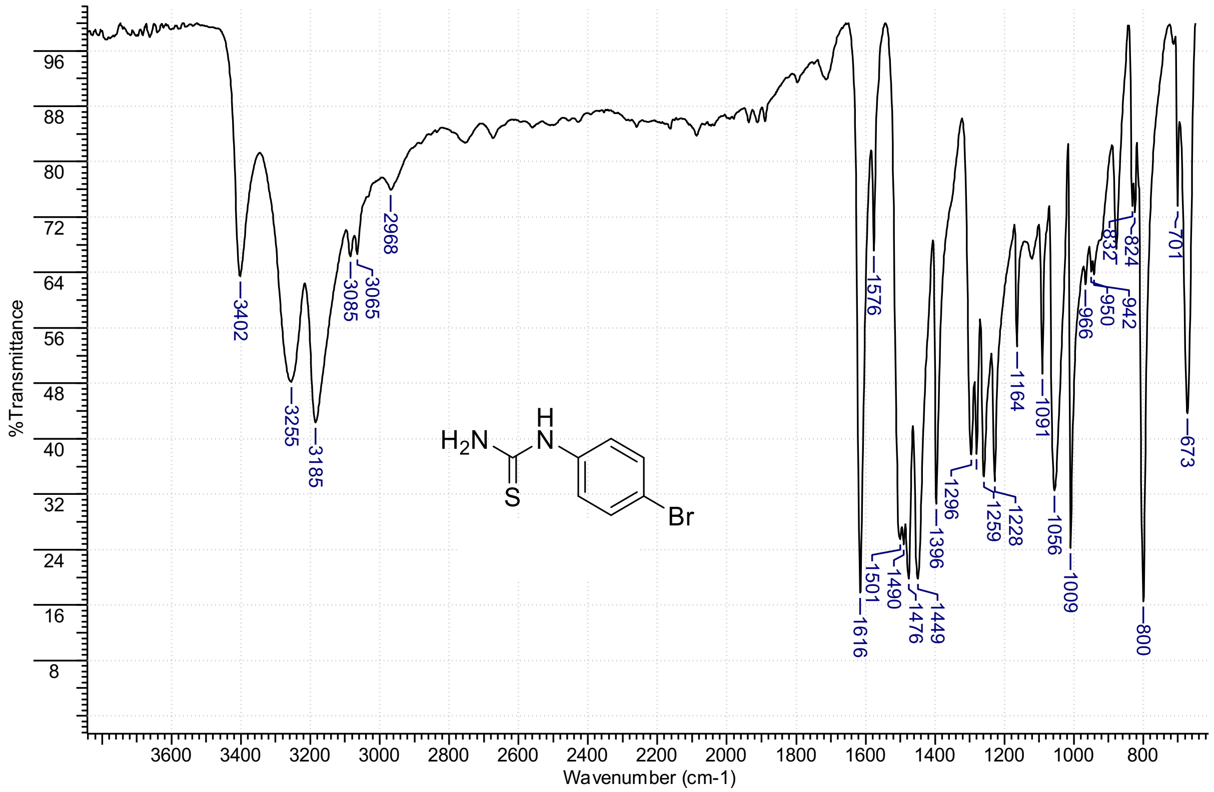

Figure S14. FT-IR spectrum of intermediate 5d

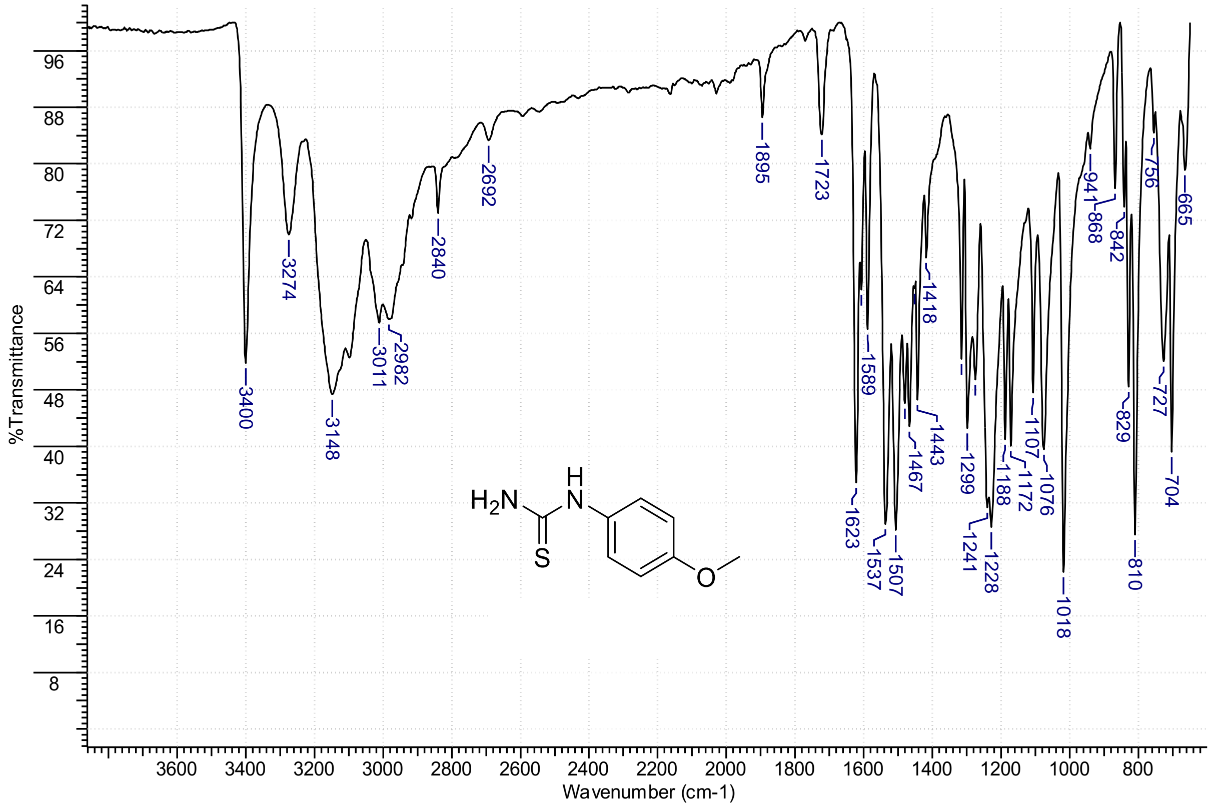

Figure S15. FT-IR spectrum of intermediate 5e

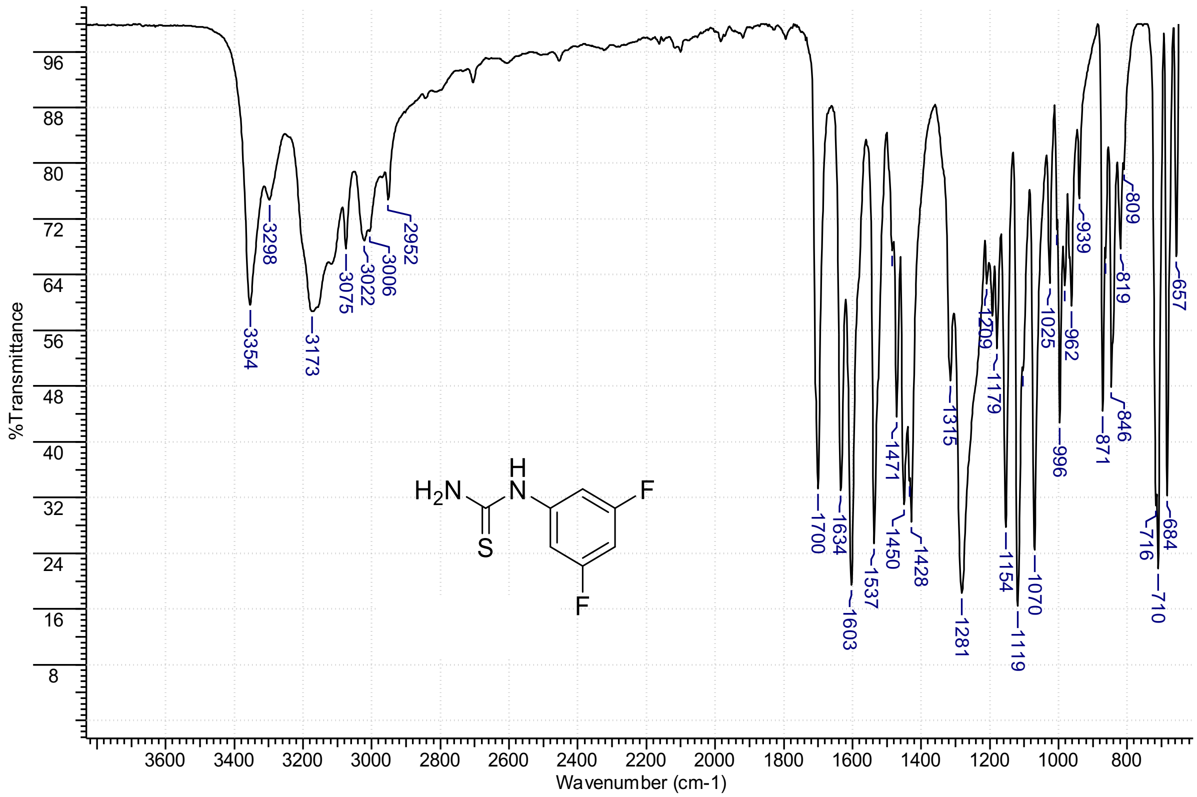

Figure S16. FT-IR spectrum of intermediate 5f

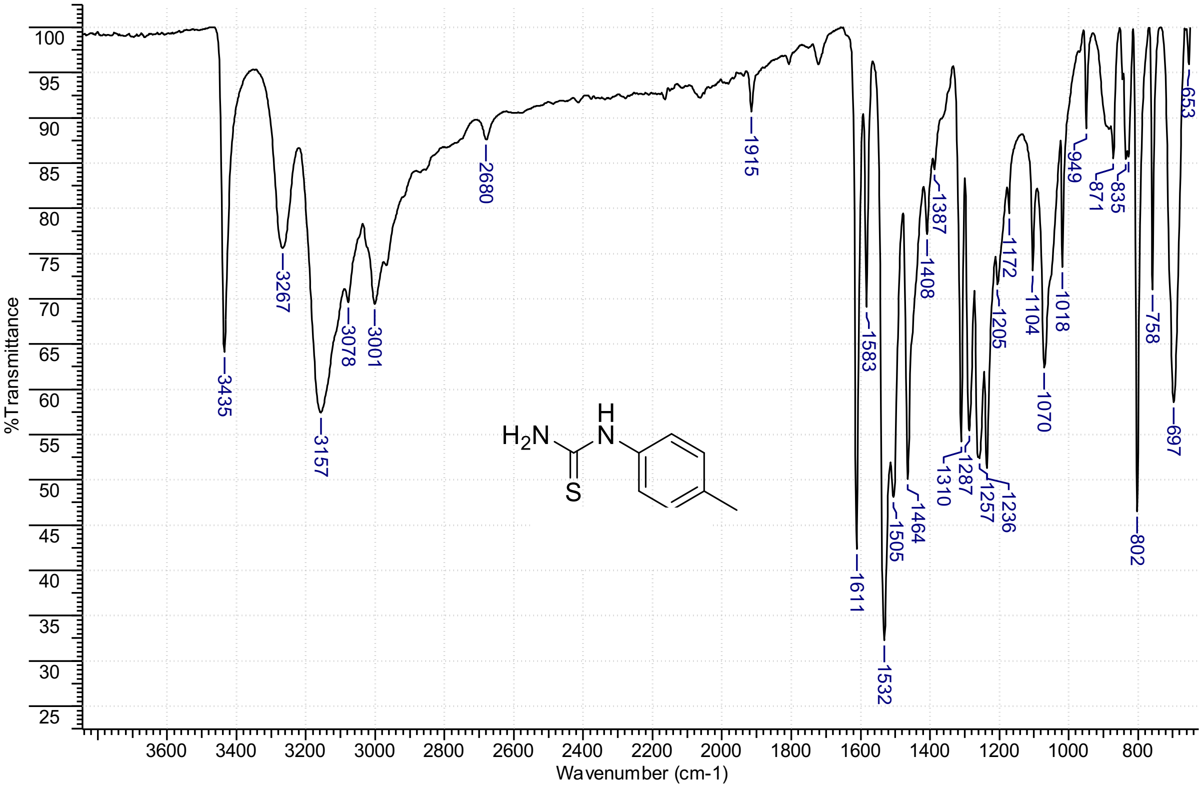

Figure S17. FT-IR spectrum of intermediate 5g

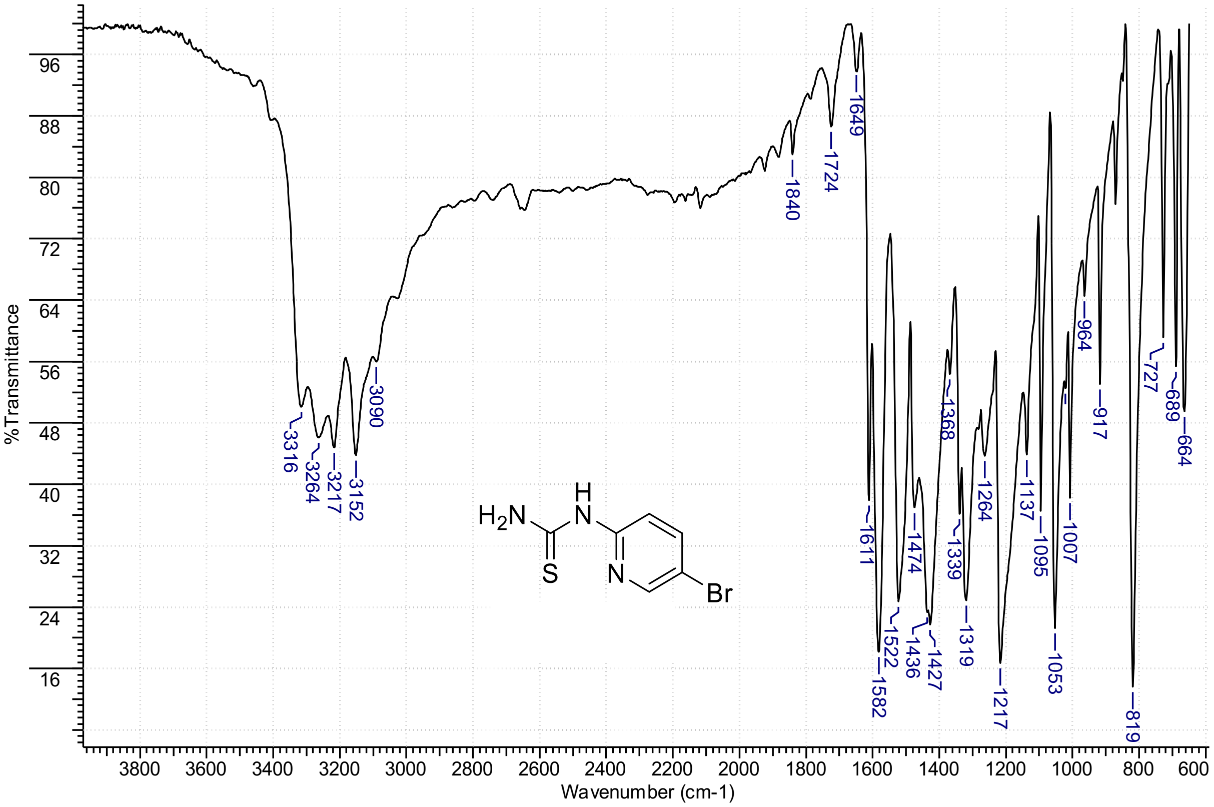

Figure S18. FT-IR spectrum of intermediate 5h

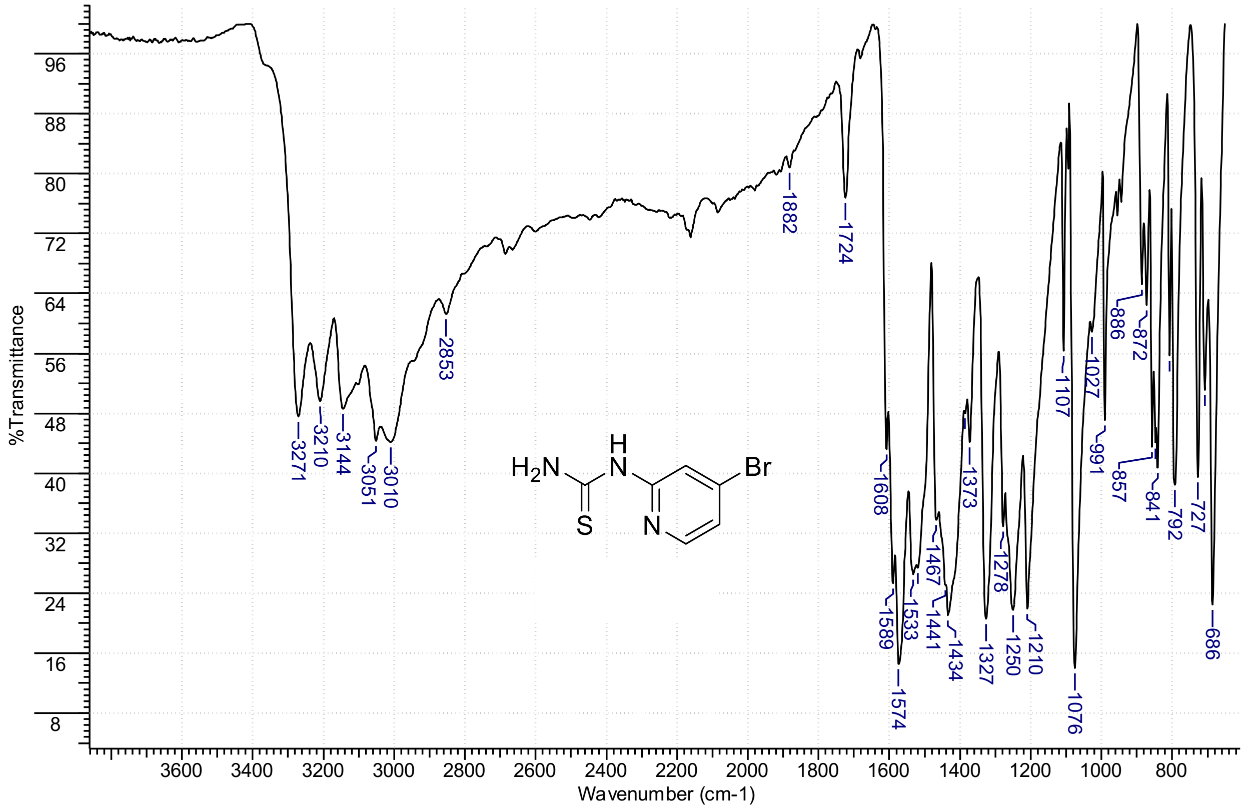

Figure S19. FT-IR spectrum of intermediate 5i

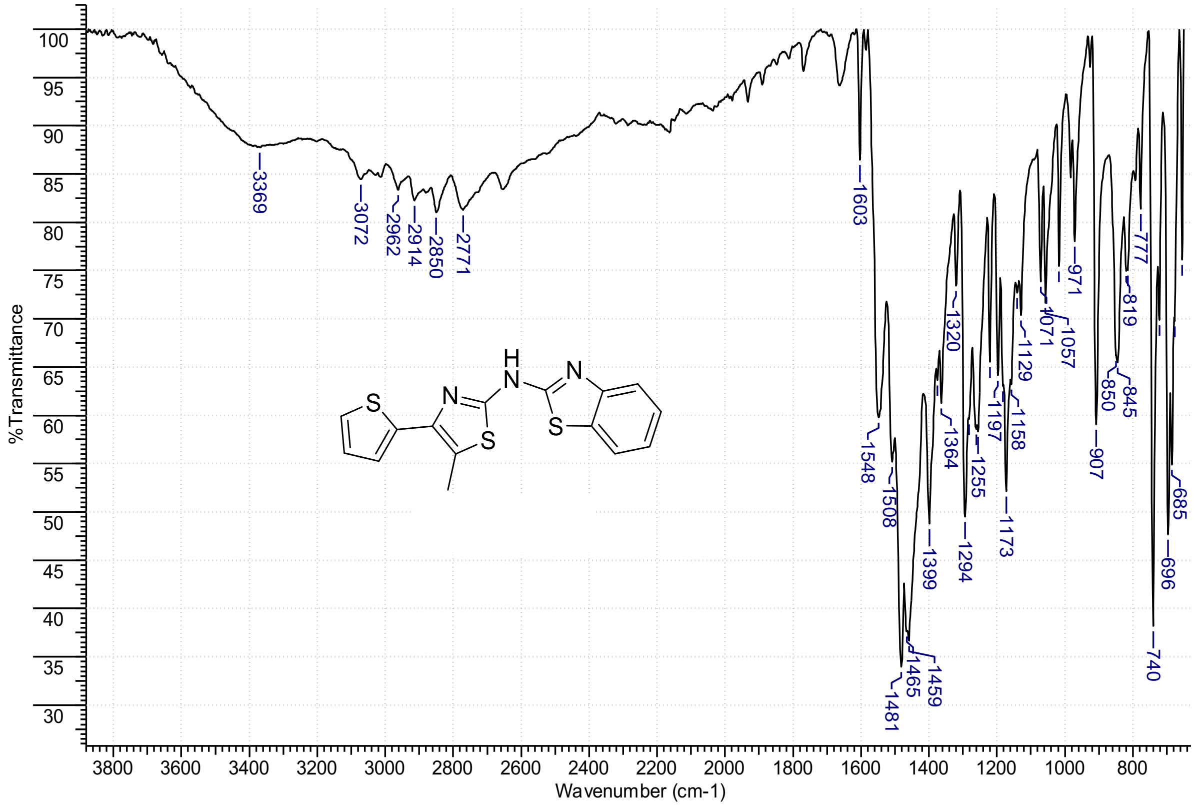

Figure S20. FT-IR spectrum of compound **C1**

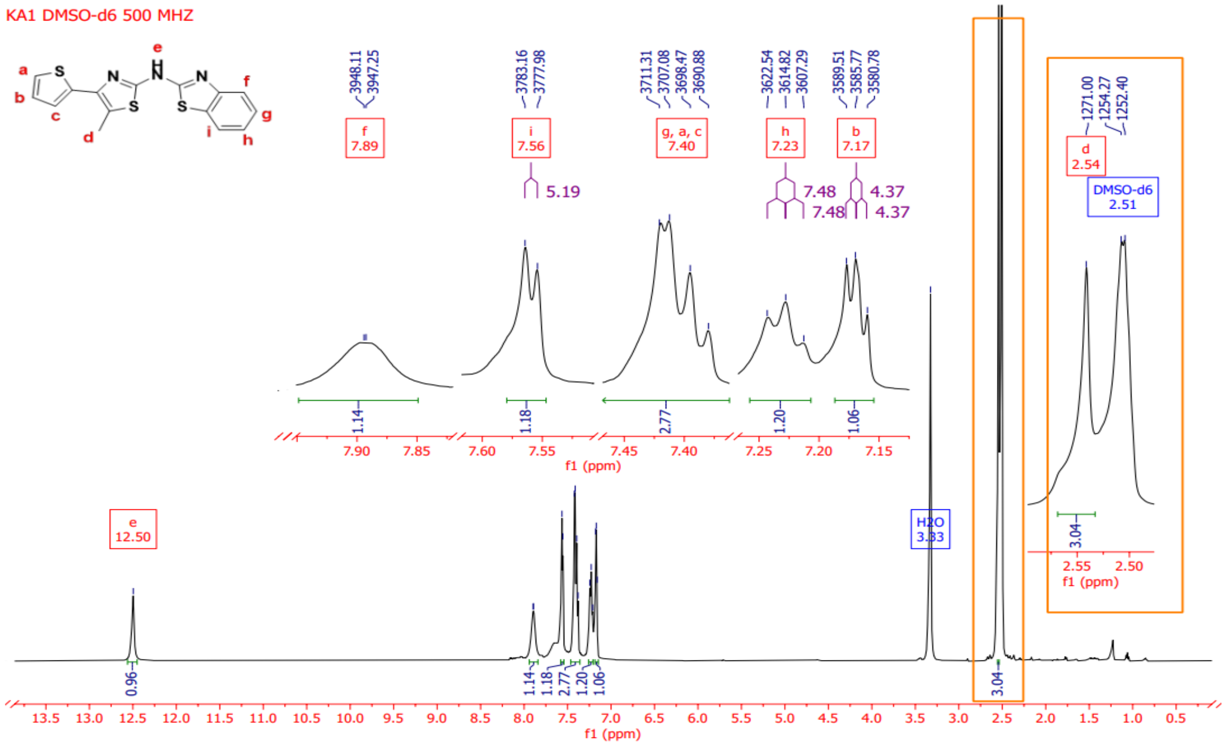

Figure S21. ^{1}H NMRspectrum of compound **C1** (500 MHz, DMSO-d$_6$)

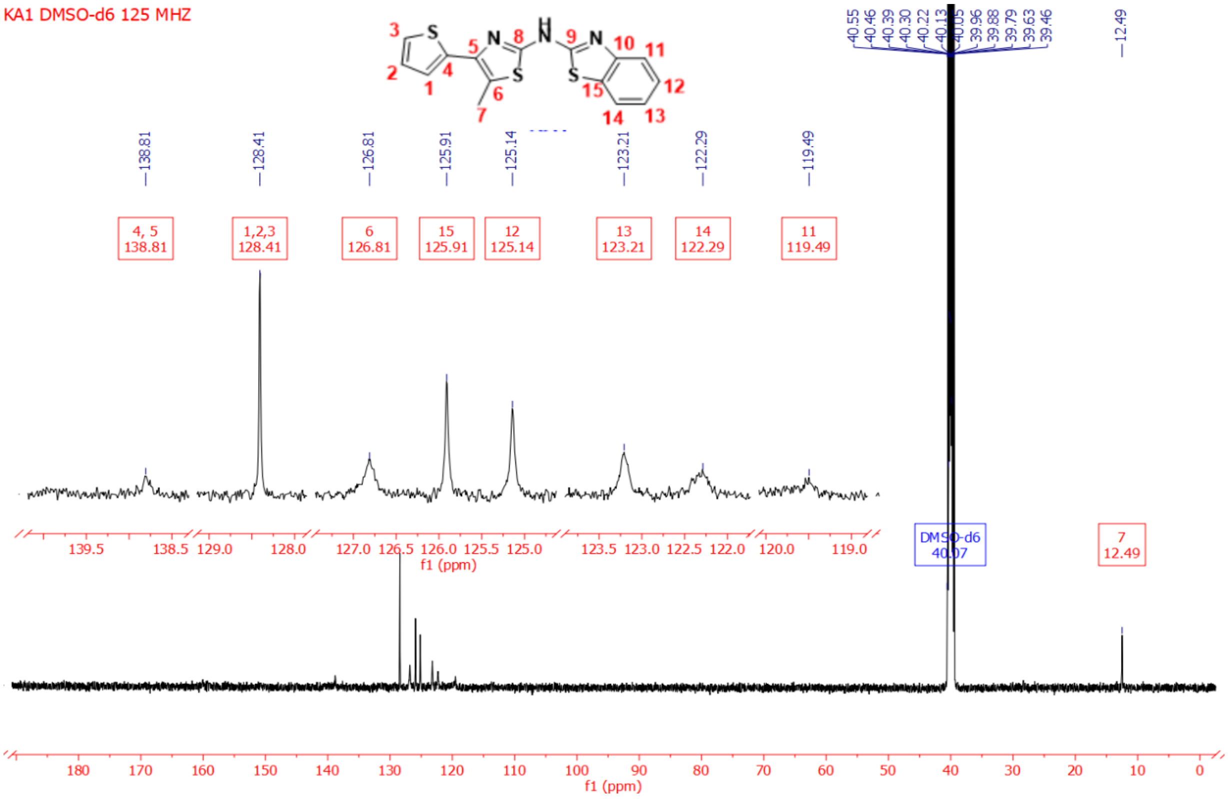

Figure S22. ^{13}C NMRspectrum of compound **C1** (125 MHz, DMSO-d_6)

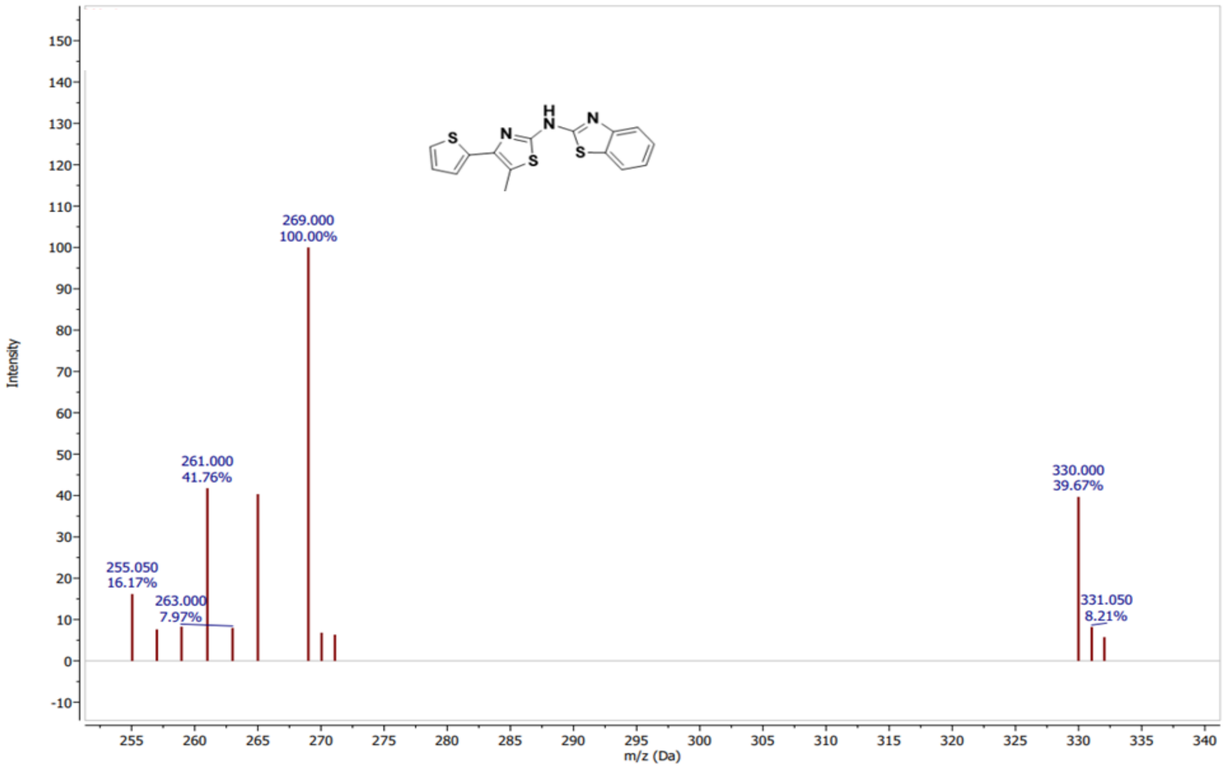

Figure S23. LC-MS/MS spectrum of compound **C1**

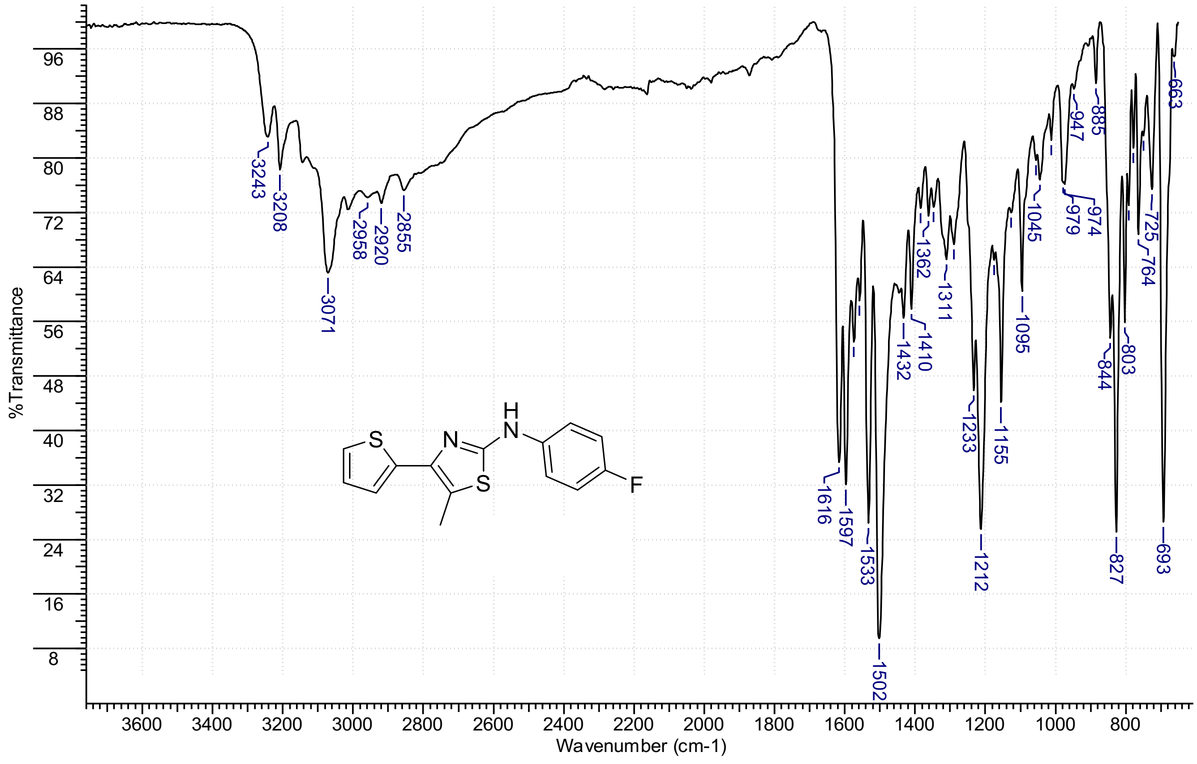

Figure S24. FT-IR spectrum of compound **C2**

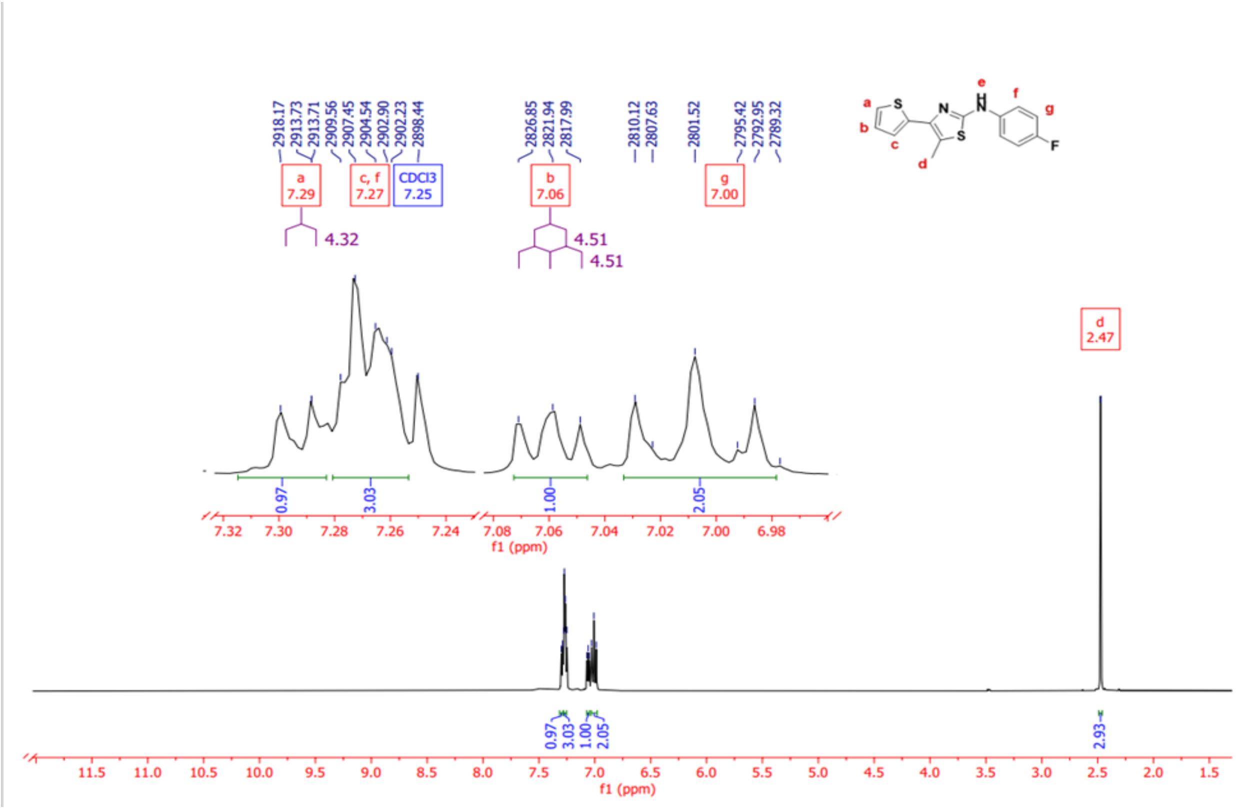

Figure S25. ^{1}H NMRspectrum of compound **C2** (400 MHz, $CDCl_3$)

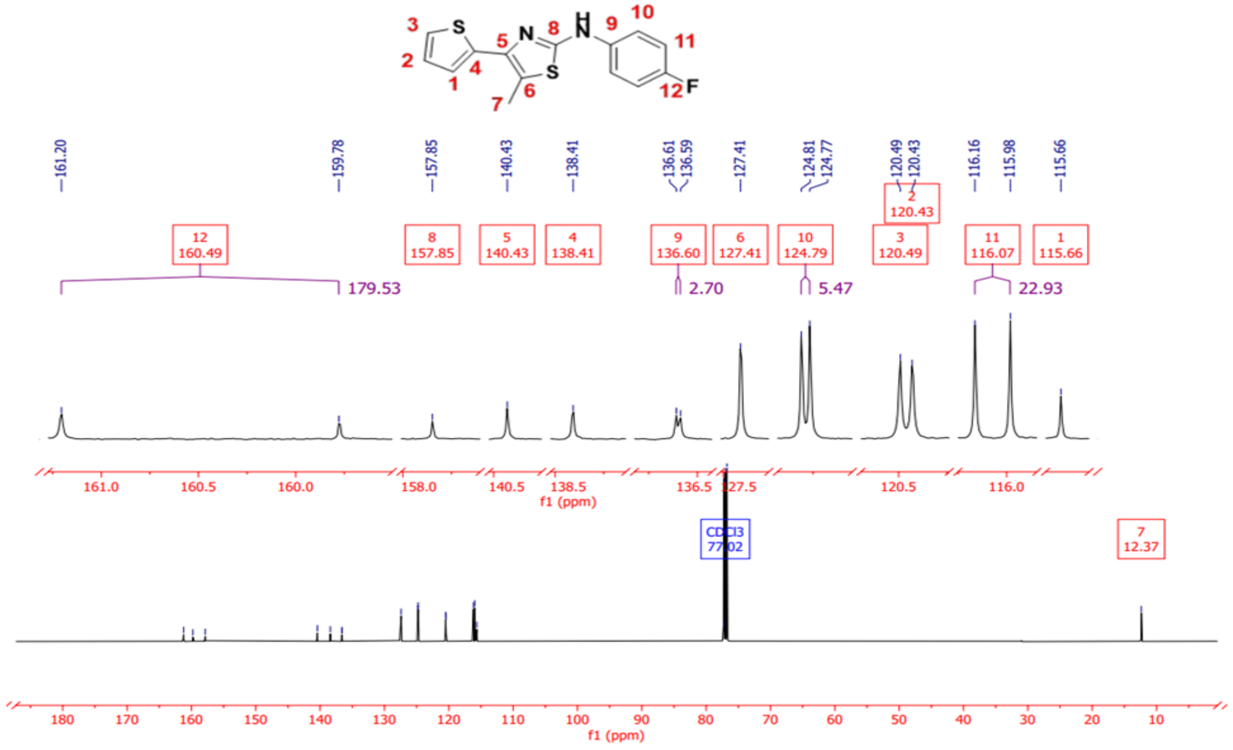

Figure S26. ^{13}C NMRspectrum of compound **C2** (125 MHz, $CDCl_3$)

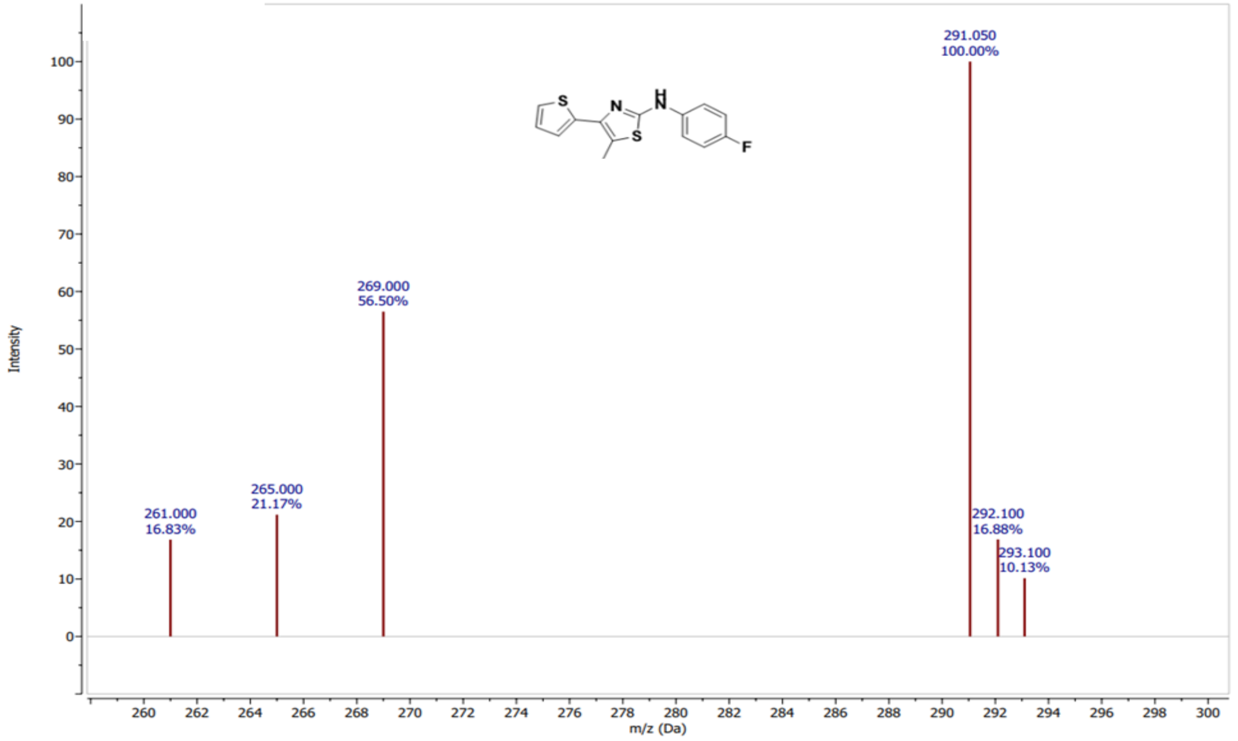

Figure S27. LC-MS/MS spectrum of compound **C2**

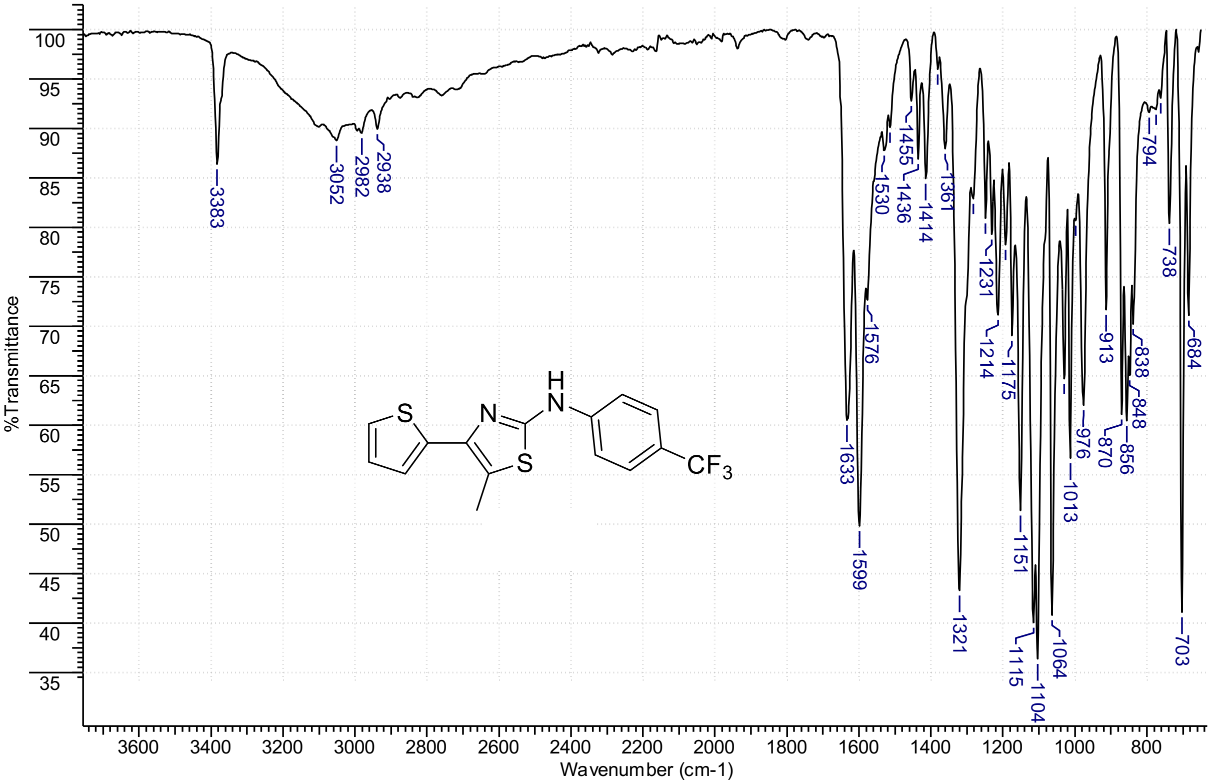

Figure S28. FT-IR spectrum of compound **C3**

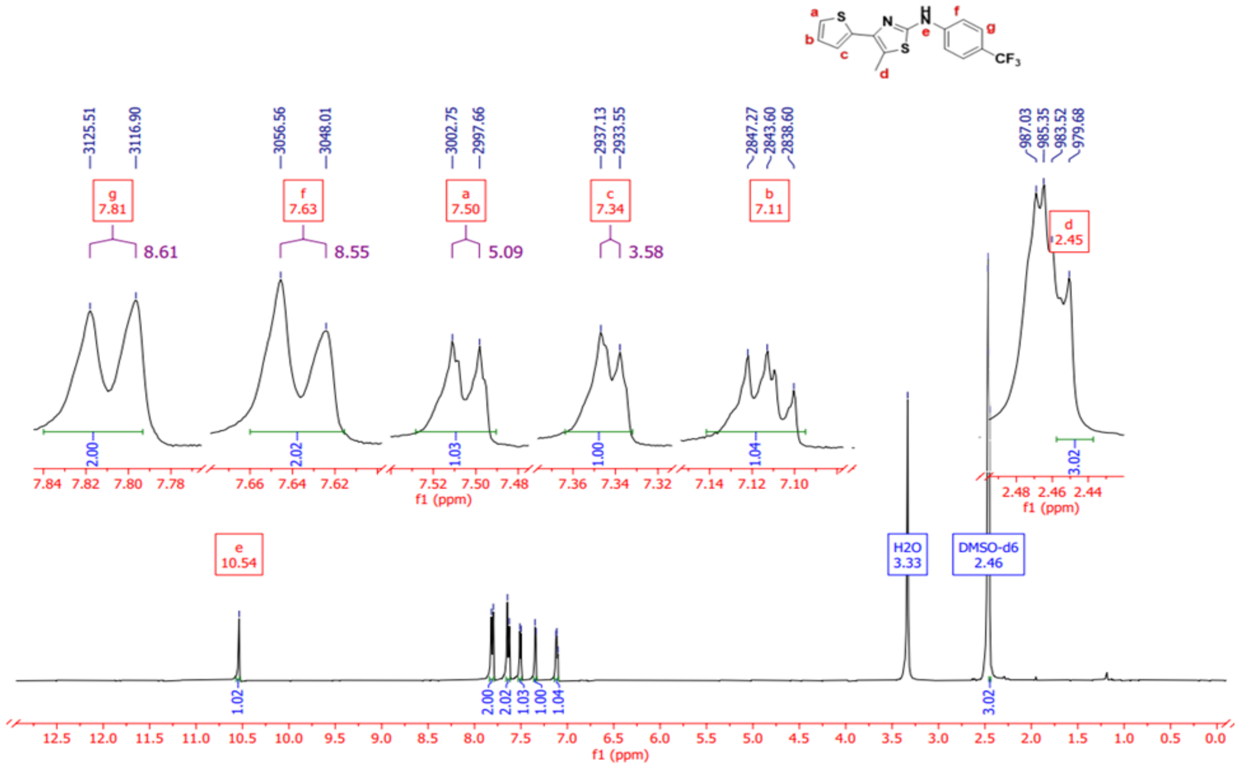

Figure S29. ^{1}H NMRspectrum of compound **C3** (400 MHz, DMSO-d_6)

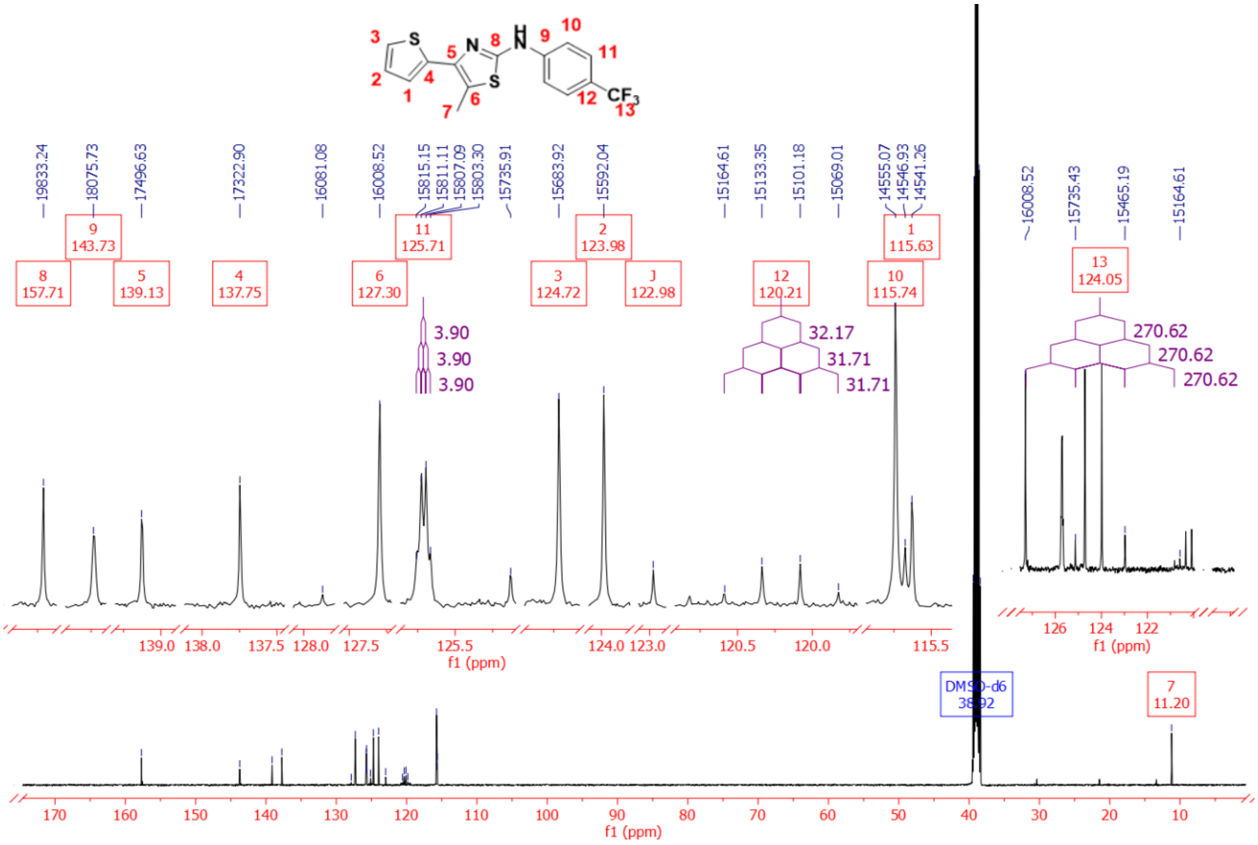

Figure S30. ^{13}C NMRspectrum of compound **C3** (125 MHz, DMSO-d_6)

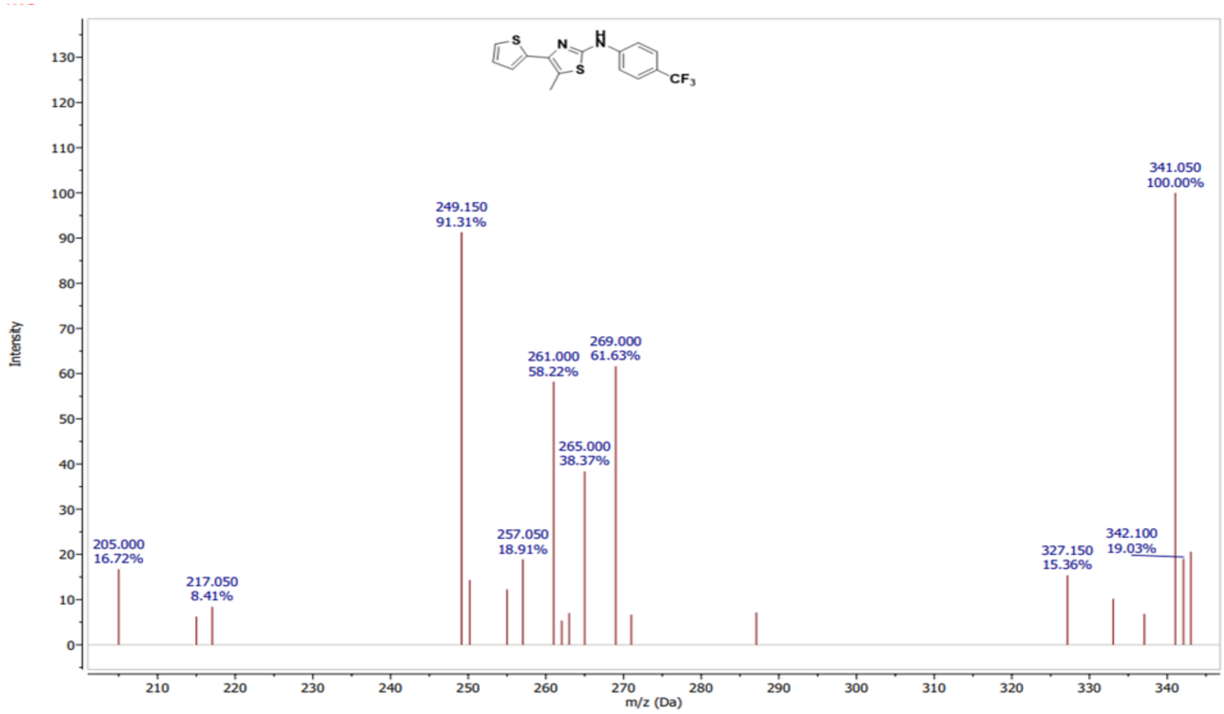

Figure S31. LC-MS/MS spectrum of compound **C3**

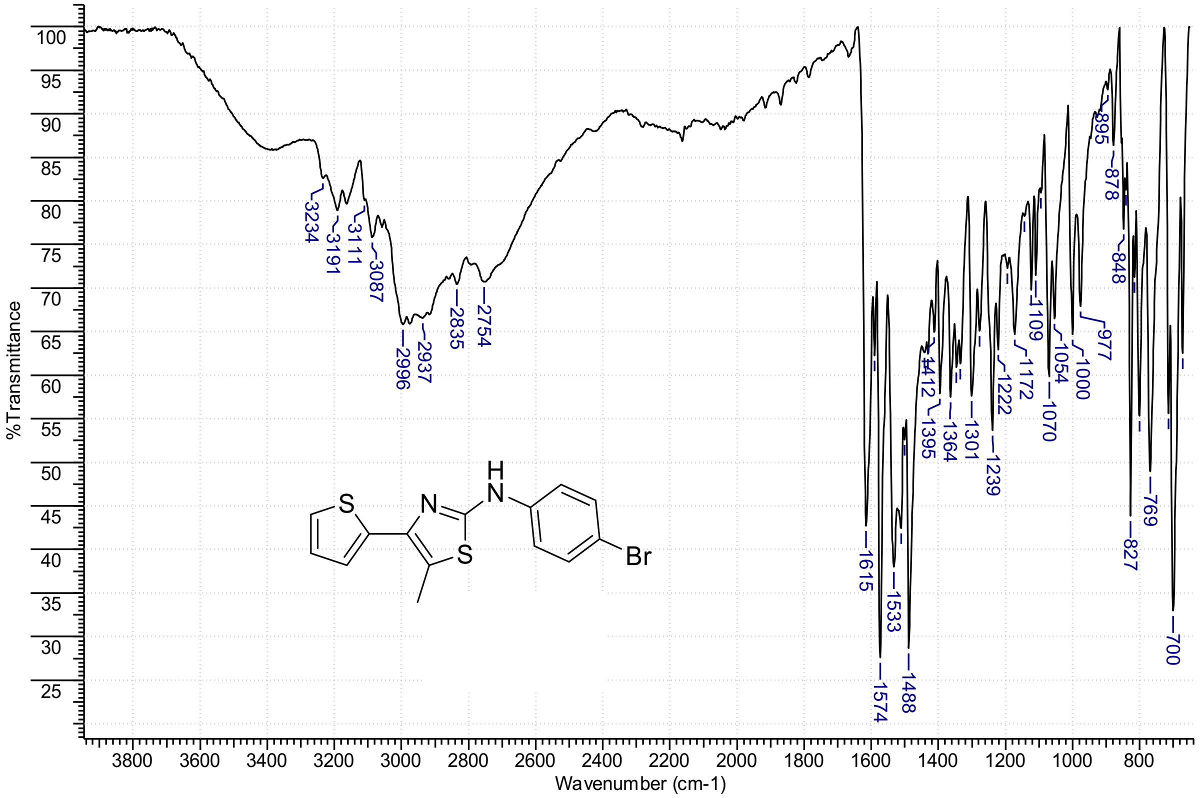

Figure S32. FT-IR spectrum of compound **C4**

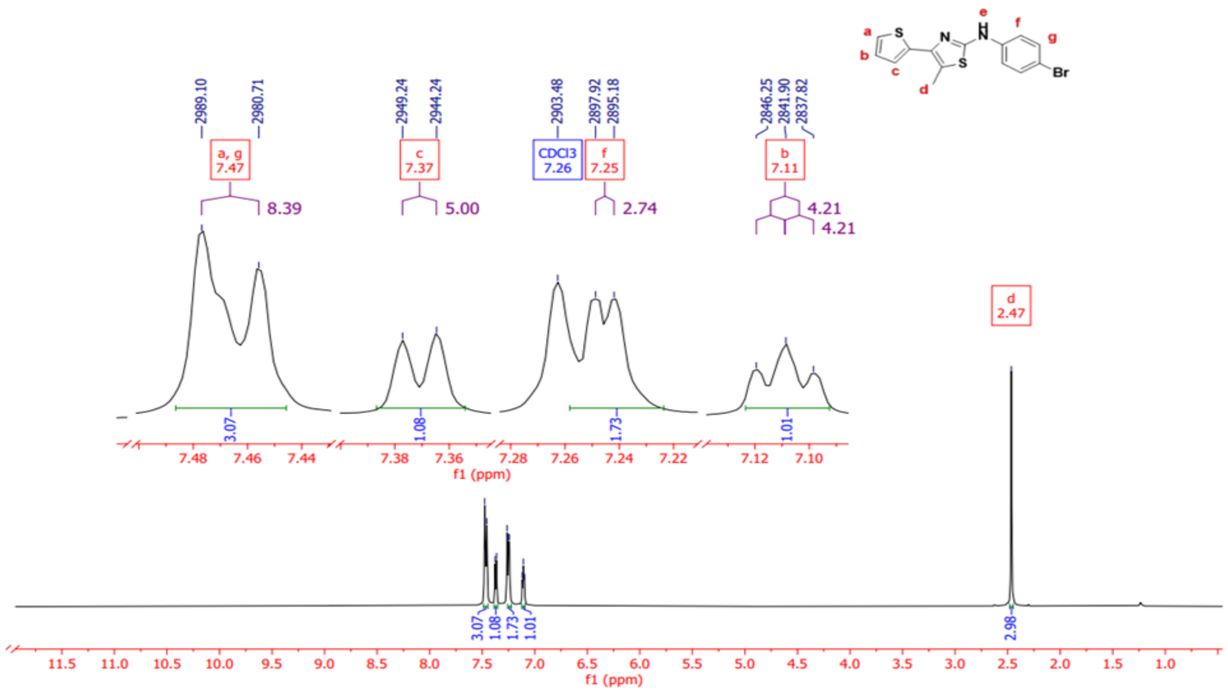

Figure S33. ^{1}H NMRspectrum of compound **C4** (400 MHz, $CDCl_3$)

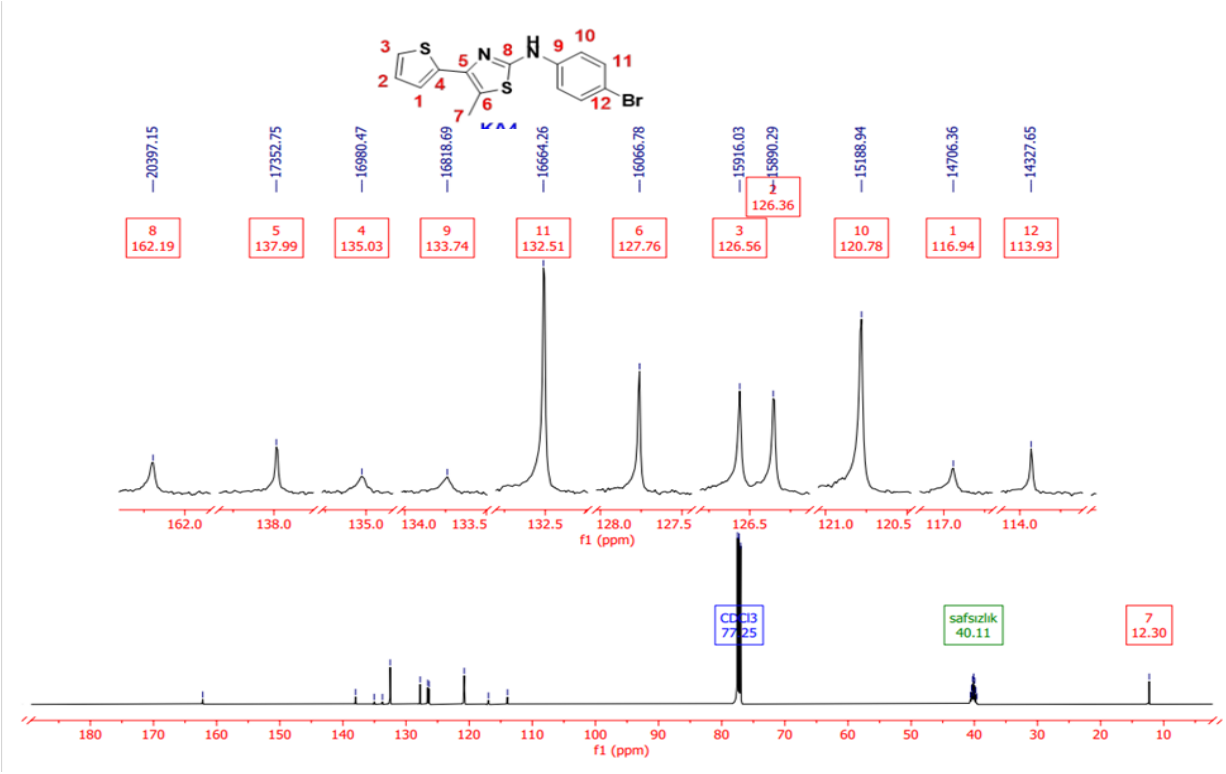

Figure S34. ^{13}C NMRspectrum of compound **C4** (125 MHz, $CDCl_3$)

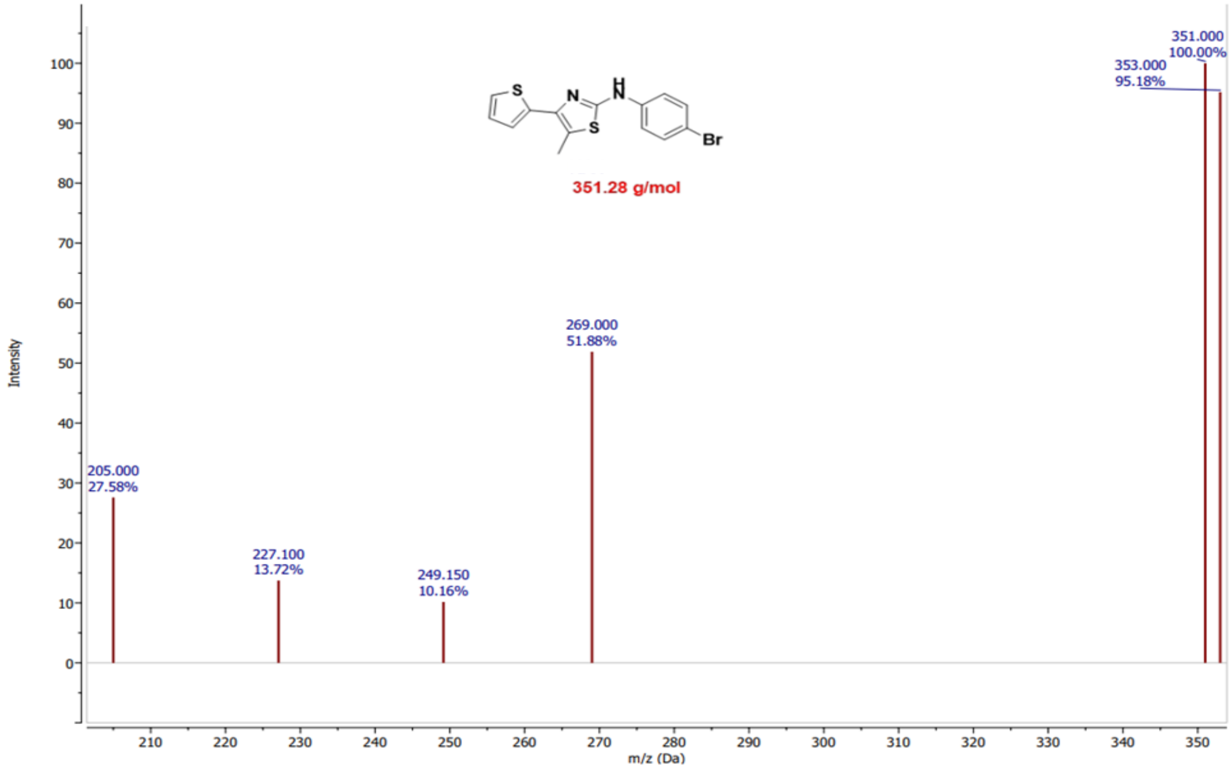

Figure S35. LC-MS/MS spectrum of compound **C4**

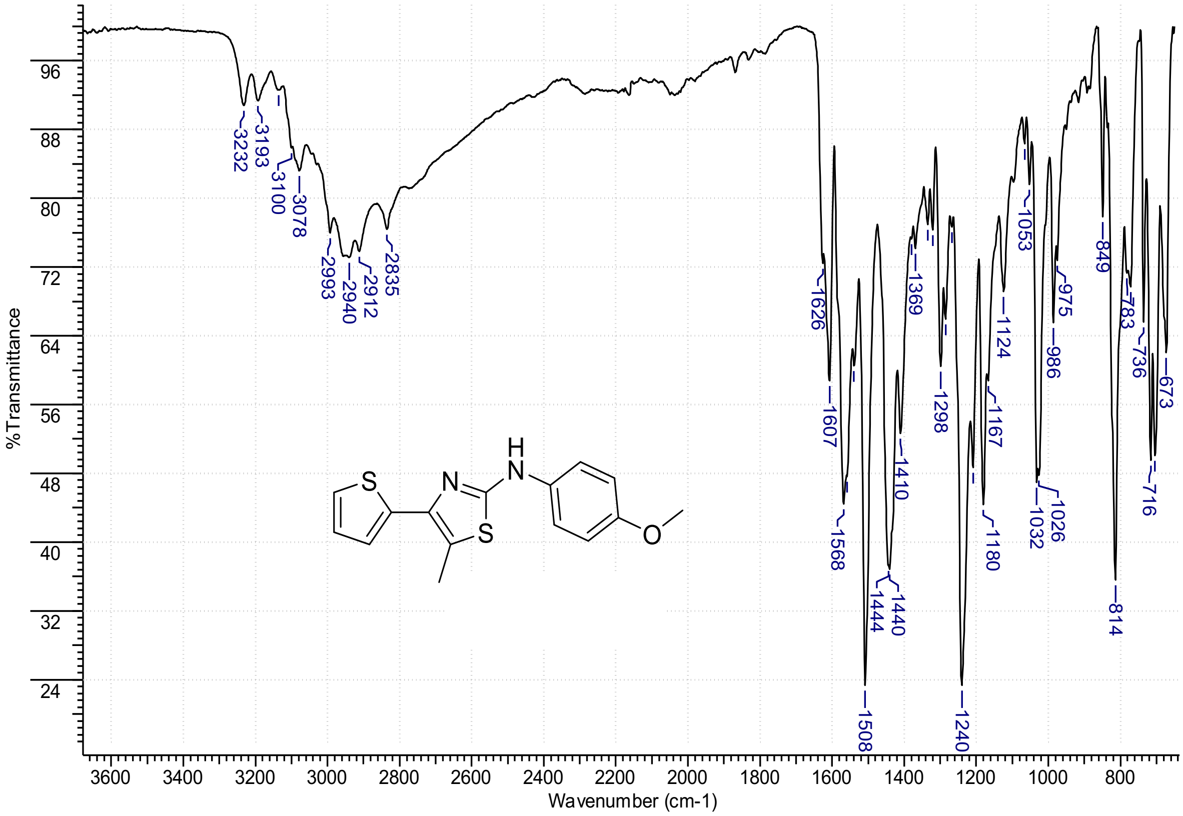

Figure S36. FT-IR spectrum of compound **C5**

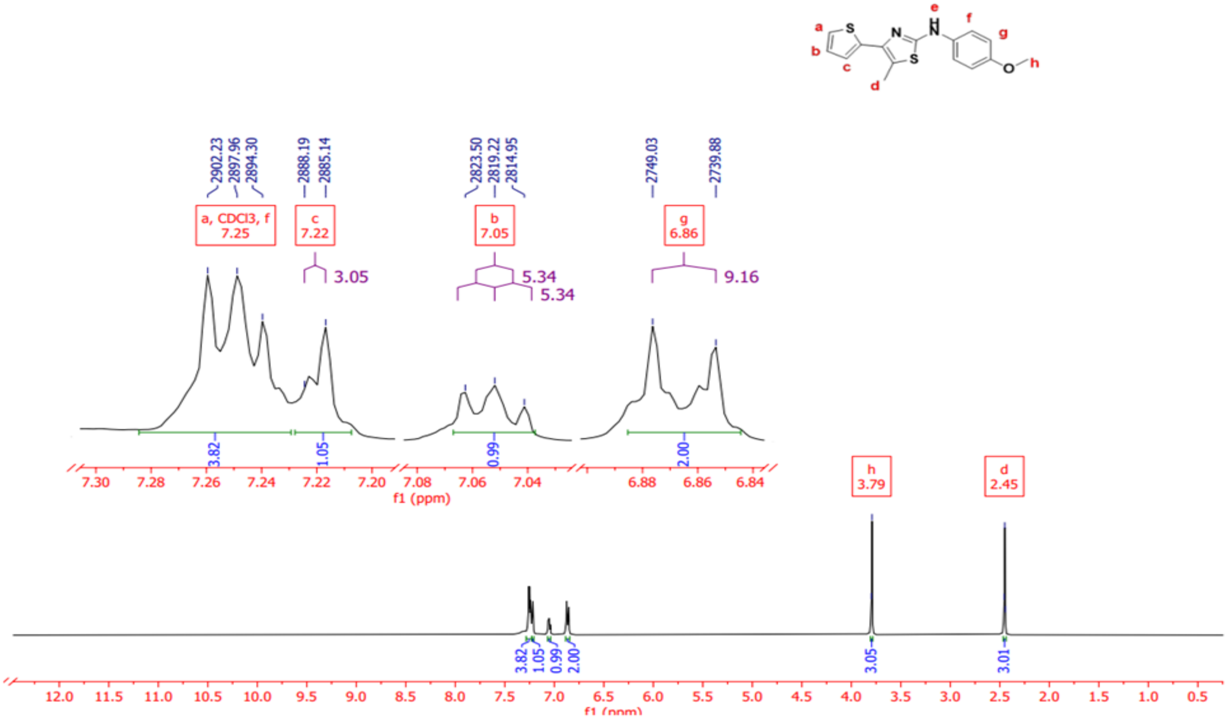

Figure S37. ^{1}H NMR spectrum of compound **C5** (400 MHz, $CDCl_3$)

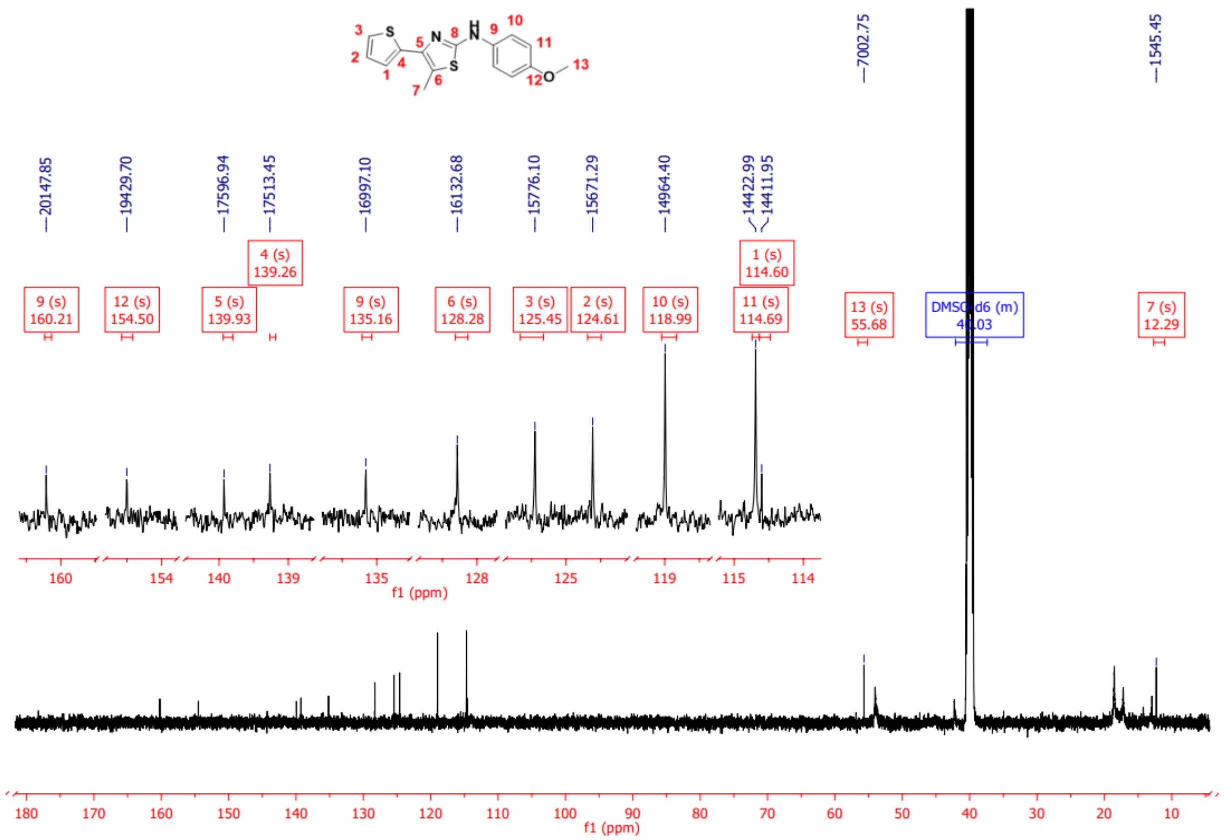

Figure S38. ^{13}C NMR spectrum of compound **C5** (125 MHz, $CDCl_3$)

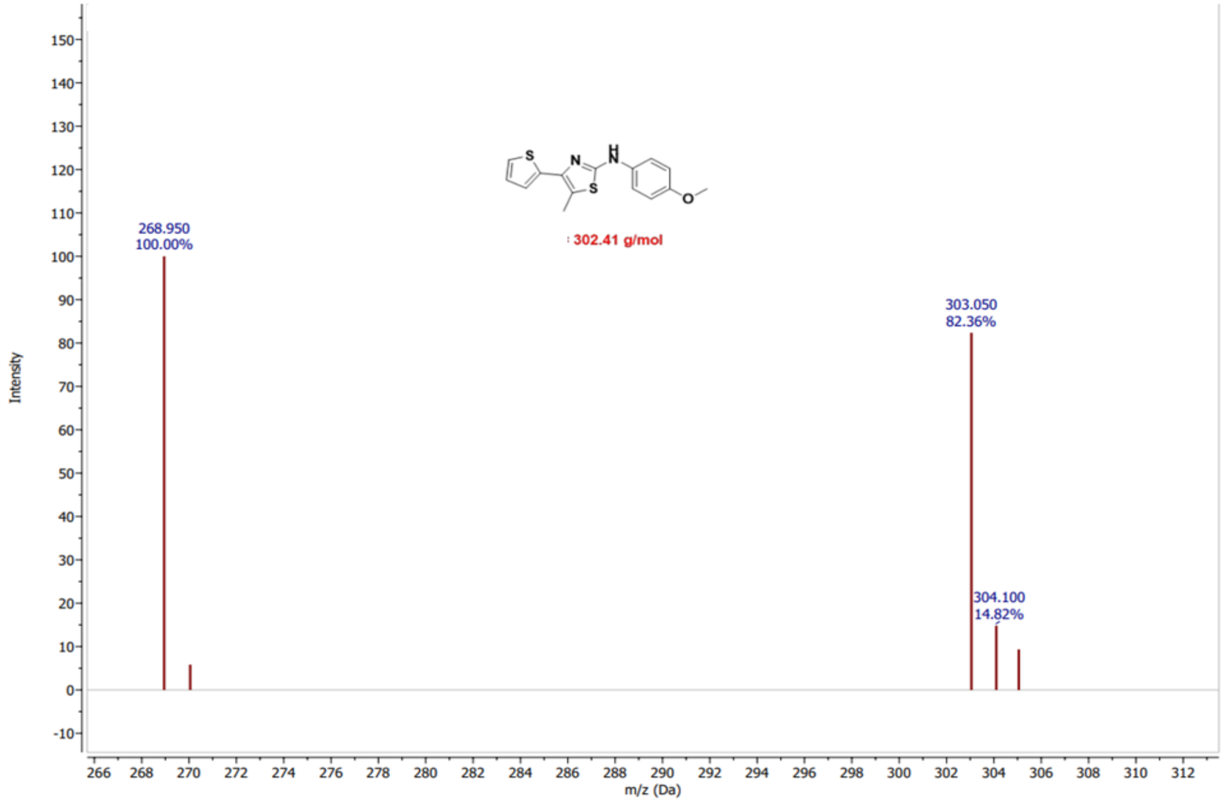

Figure S39. LC-MS/MS spectrum of compound **C5**

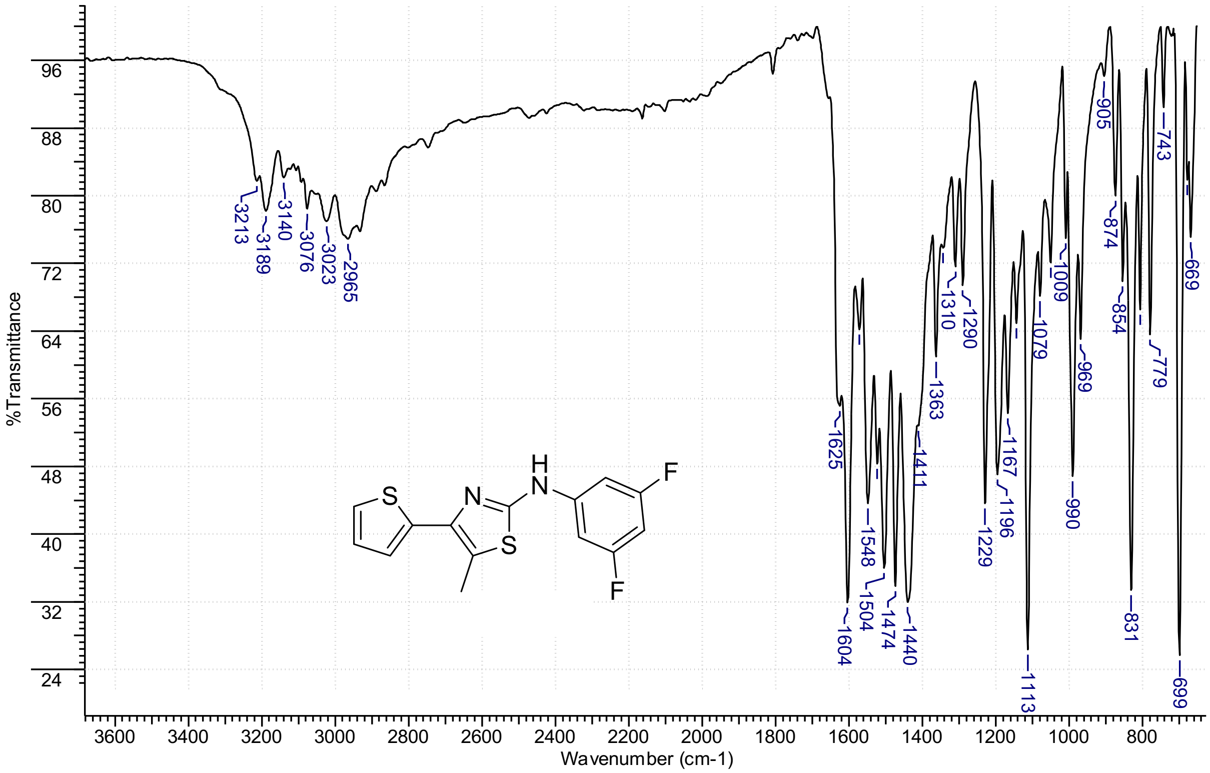

Figure S40. FT-IR spectrum of compound **C6**

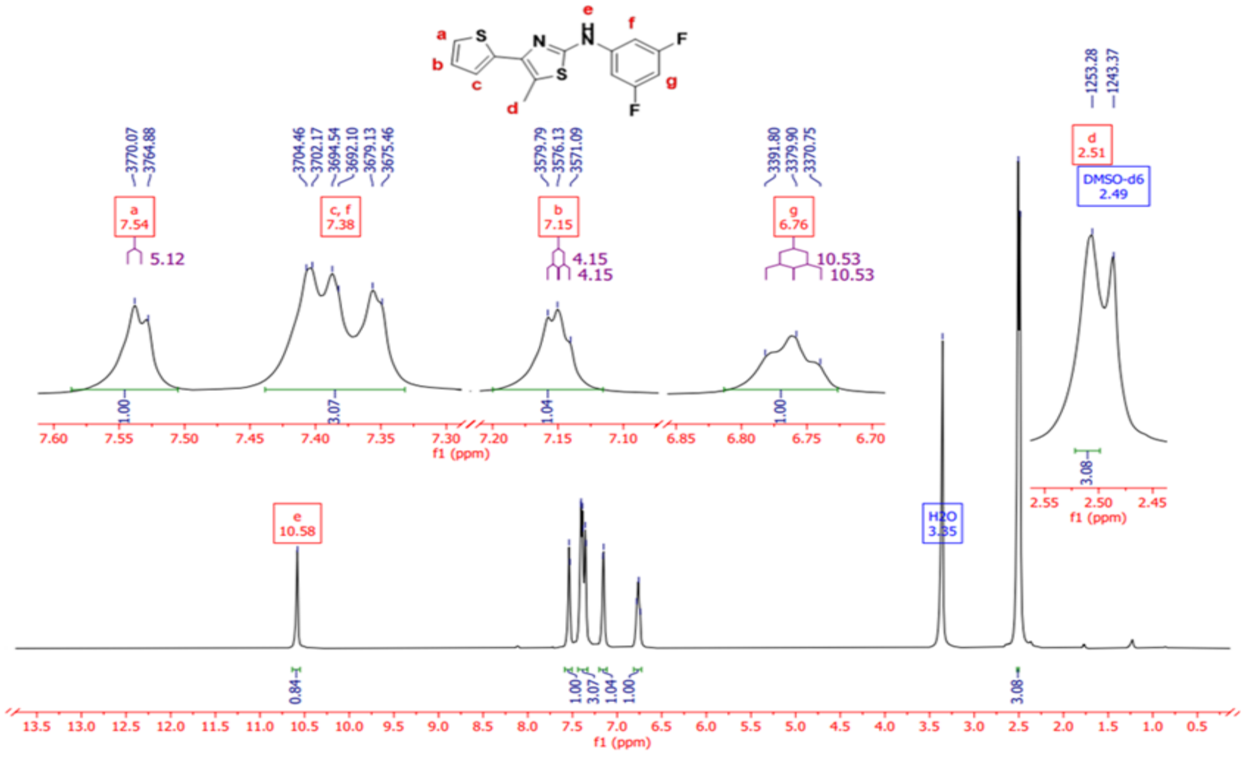

Figure S41. ^{1}H NMR spectrum of compound **C6** (400 MHz, $CDCl_3$)

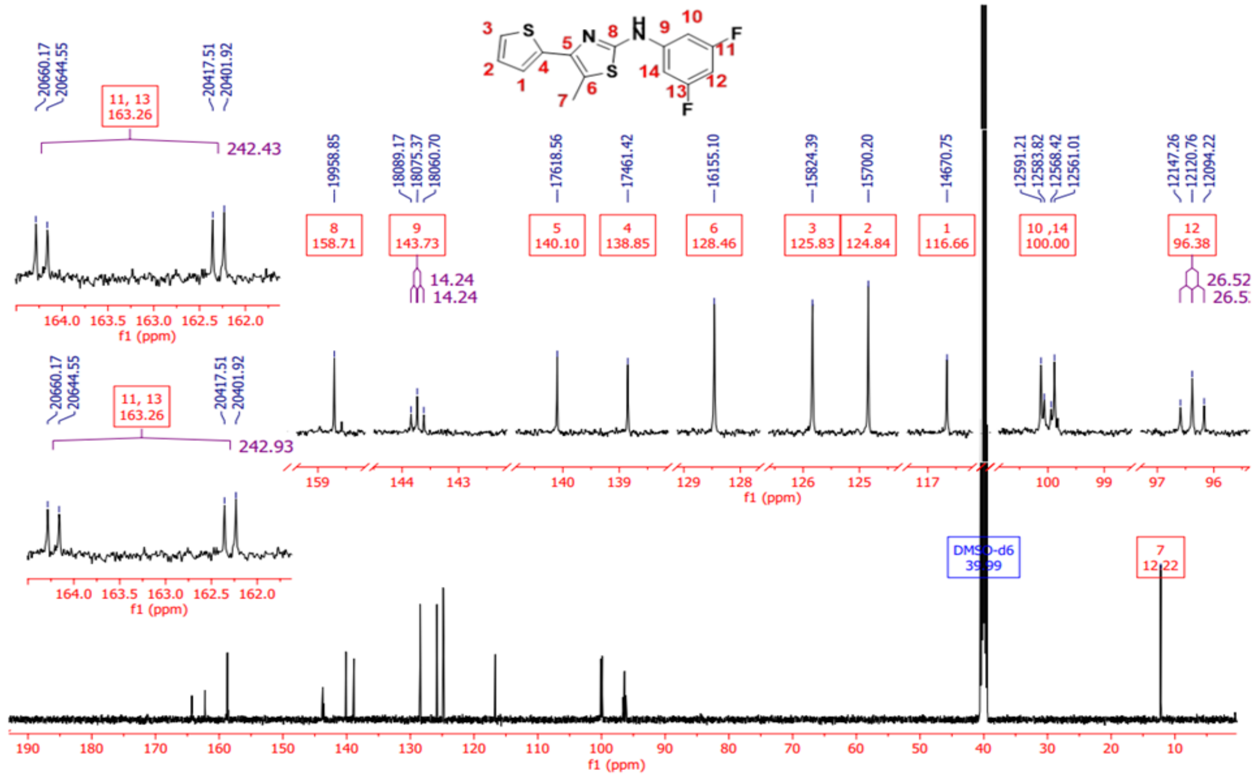

Figure S42. ^{13}C NMR spectrum of compound **C6** (125 MHz, $CDCl_3$)

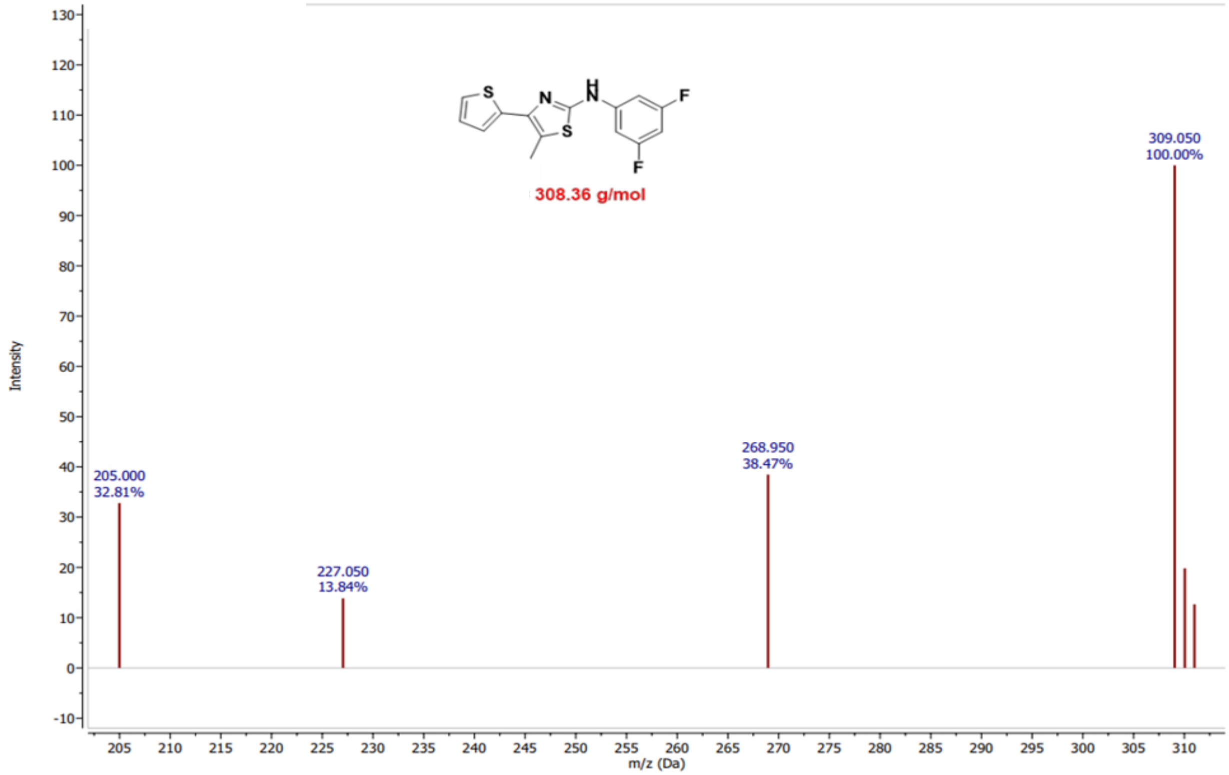

Figure S43. LC-MS/MS spectrum of compound **C6**

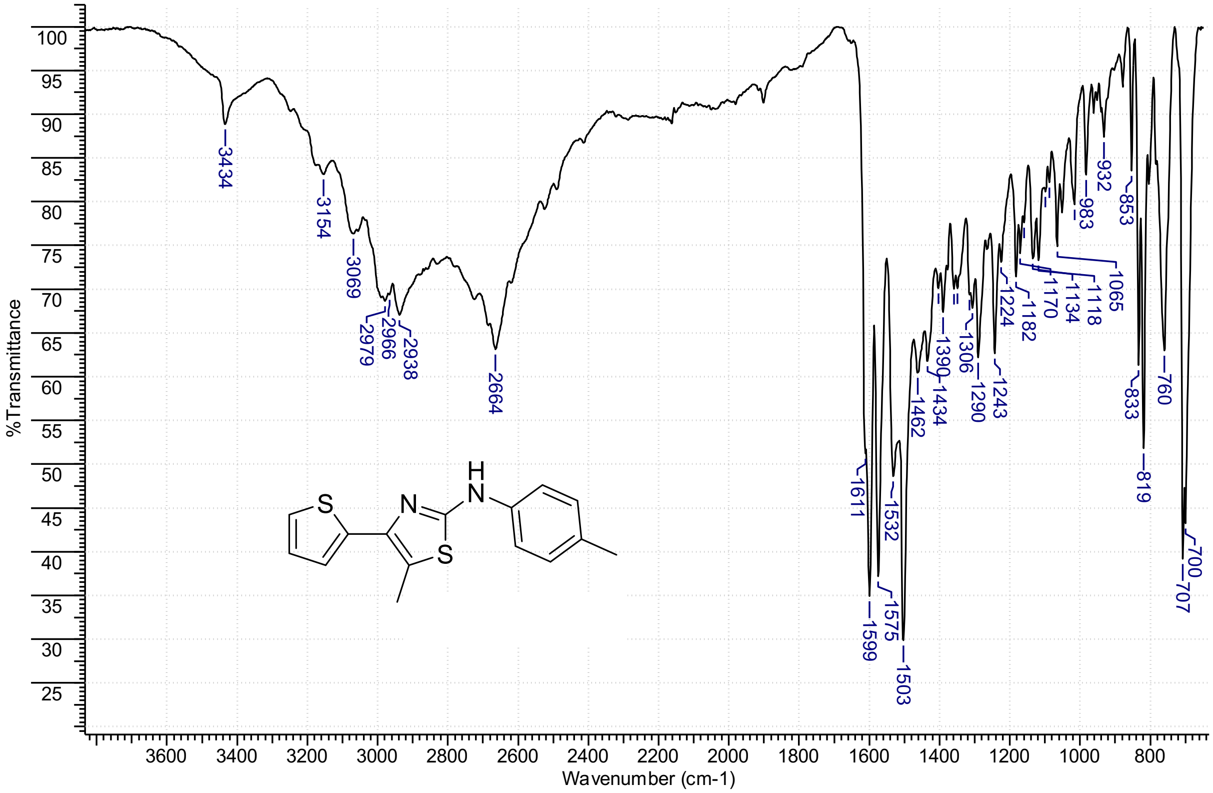

Figure S44. FT-IR spectrum of compound **C7**

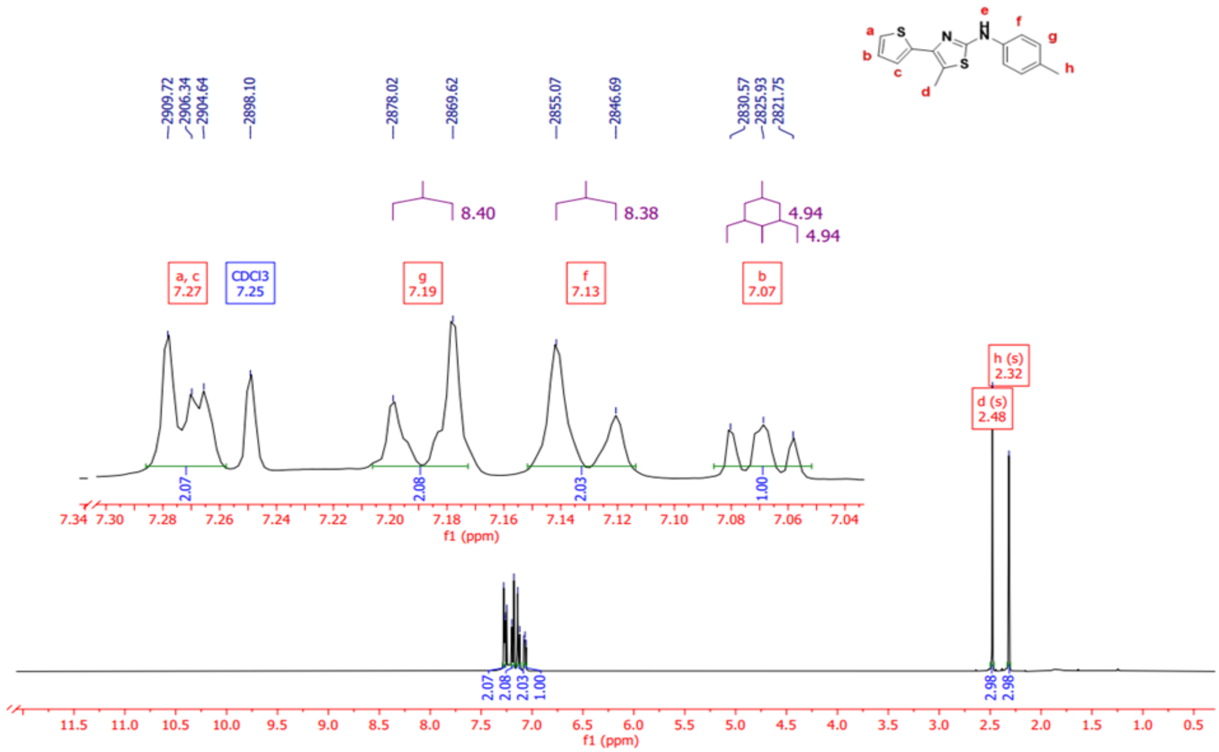

Figure S45. ^{1}H NMRspectrum of compound **C7** (400 MHz, $CDCl_3$)

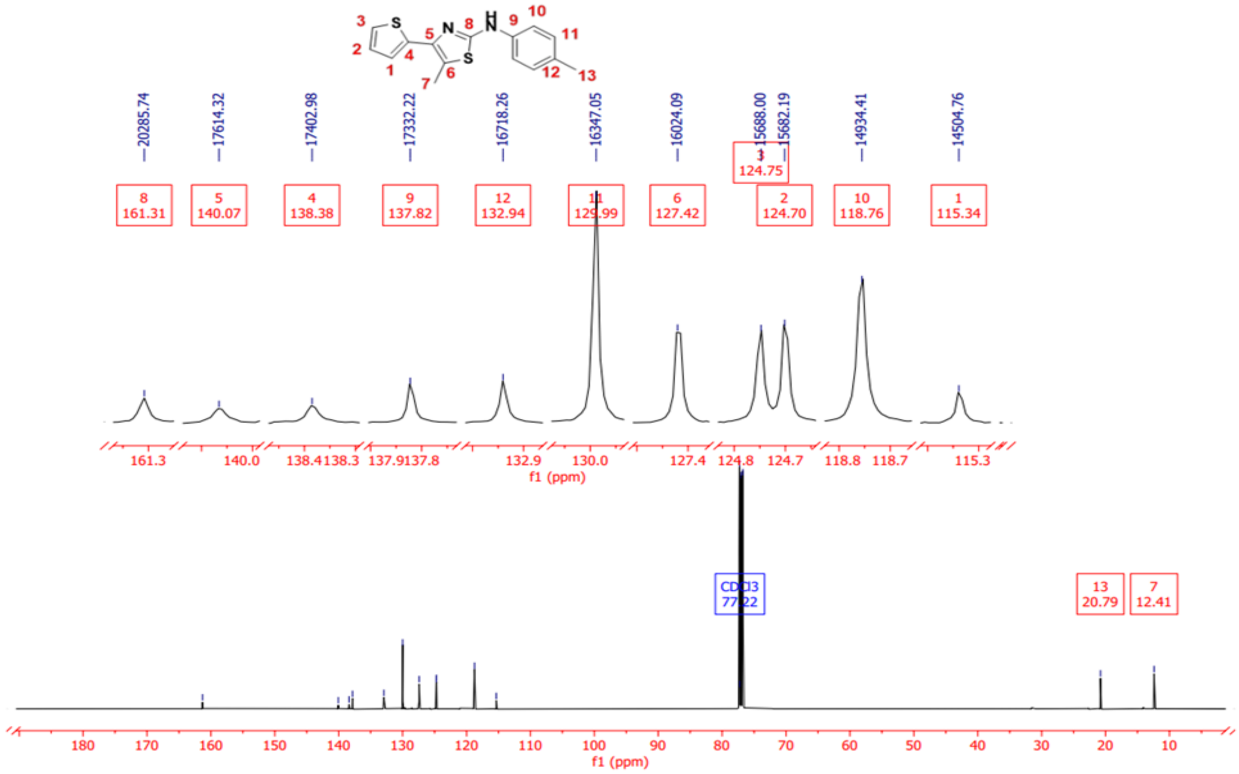

Figure S46. ^{13}C NMRspectrum of compound **C7** (125 MHz, $CDCl_3$)

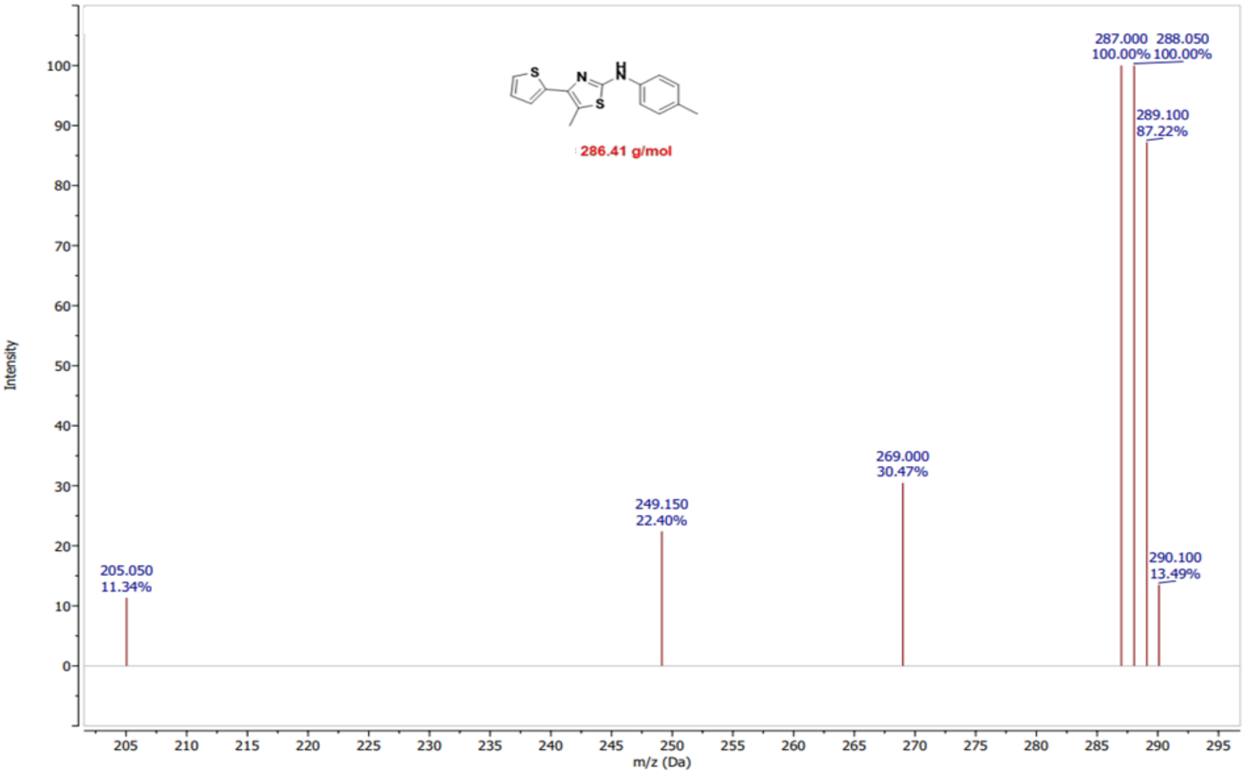

Figure S47. LC-MS/MS spectrum of compound **C7**

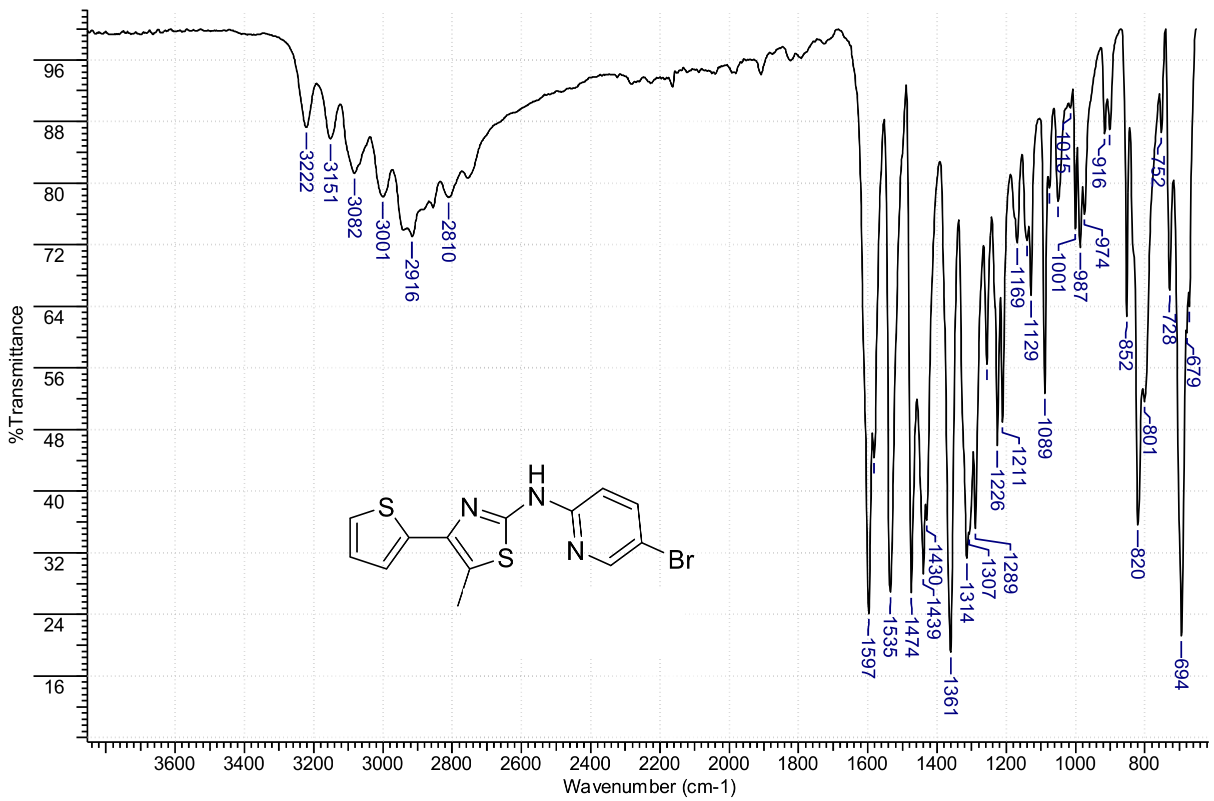

Figure S48. FT-IR spectrum of compound **C8**

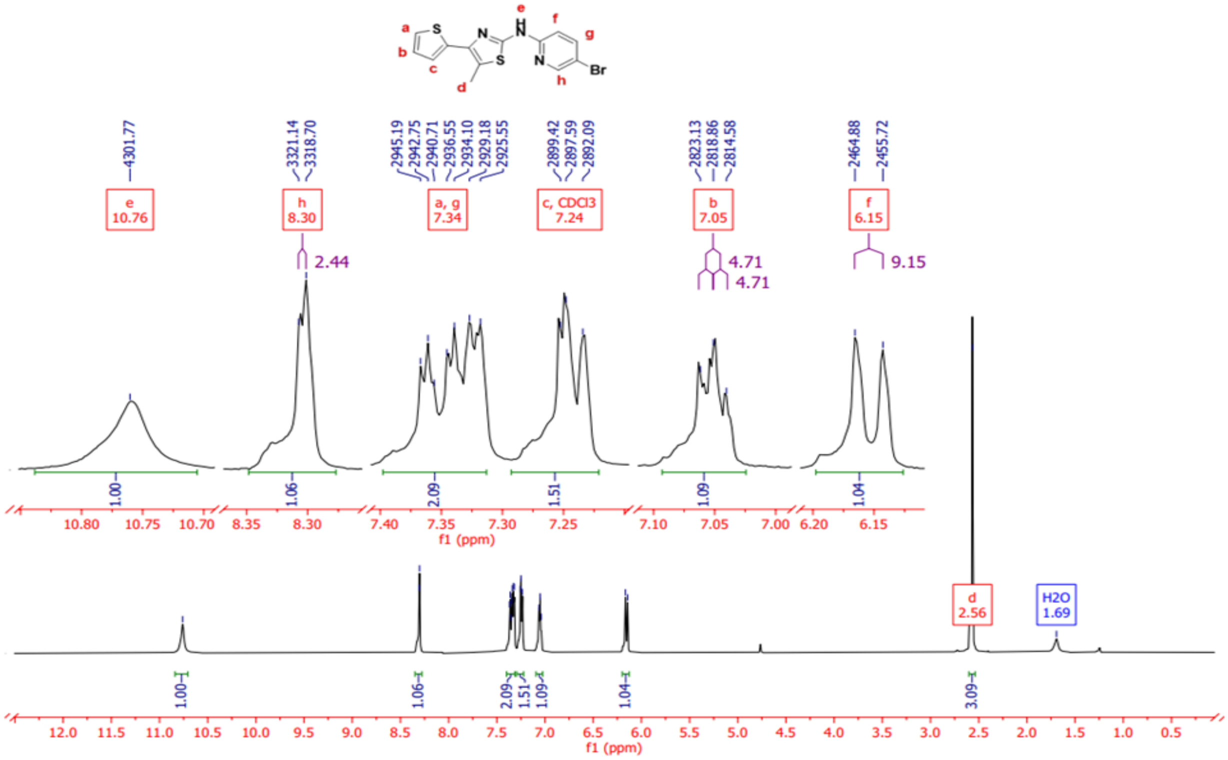

Figure S49. ^{1}H NMR spectrum of compound **C8** (400 MHz, $CDCl_3$)

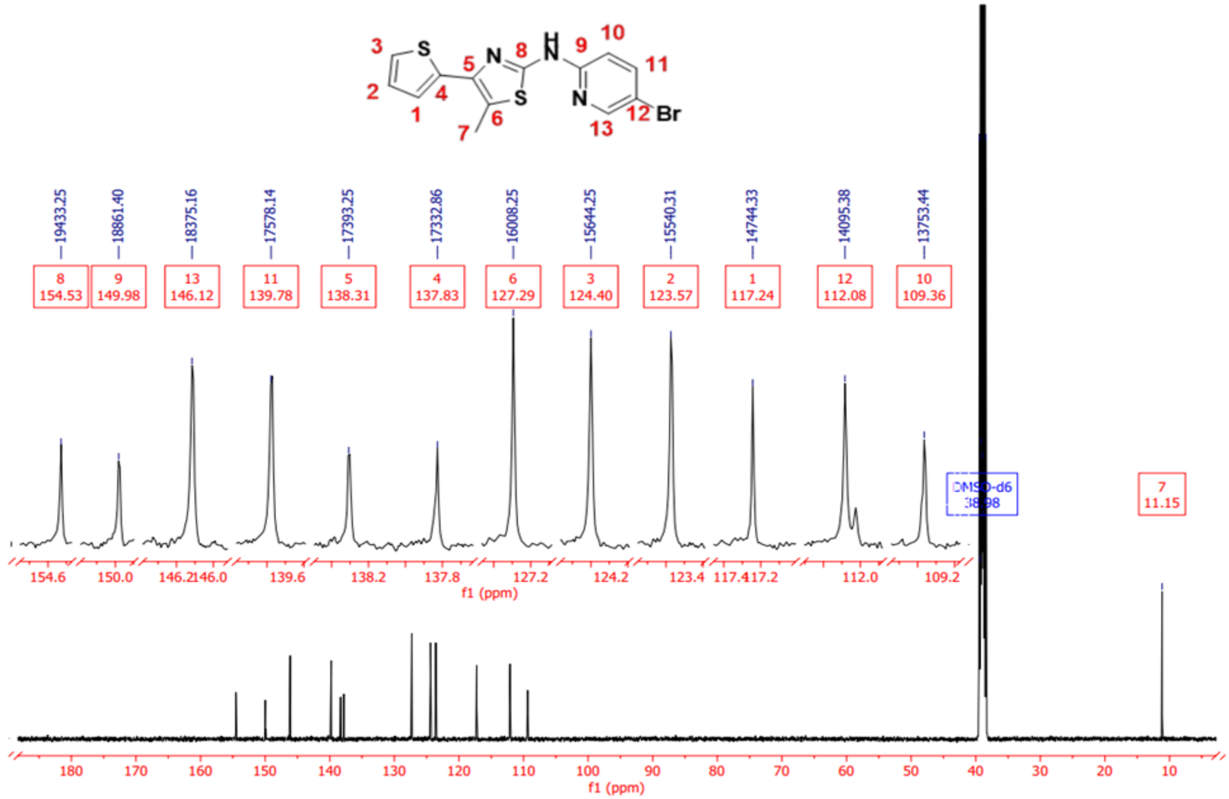

Figure S50. ^{13}C NMRspectrum of compound **C8** (125 MHz, $CDCl_3$)

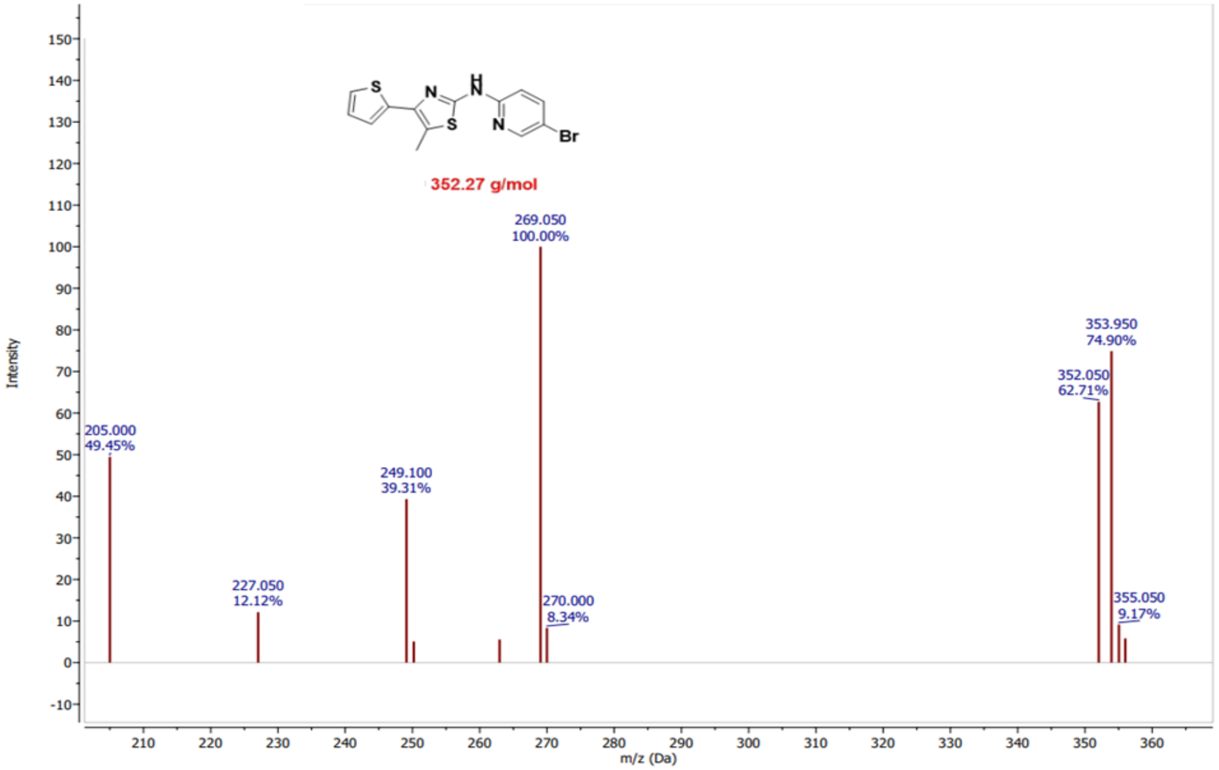

Figure S51. LC-MS/MS spectrum of compound **C8**

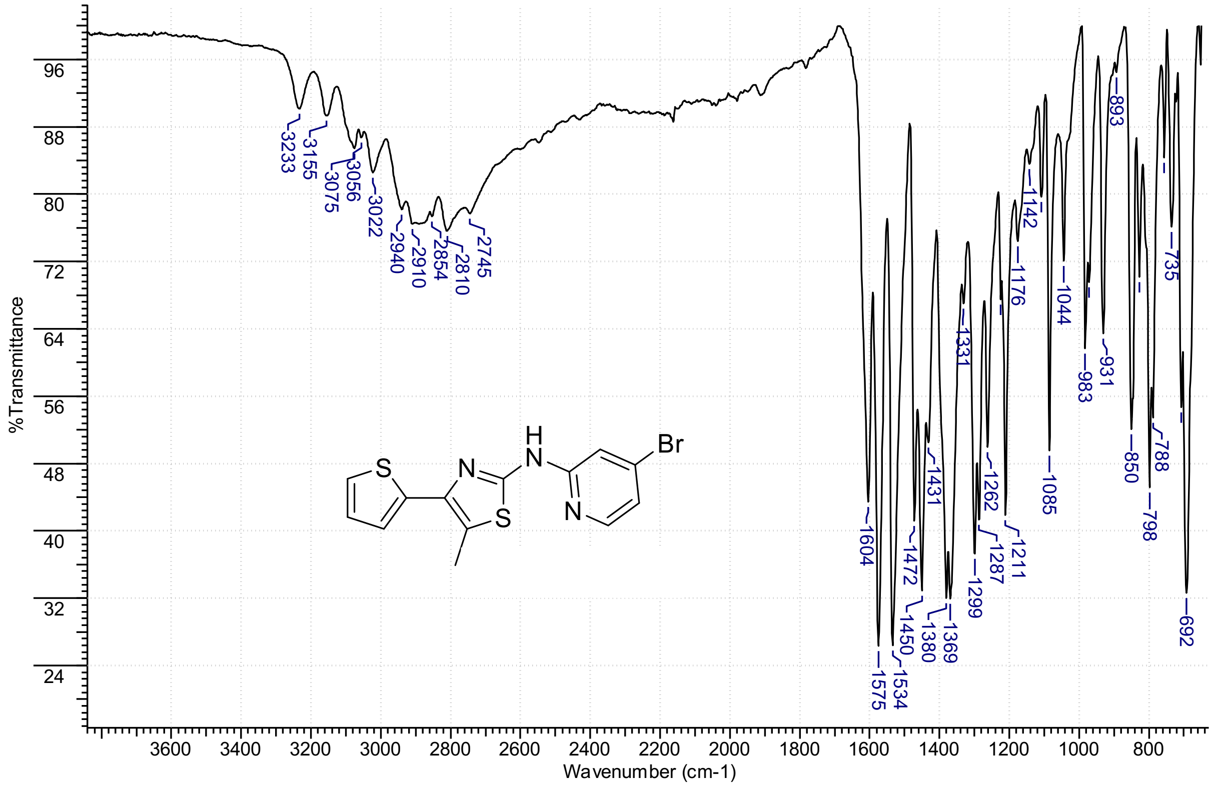

Figure S52. FT-IR spectrum of compound **C9**

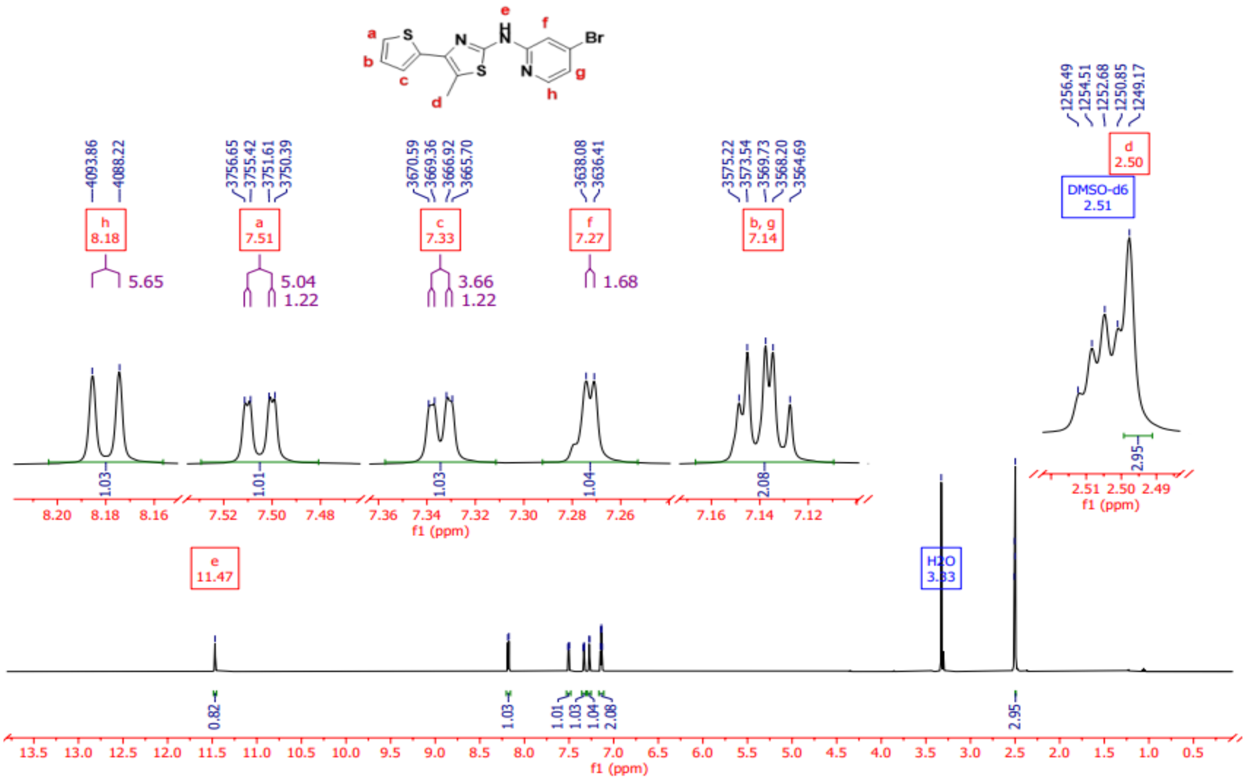

Figure S53. ^{1}H NMR spectrum of compound **C9** (500 MHz, DMSO-d_6)

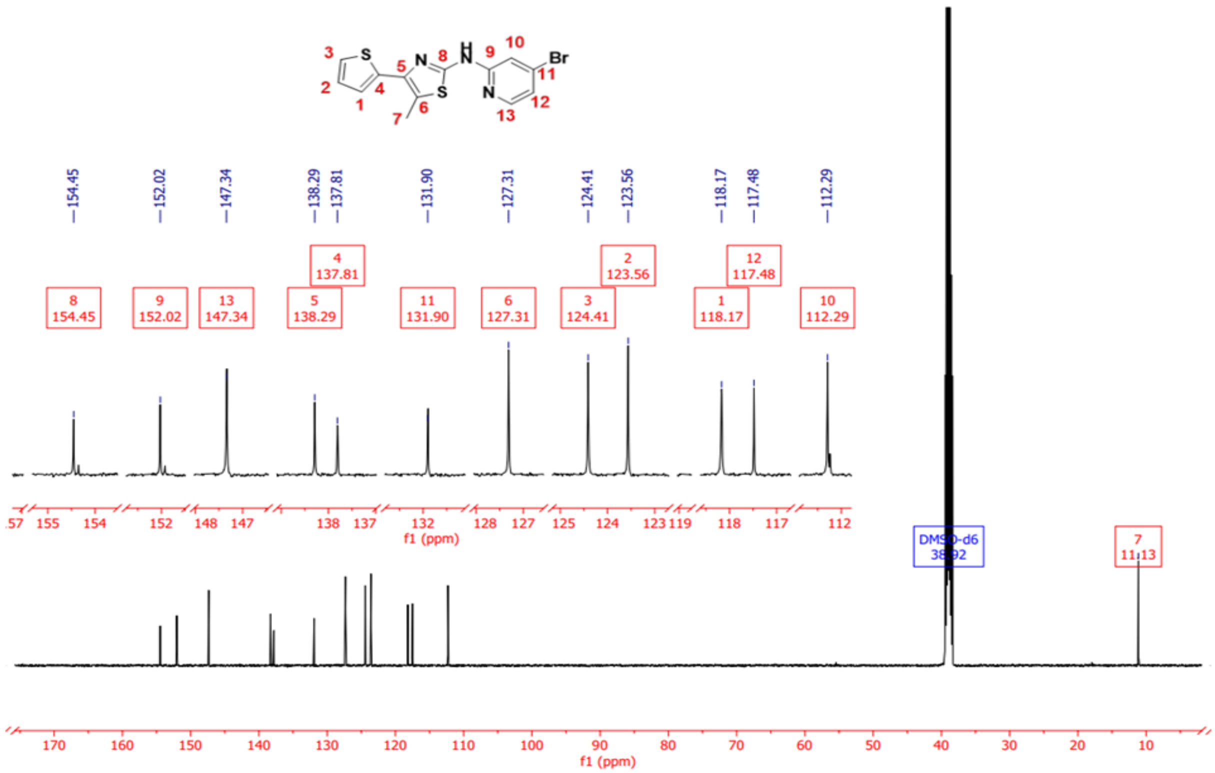

Figure S54. ^{13}C NMRspectrum of compound **C9** (125 MHz, DMSO-d$_6$)

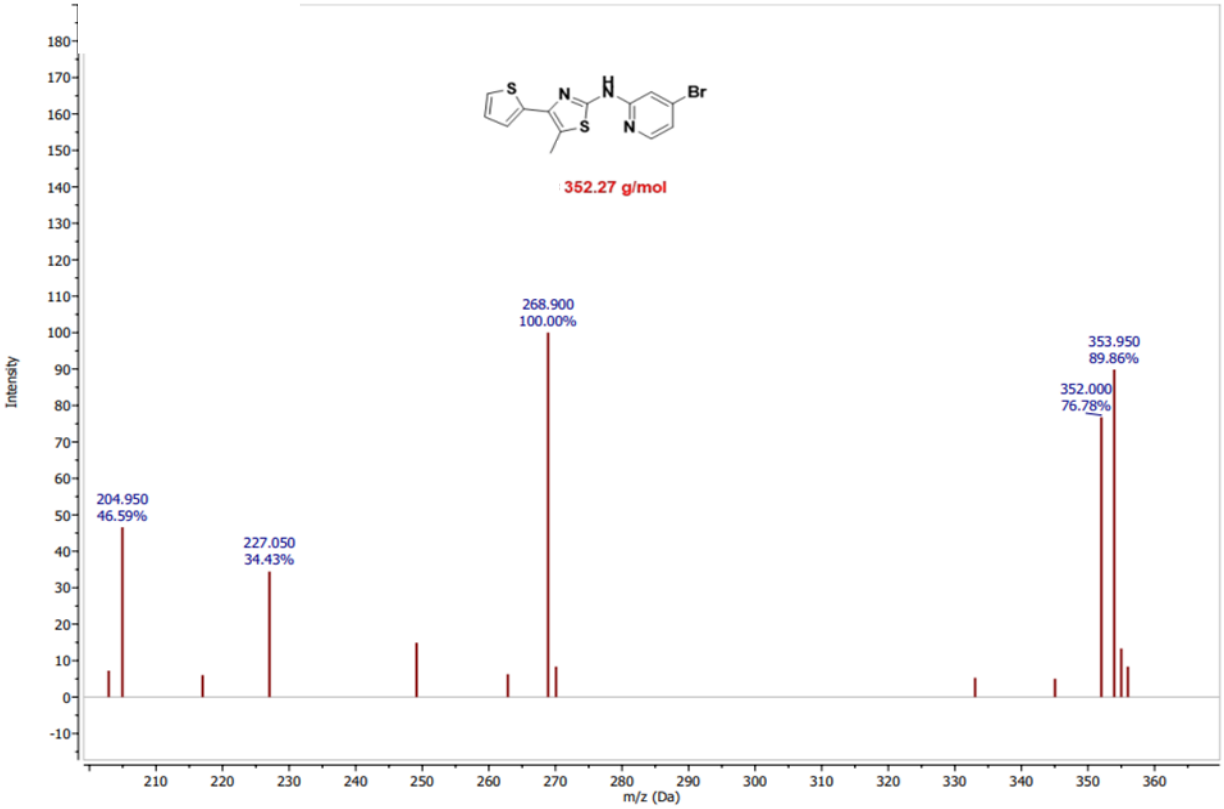

Figure S55. LC-MS/MS spectrum of compound **C9**

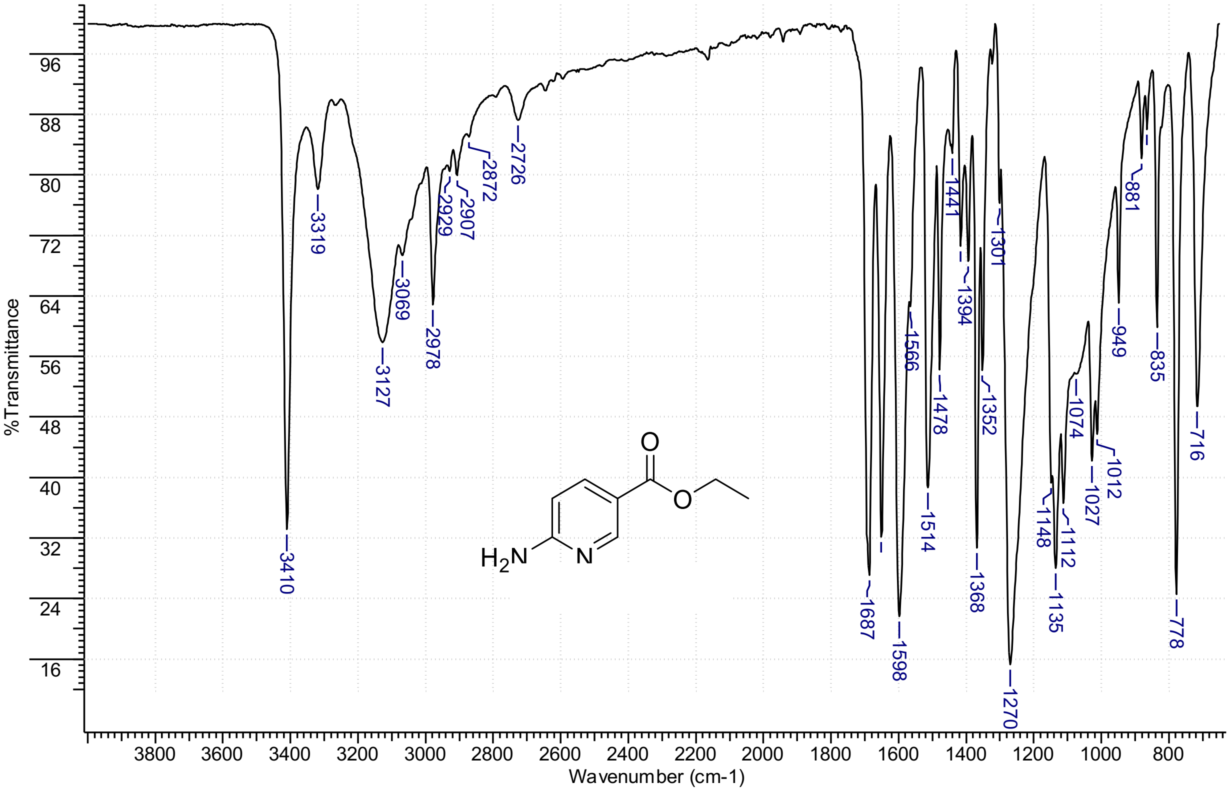

Figure S56. FT-IR spectrum of intermediate 8

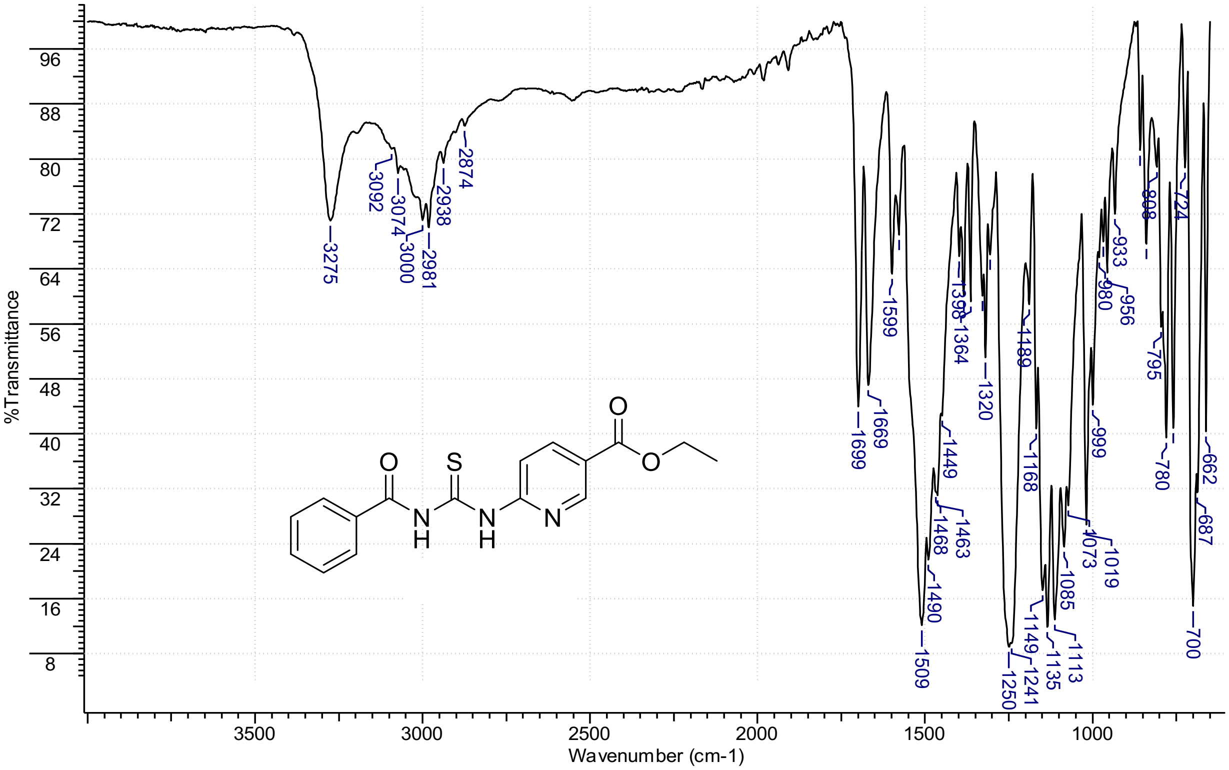

Figure S57. FT-IR spectrum of intermediate 9

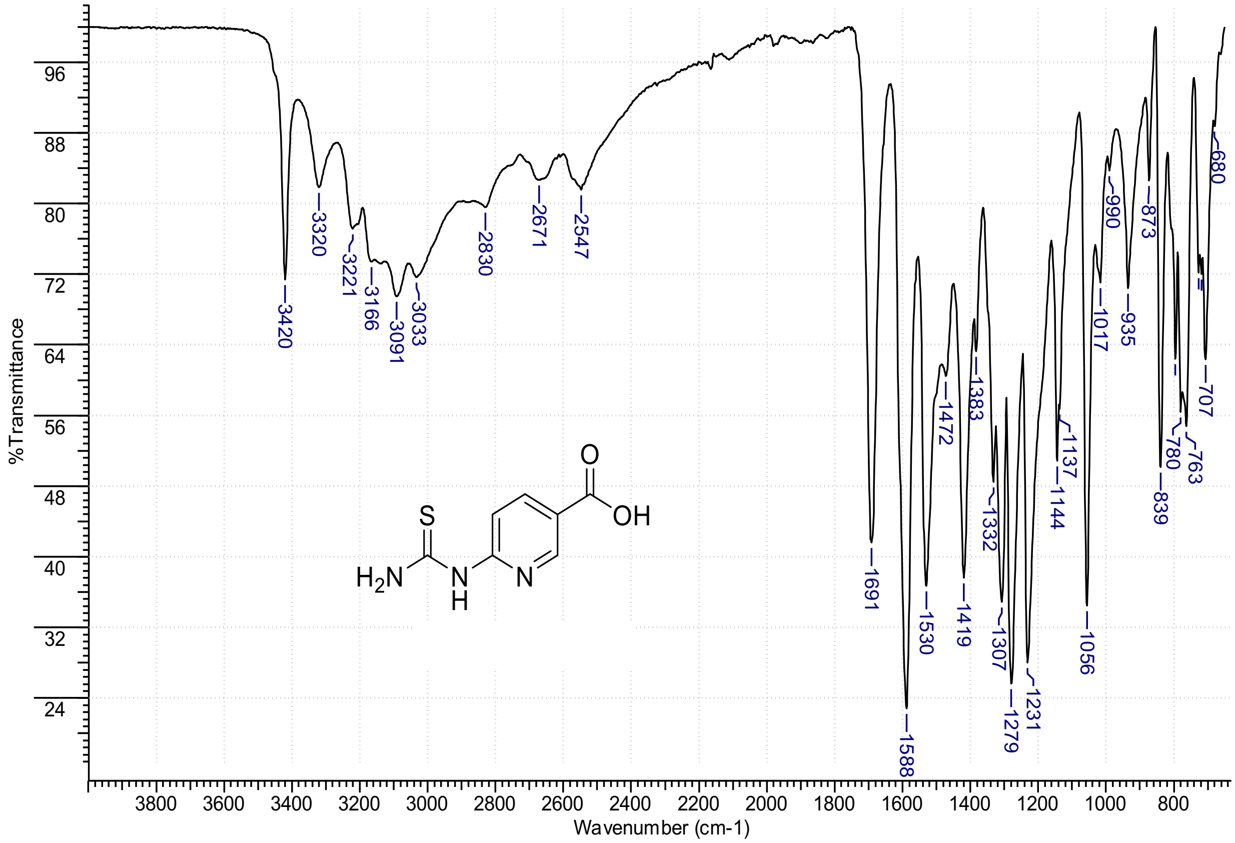

Figure S58. FT-IR spectrum of intermediate 10

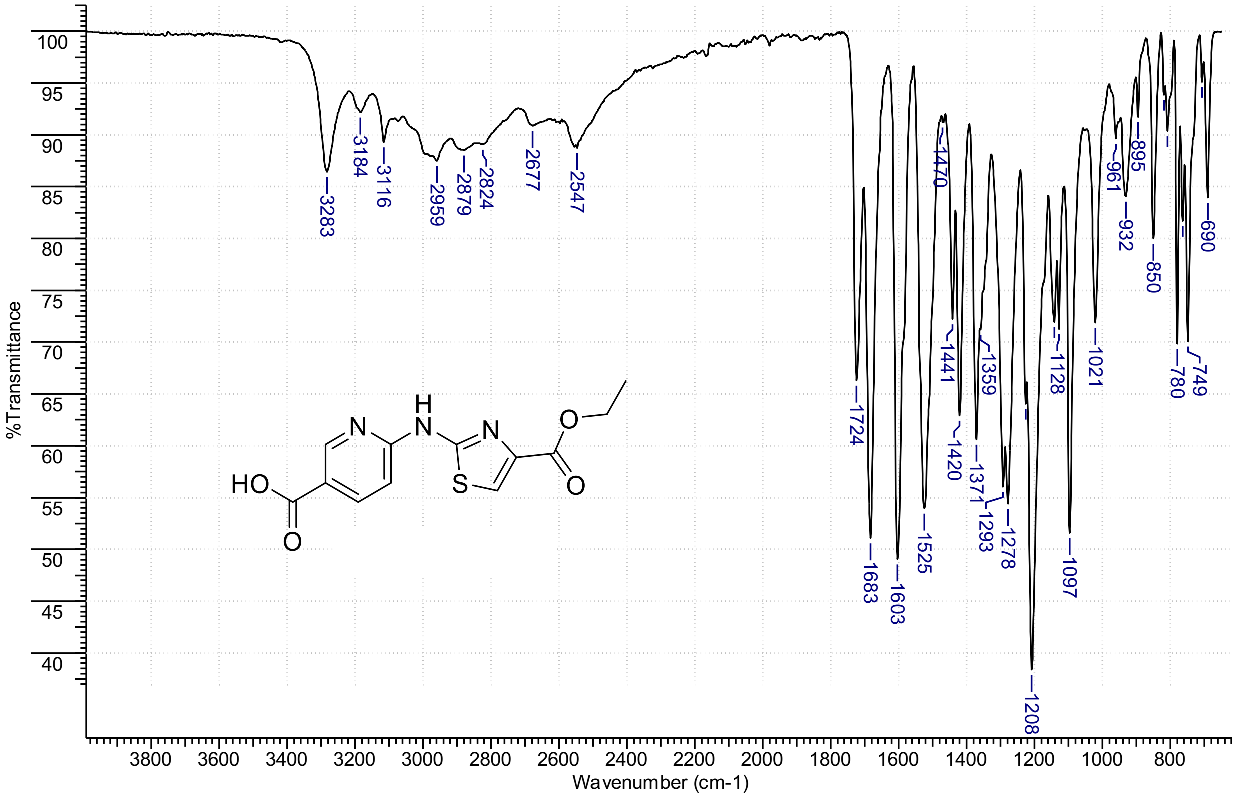

Figure S59. FT-IR spectrum of intermediate Ca

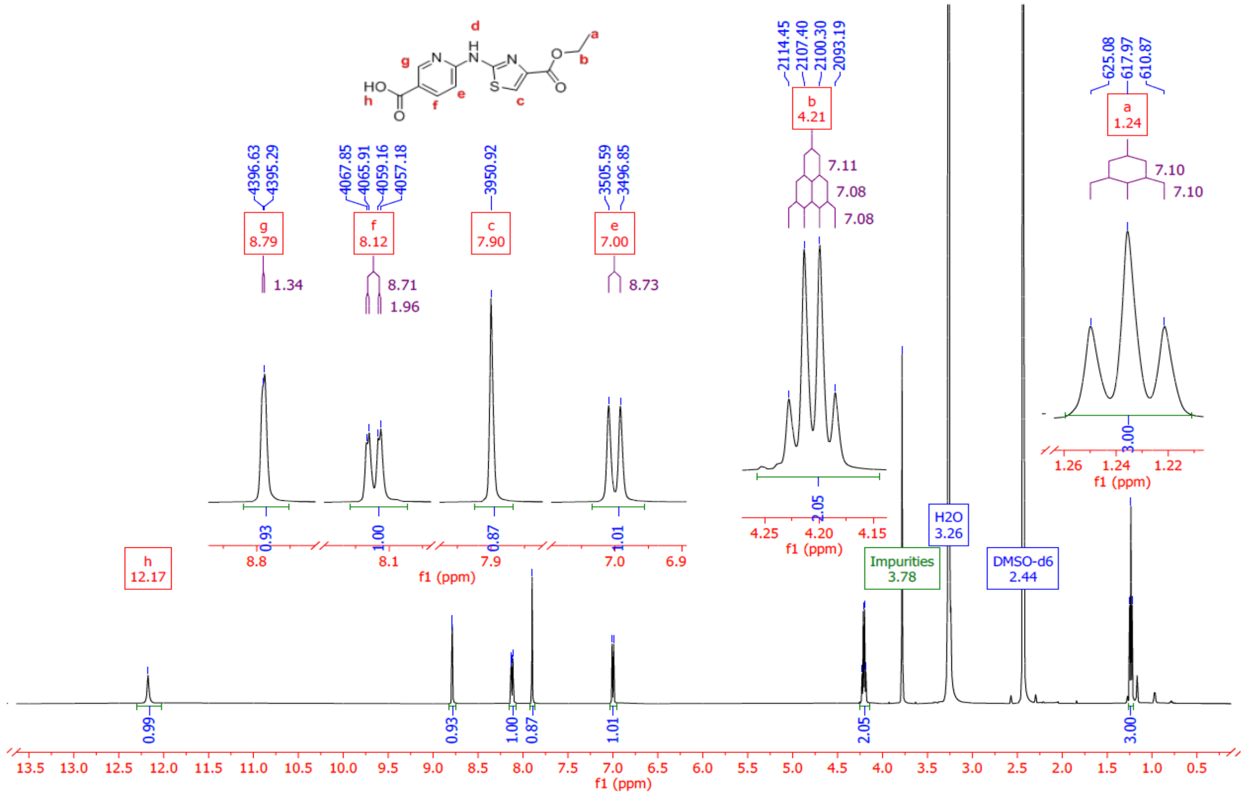

Figure S60. ^{1}H NMR spectrum of intermediate Ca (500 MHz, DMSO-d_6)

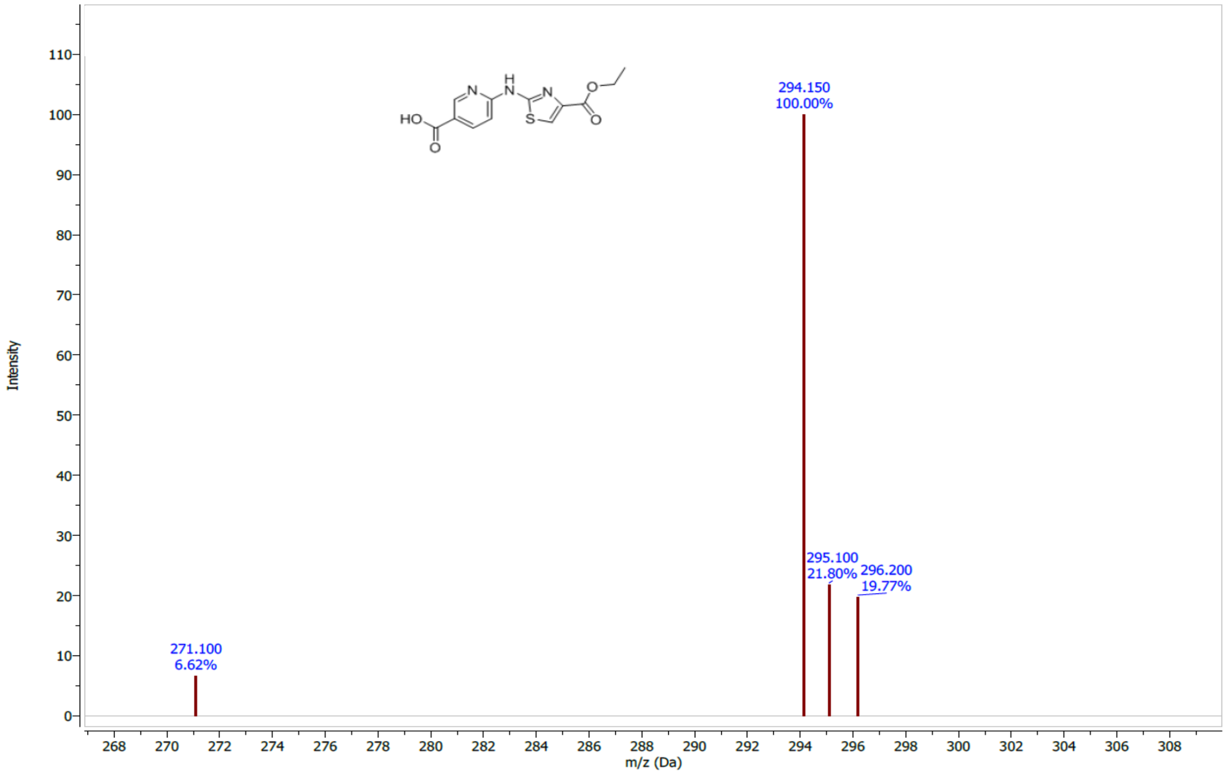

Figure S61. LC-MS/MS spectrum of intermediate Ca

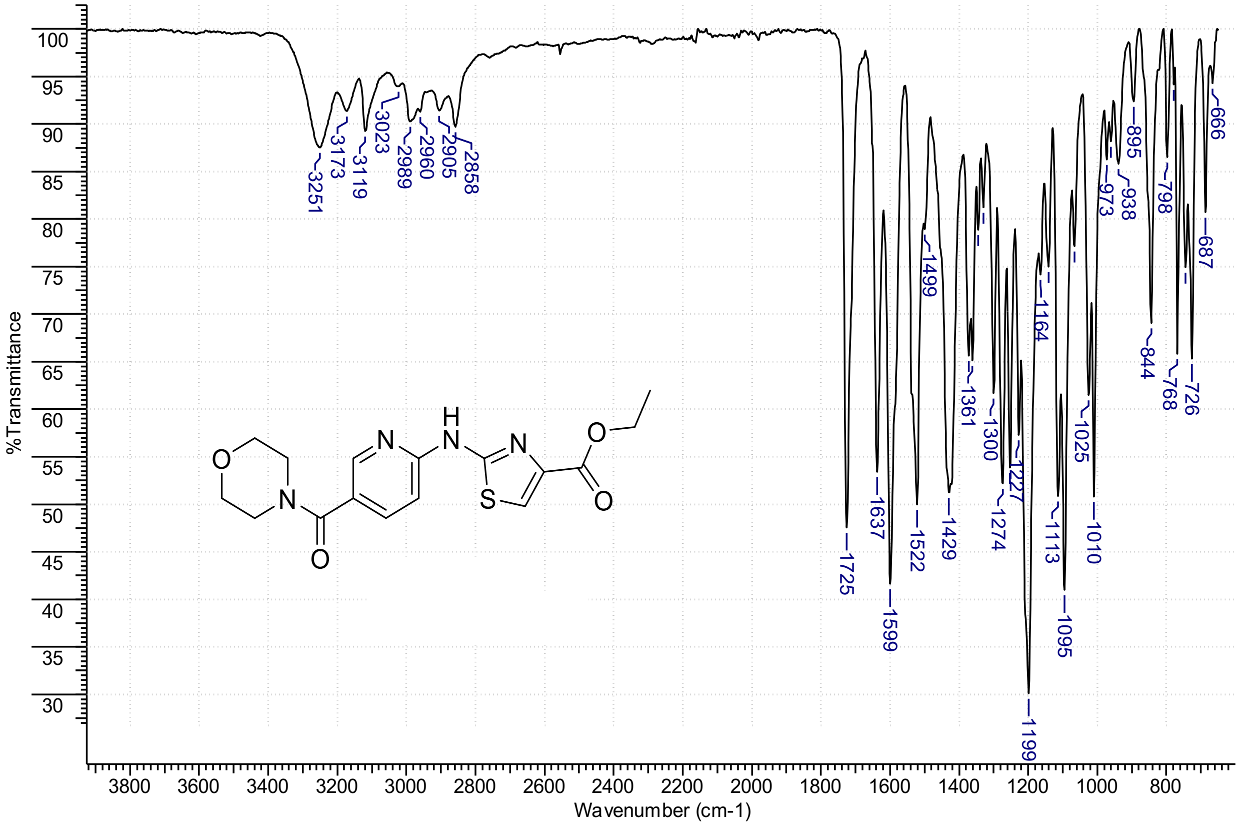

Figure S62. FT-IR spectrum of intermediate Cb

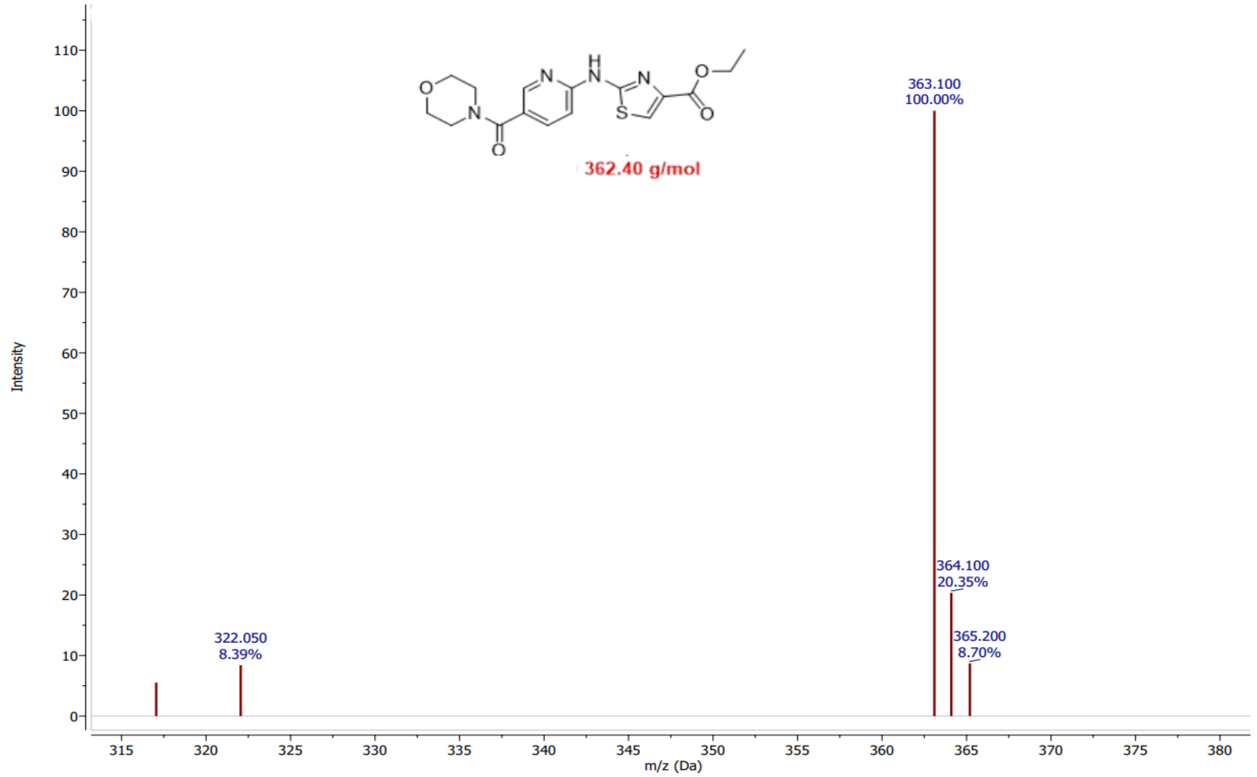

Figure S63. LC-MS/MS spectrum of intermediate Cb

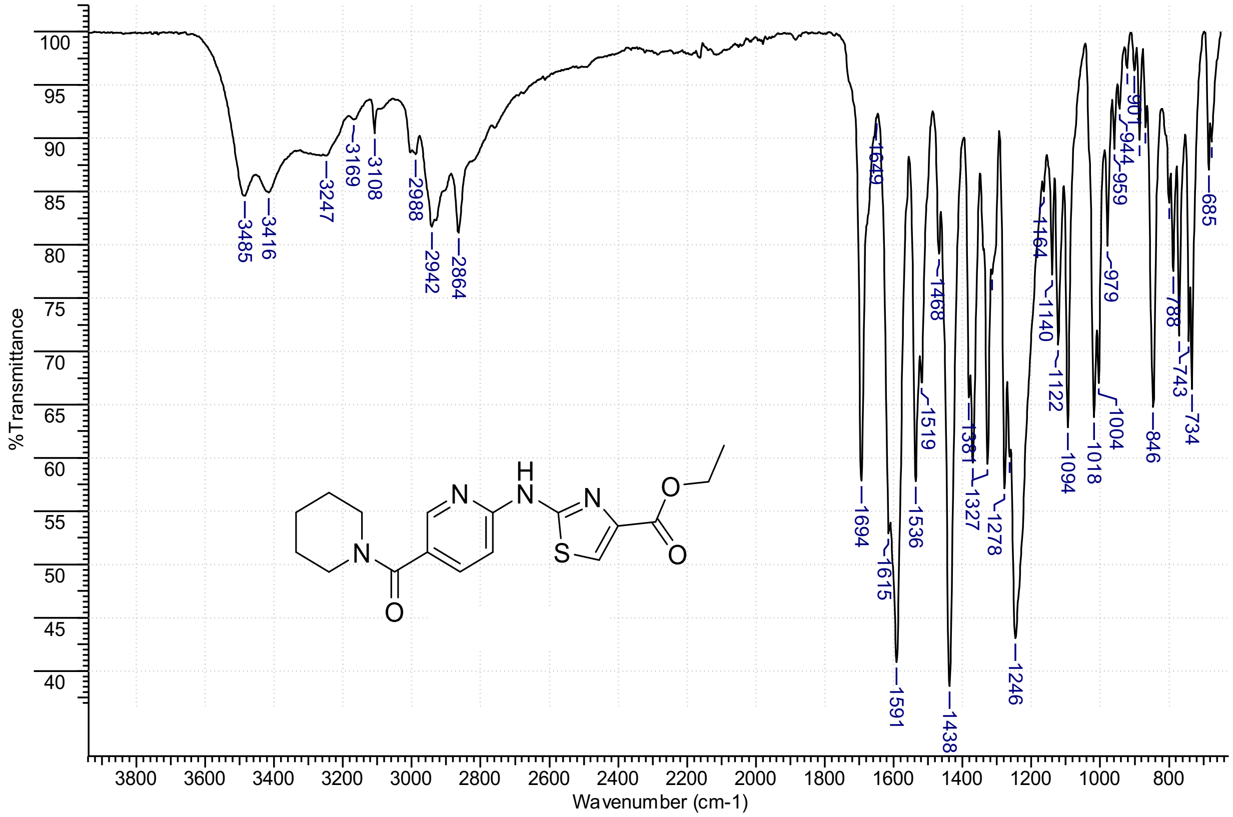

Figure S64. FT-IR spectrum of intermediate Cc

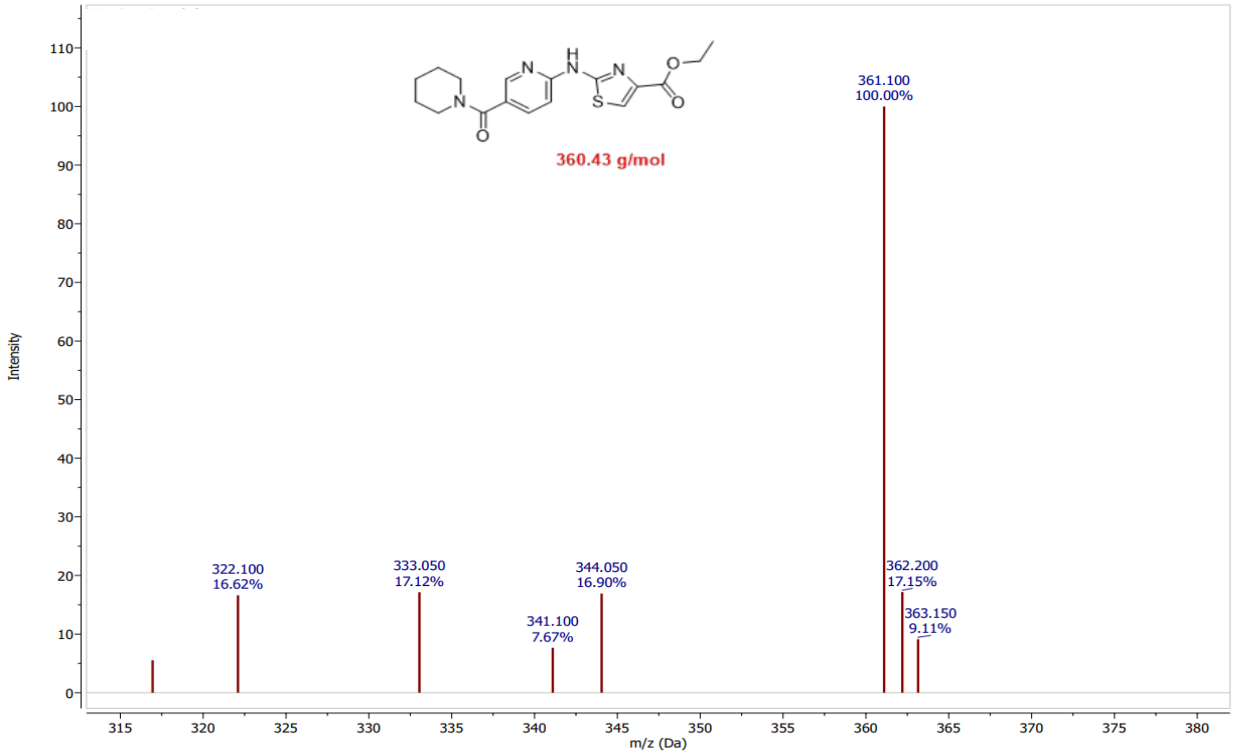

Figure S65. LC-MS/MS spectrum of intermediate Cc

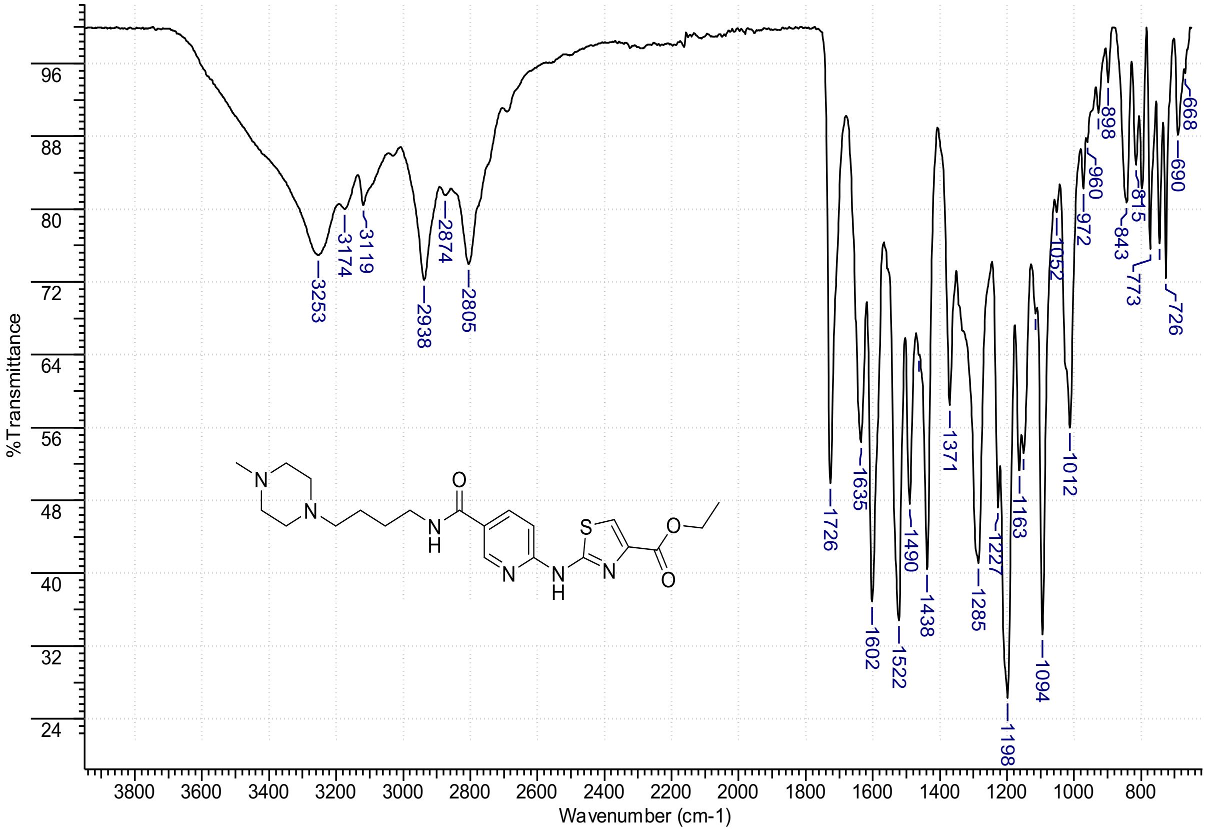

Figure S66. FT-IR spectrum of intermediate Cd

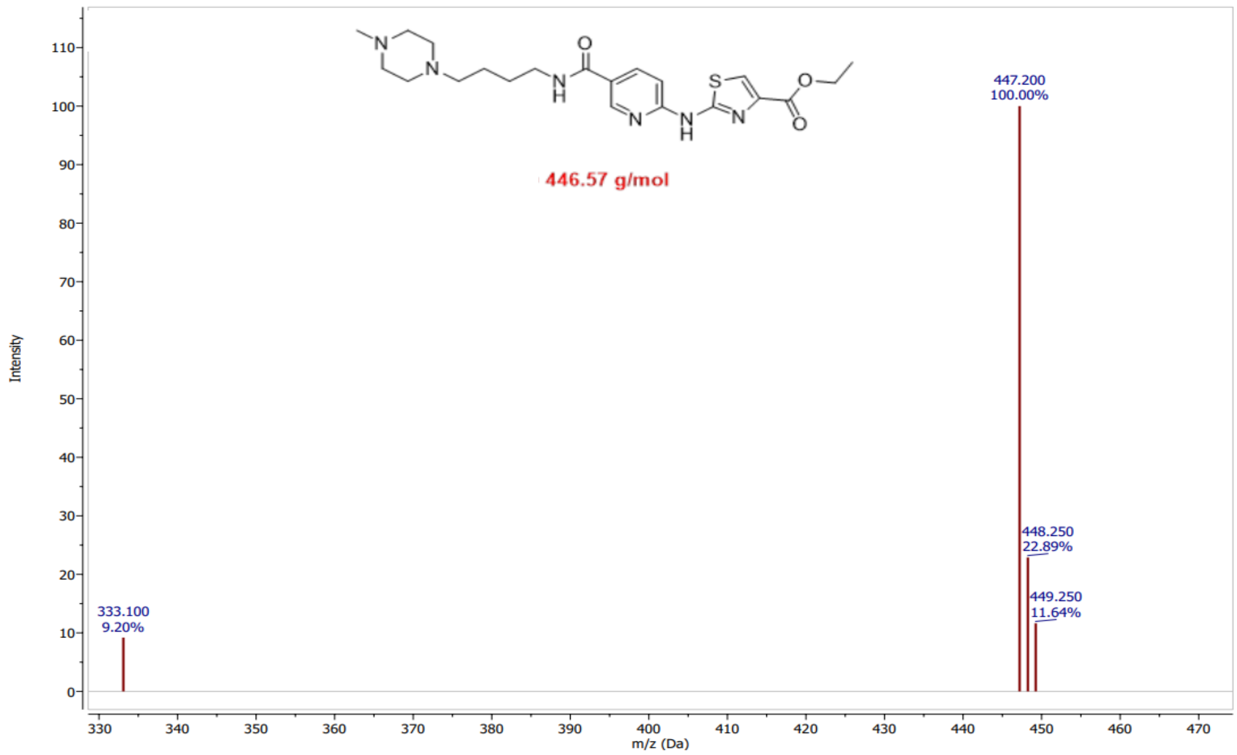

Figure S67. LC-MS/MS spectrum of intermediate Cd

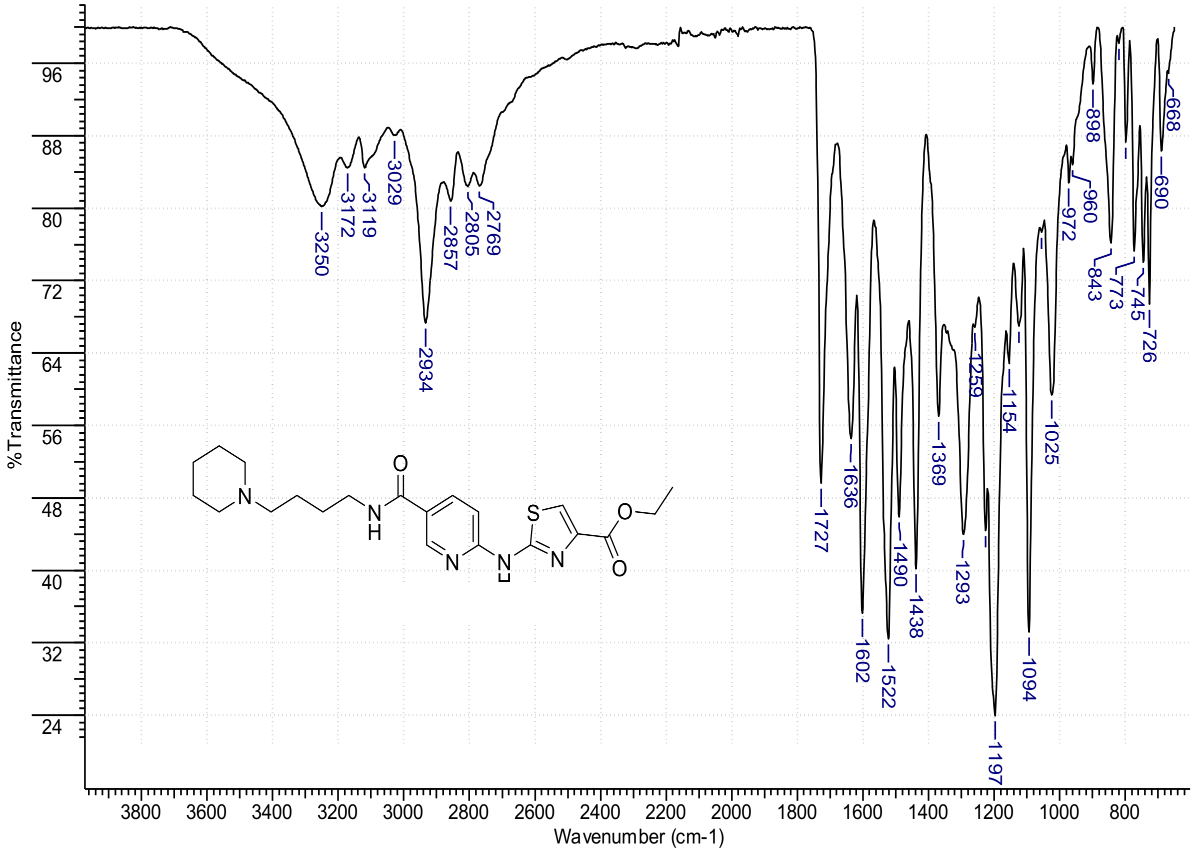

Figure S68. FT-IR spectrum of intermediate Ce

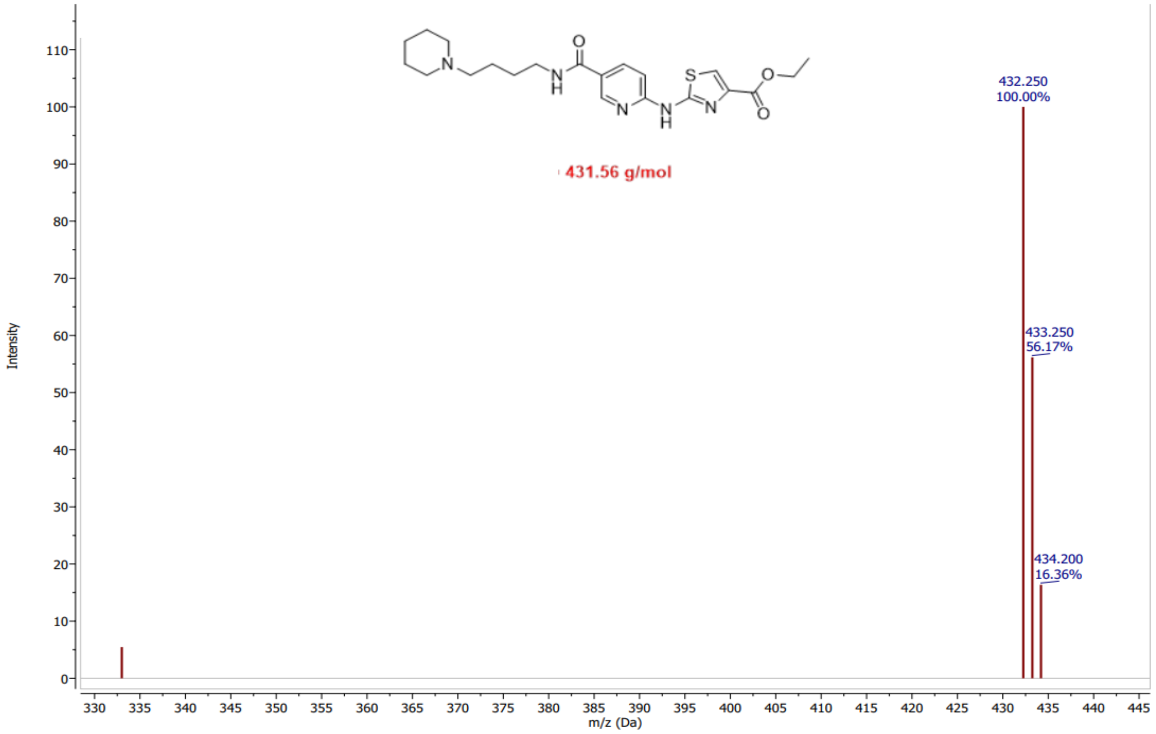

Figure S69. LC-MS/MS spectrum of intermediate Ce

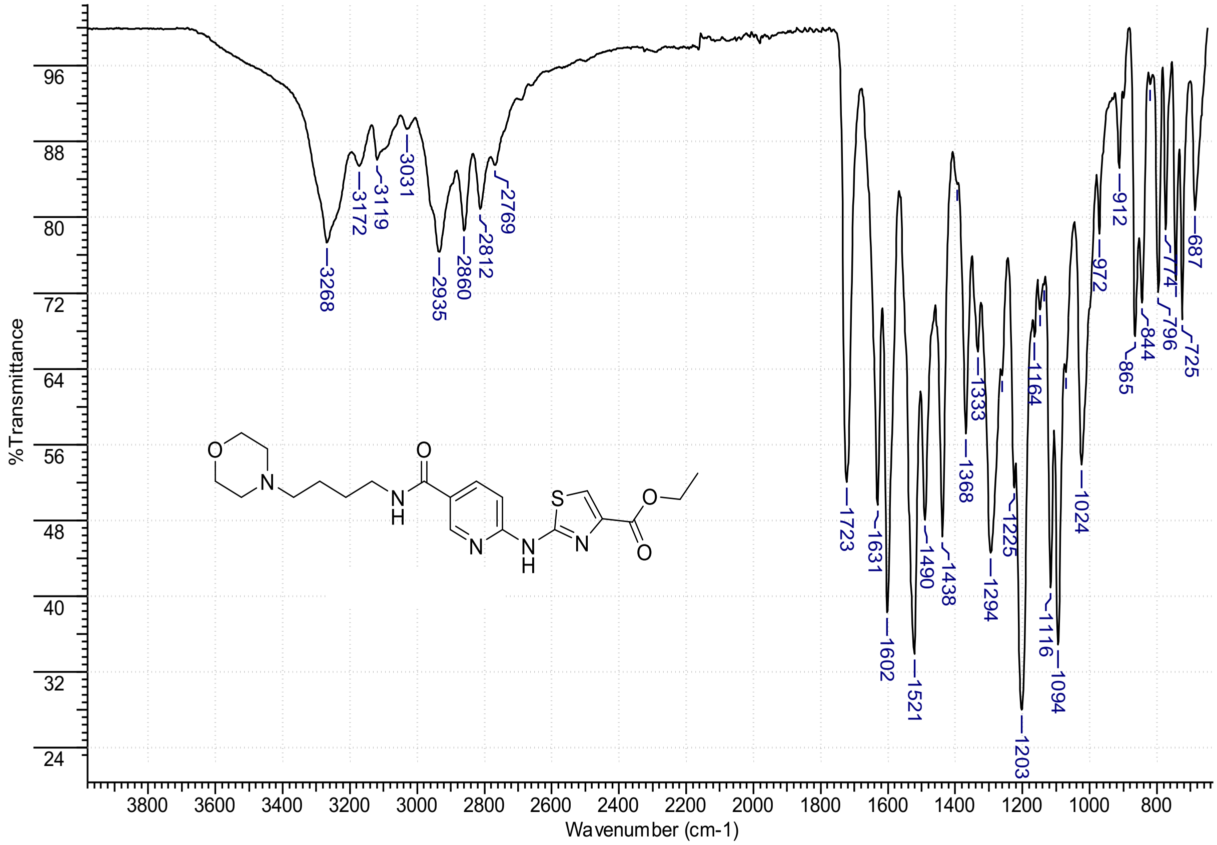

Figure S70. FT-IR spectrum of intermediate Cf

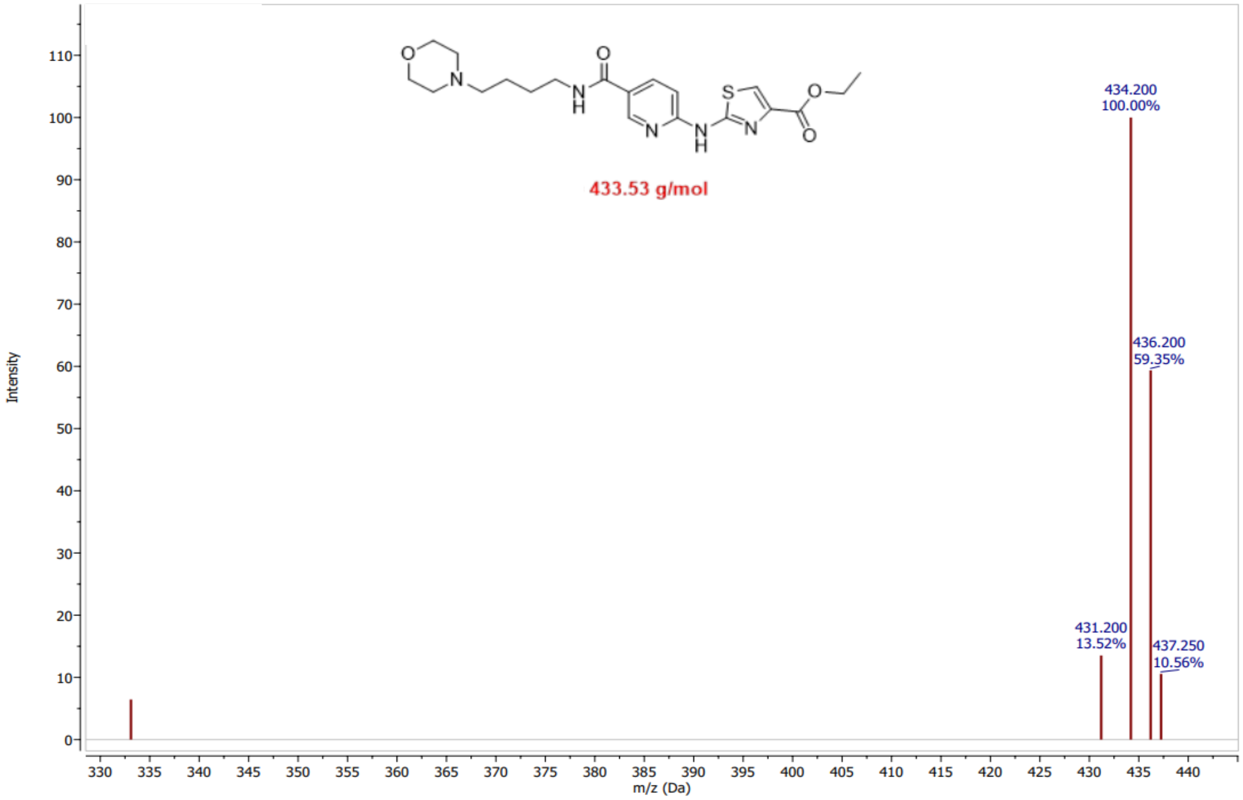

Figure S71. LC-MS/MS spectrum of intermediate Cf

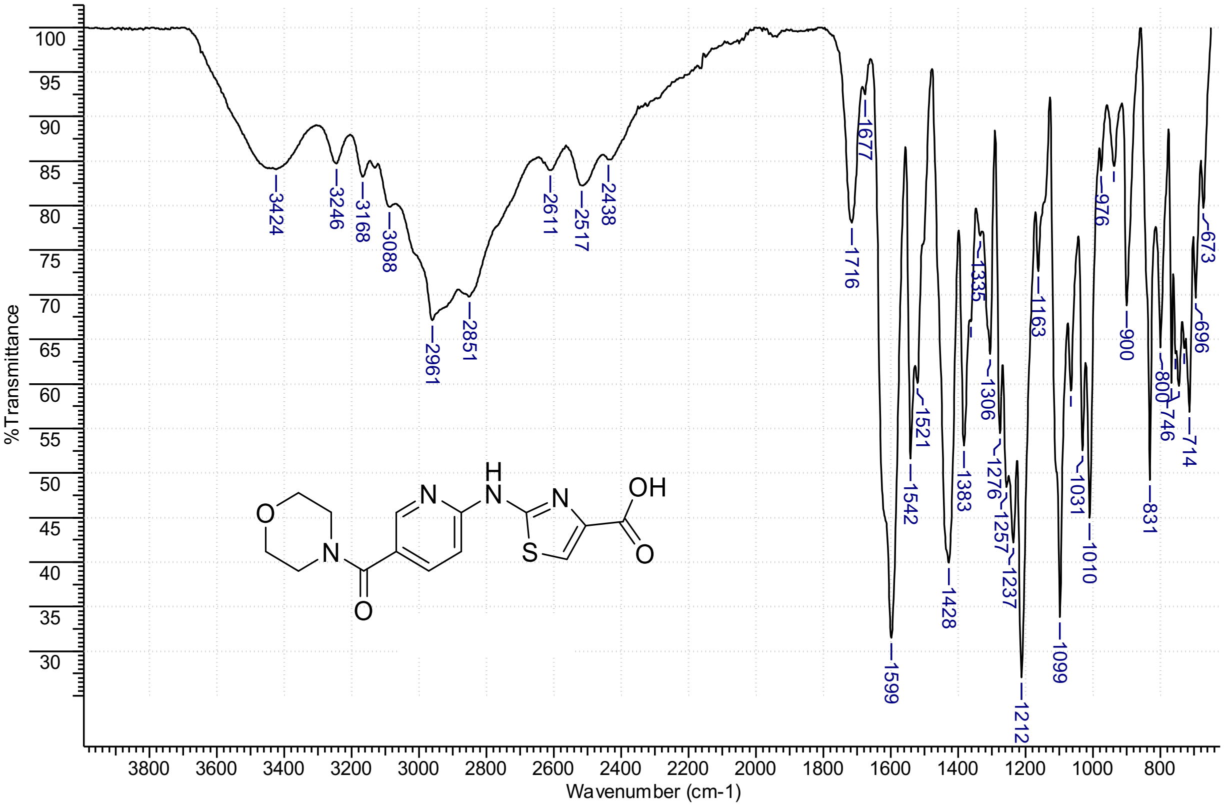

Figure S72. FT-IR spectrum of intermediate Cg

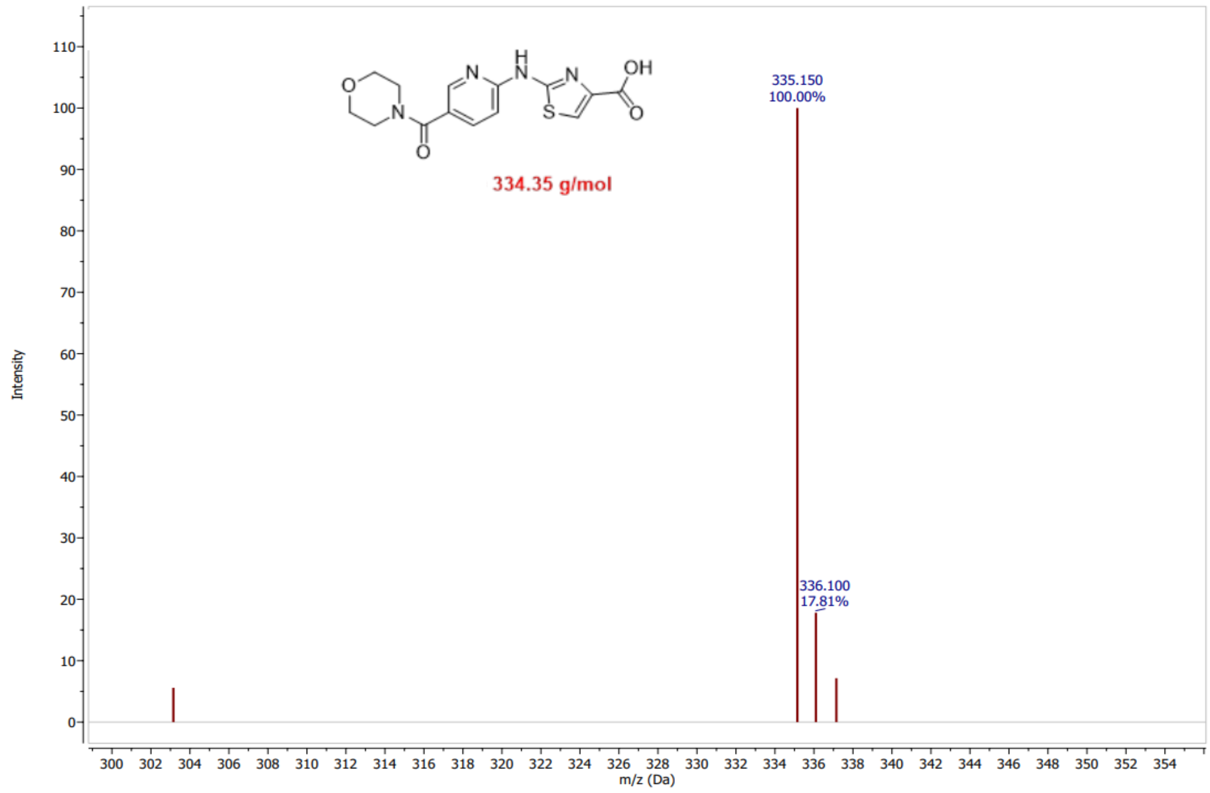

Figure S73. LC-MS/MS spectrum of intermediate Cg

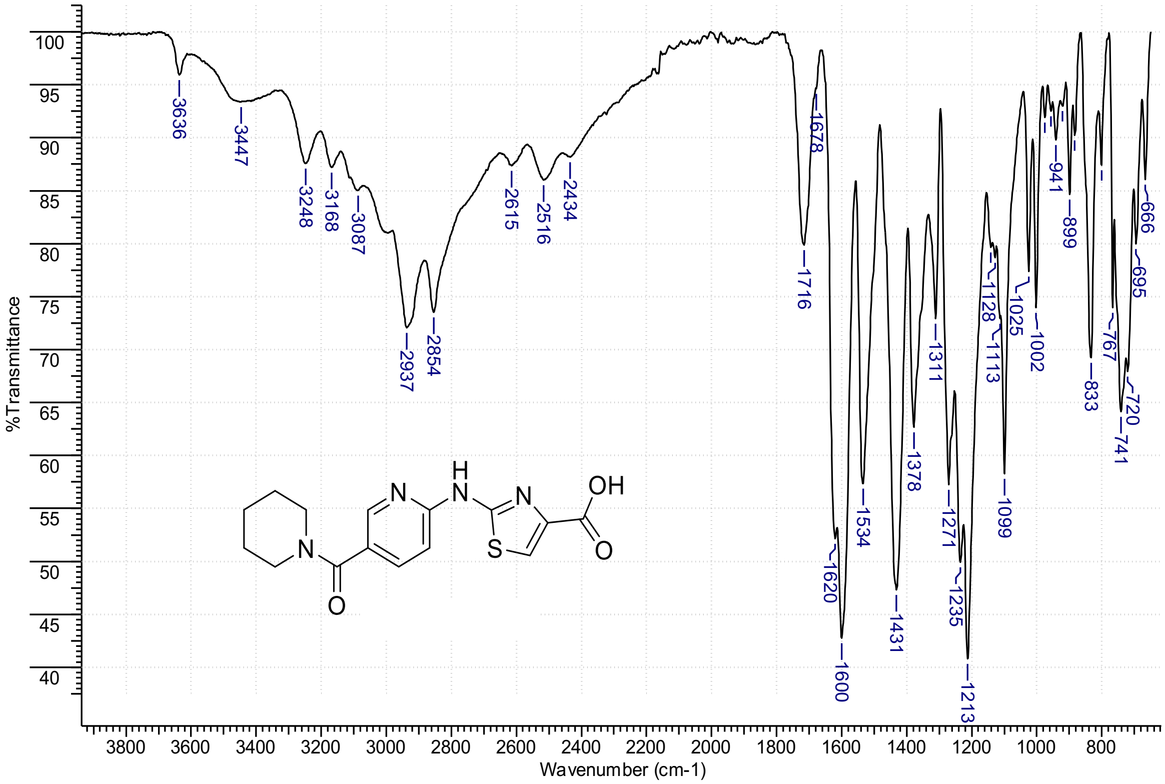

Figure S74. FT-IR spectrum of intermediate Ch

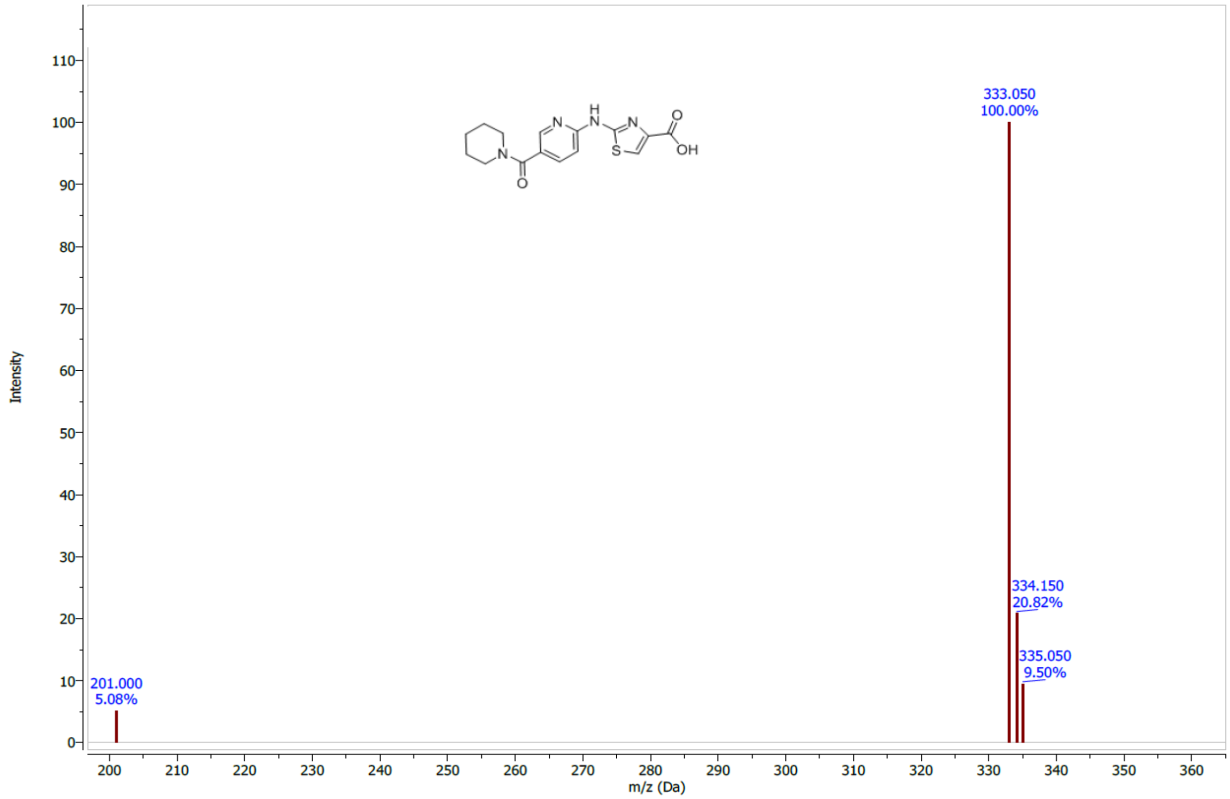

Figure S75. LC-MS/MS spectrum of intermediate Ch

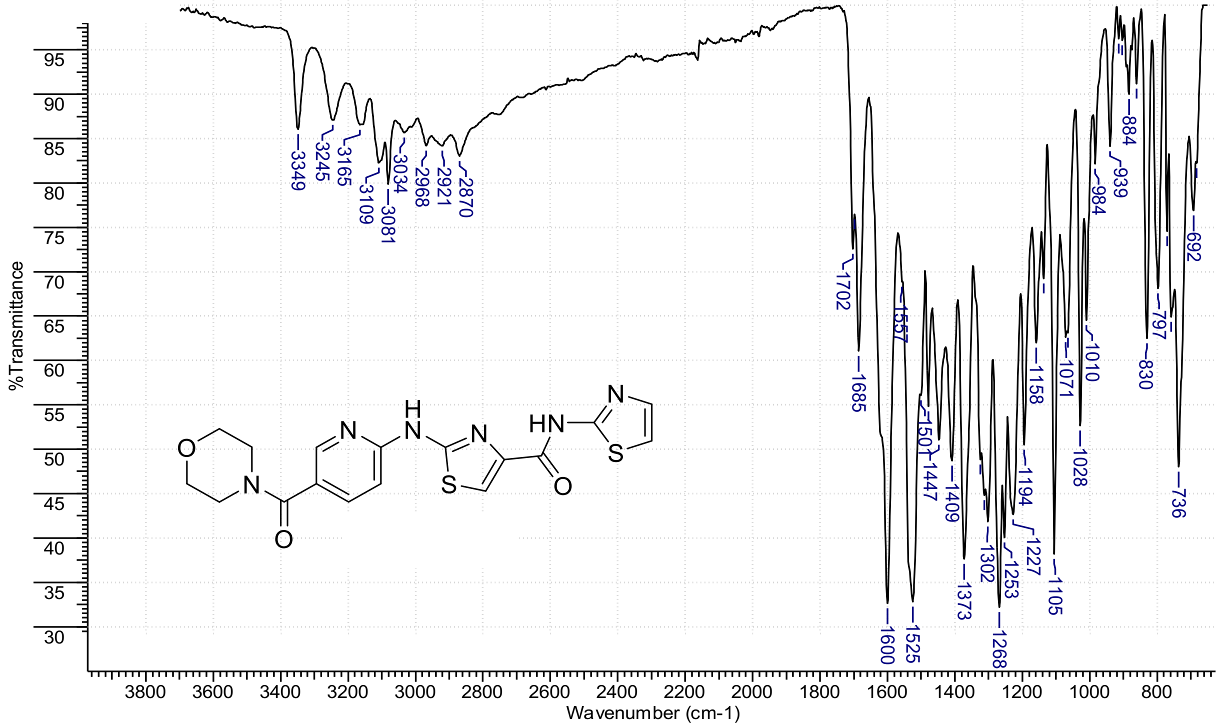

Figure S76. FT-IR spectrum of compound **C10**

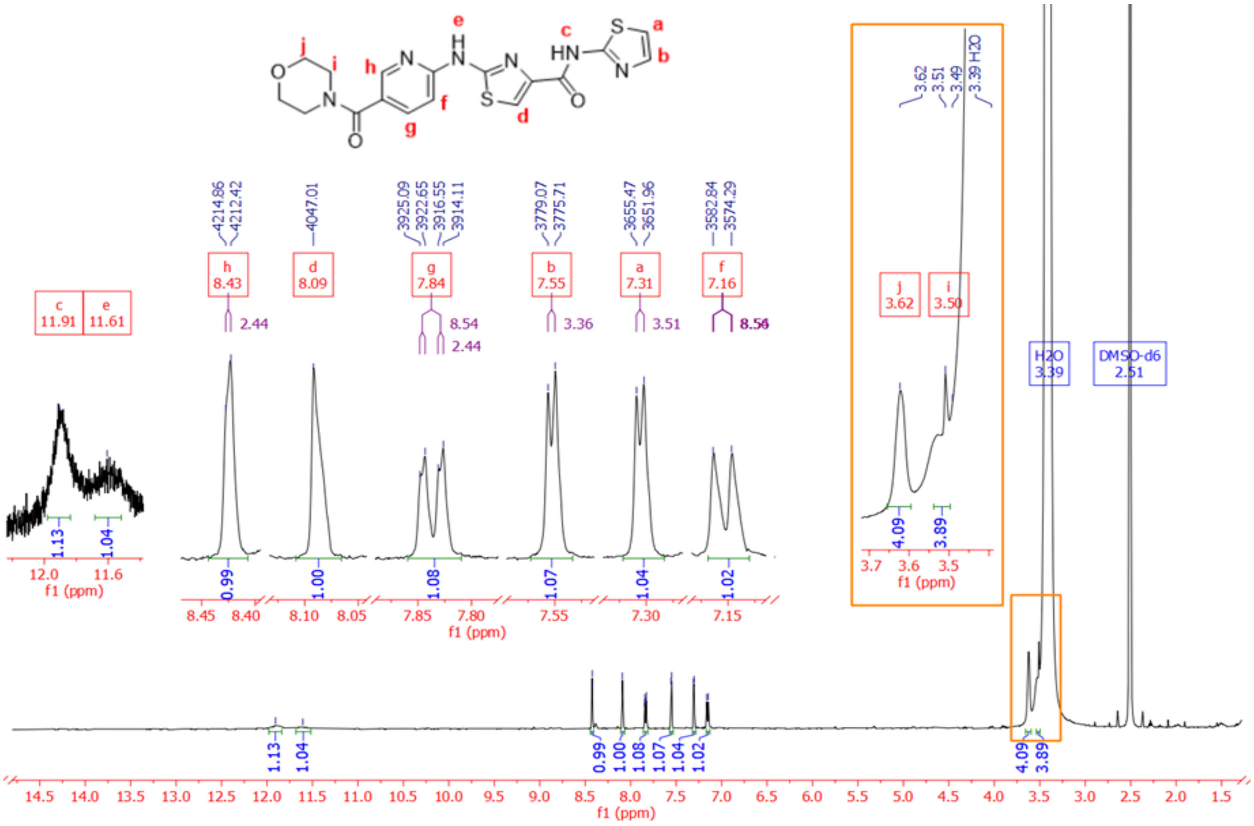

Figure S77. ^{1}H NMR spectrum of compound **C10** (500 MHz, DMSO-d_6)

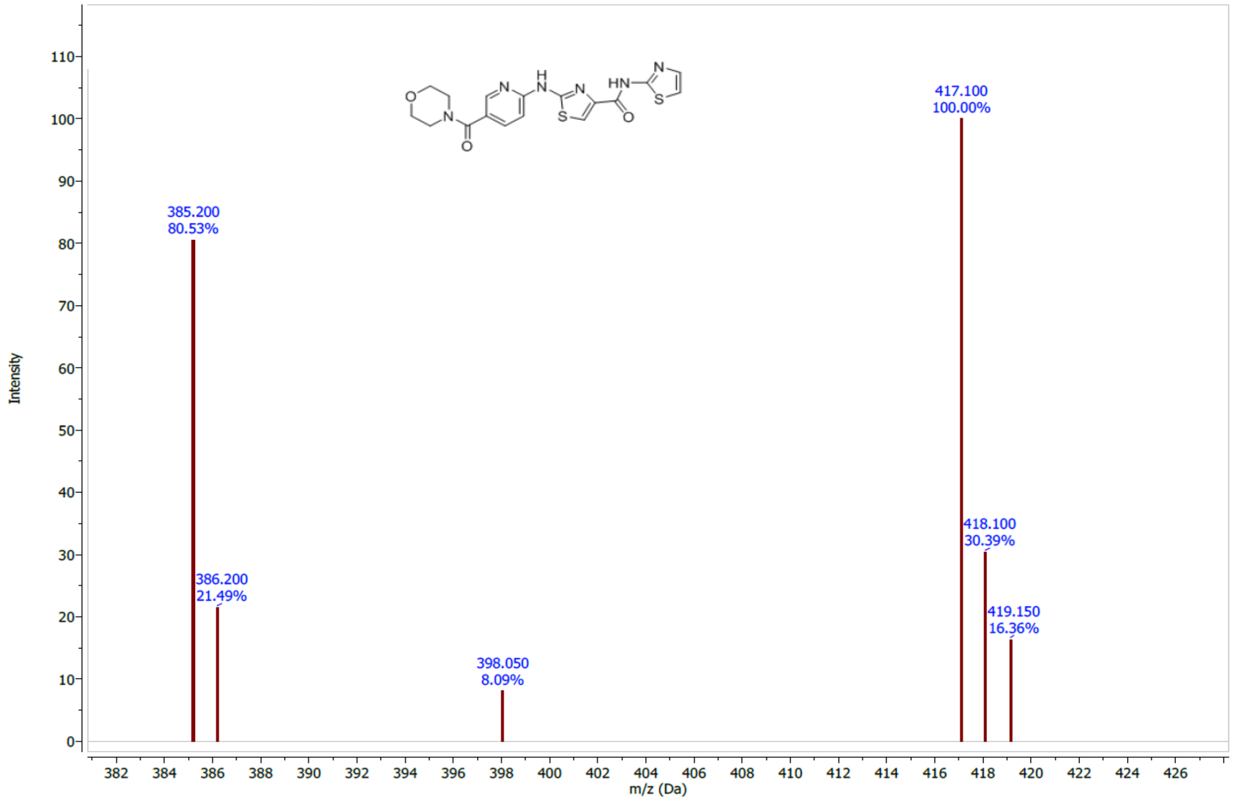

Figure S78. LC-MS/MS spectrum of compound **C10**

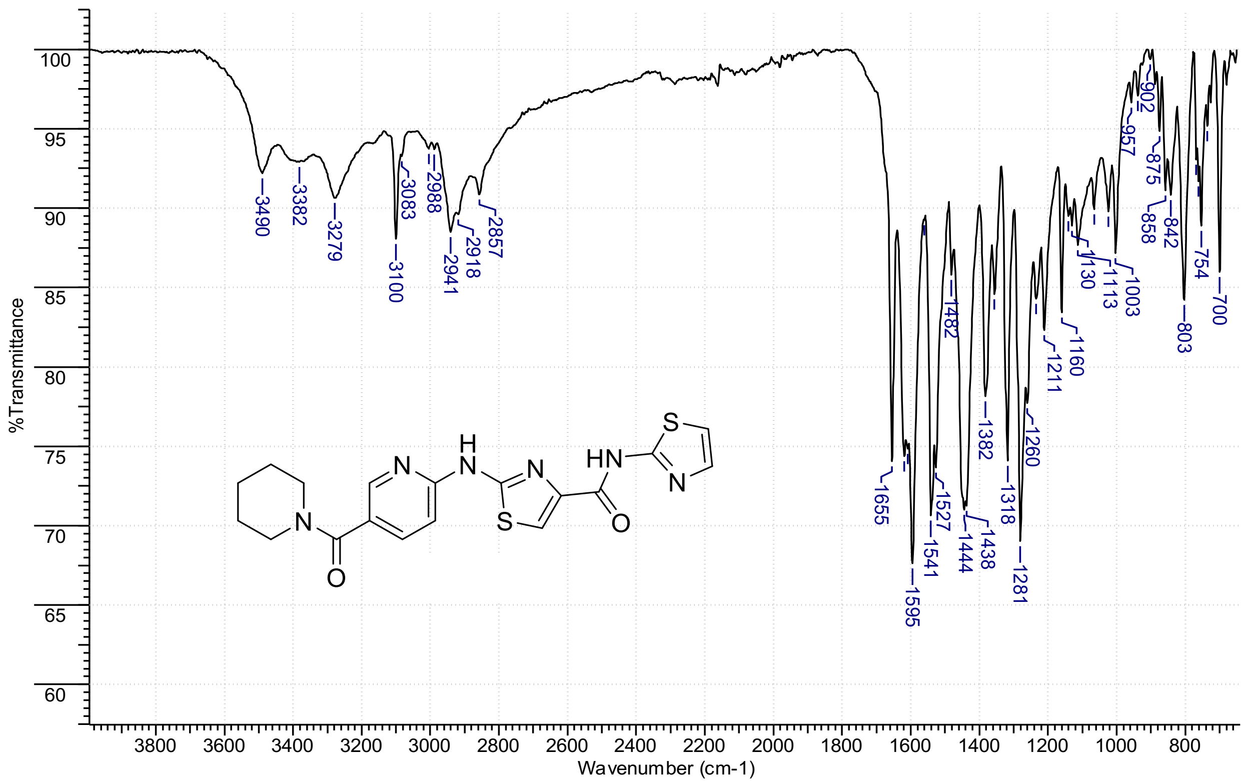

Figure S79. FT-IR spectrum of compound **C11**

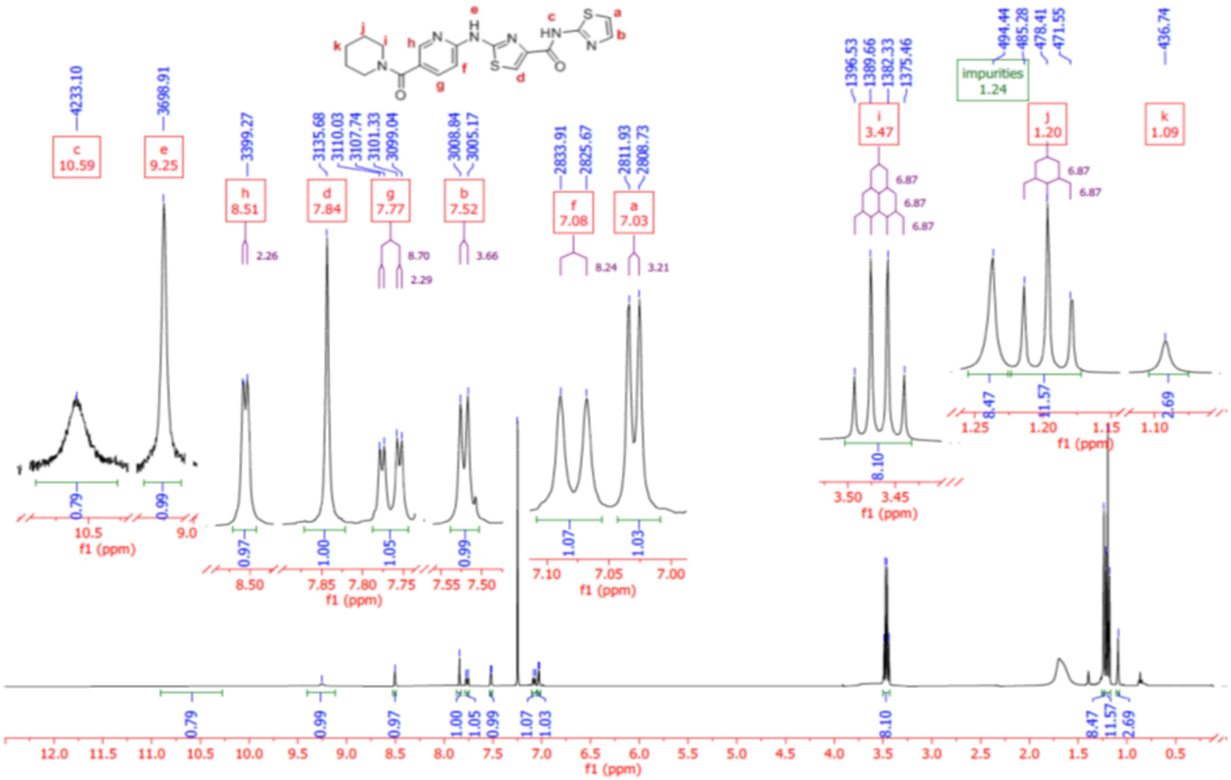

Figure S80. ^{1}H NMRspectrum of compound **C11** (400 MHz, $CDCl_3$)

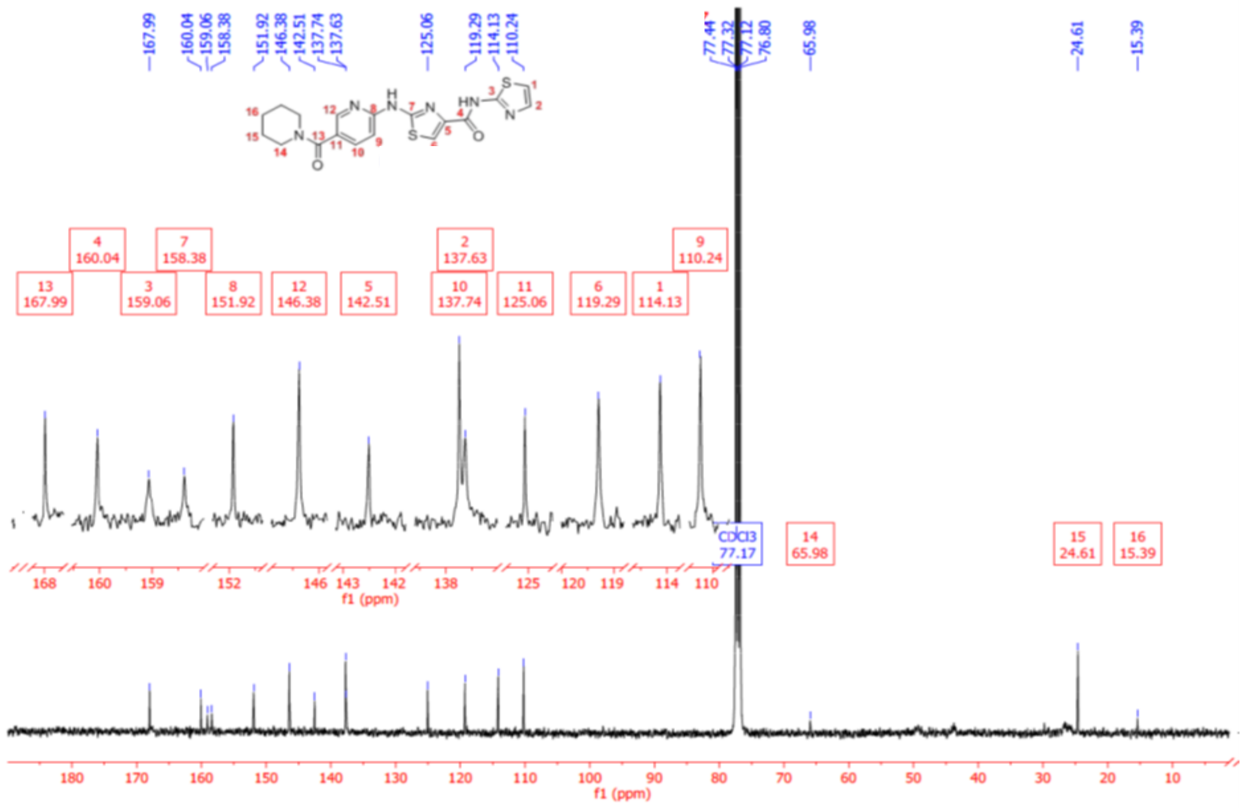

Figure S81. ^{13}C NMRspectrum of compound **C11** (100 MHz, $CDCl_3$)

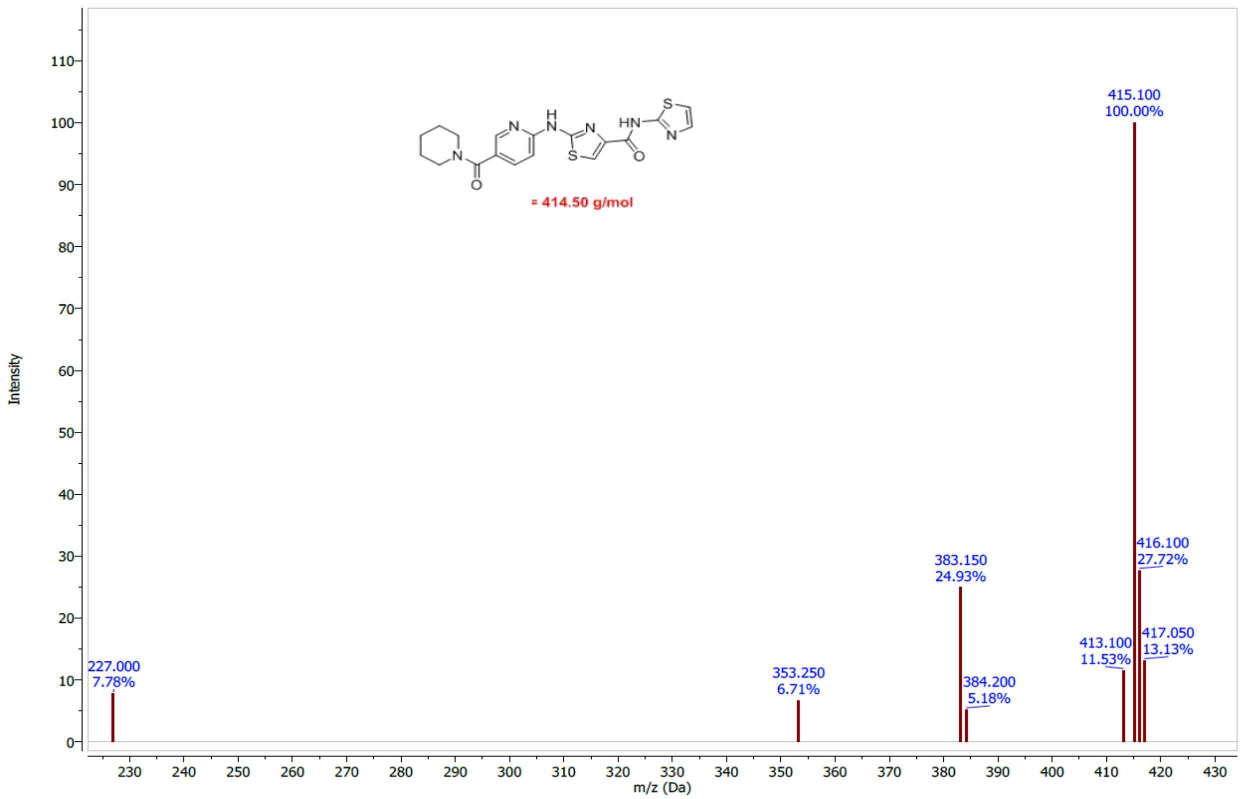

Figure S82. LC-MS/MS spectrum of compound **C11**

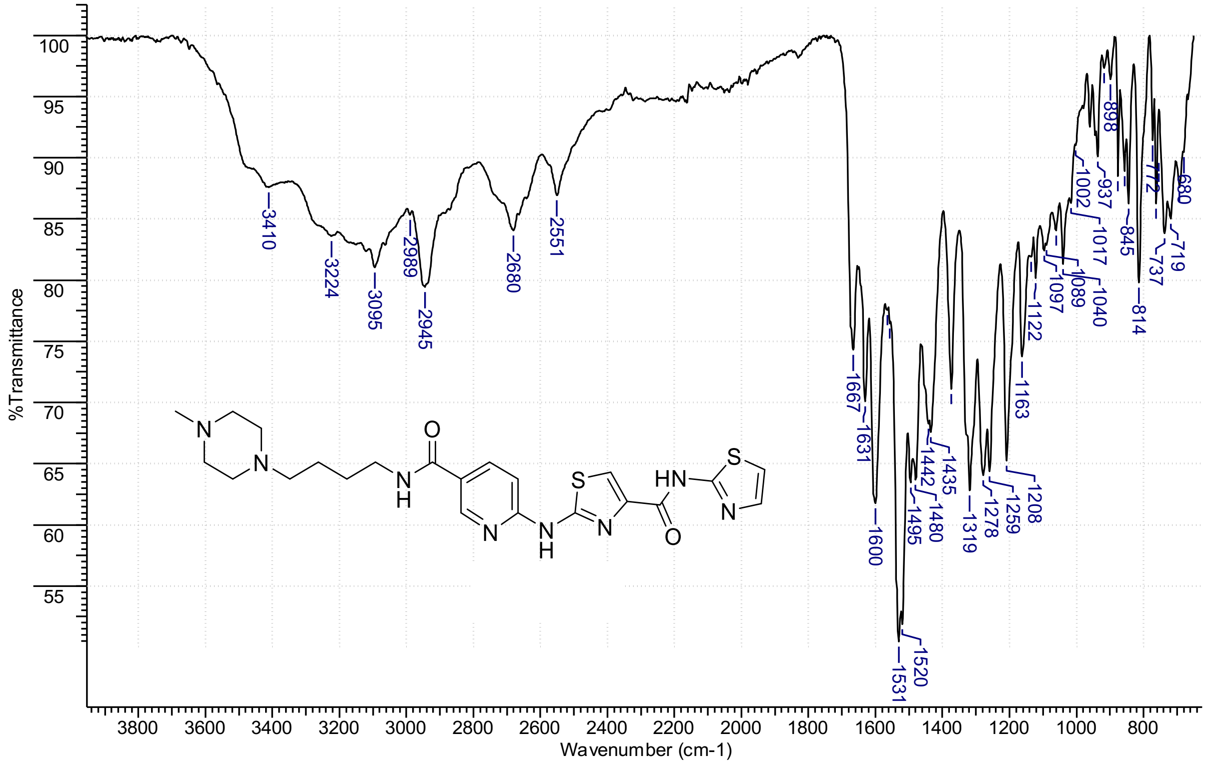

Figure S83. FT-IR spectrum of compound **C12**

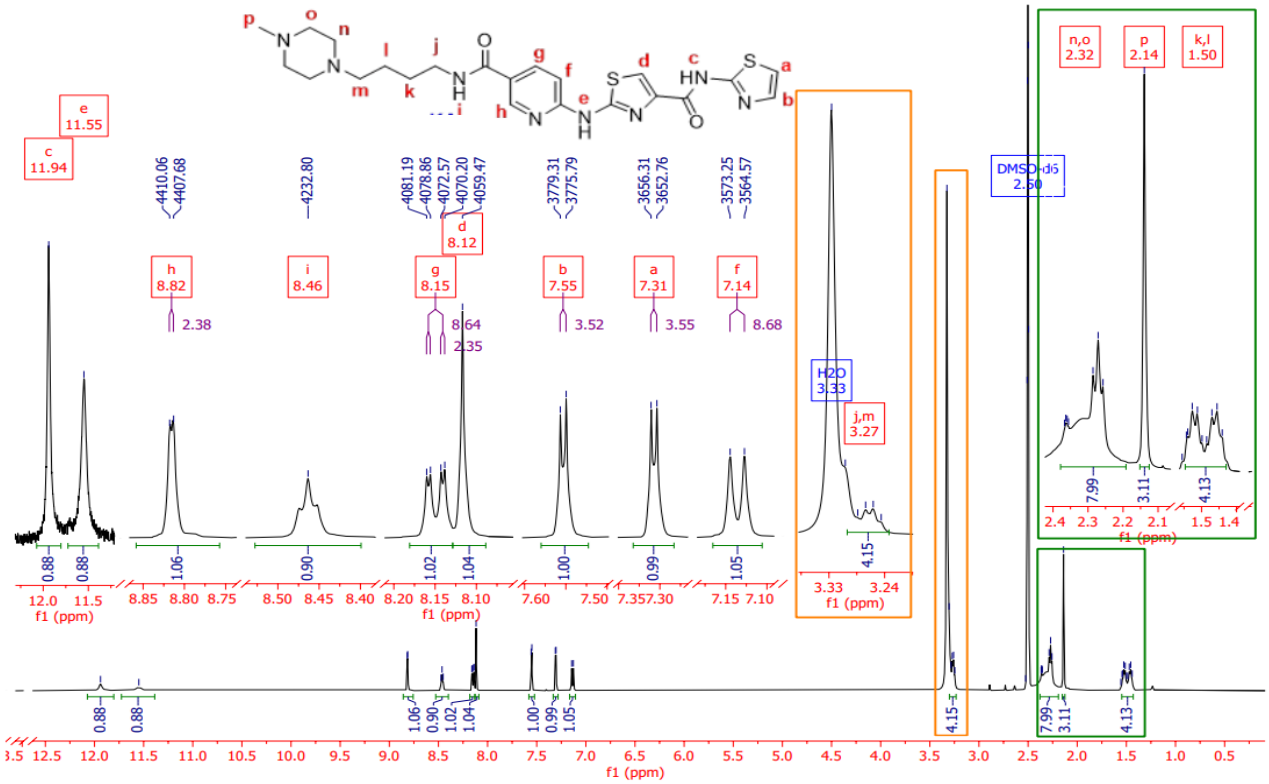

Figure S84. ^{1}H NMR spectrum of compound **C12** (500 MHz, DMSO-d6)

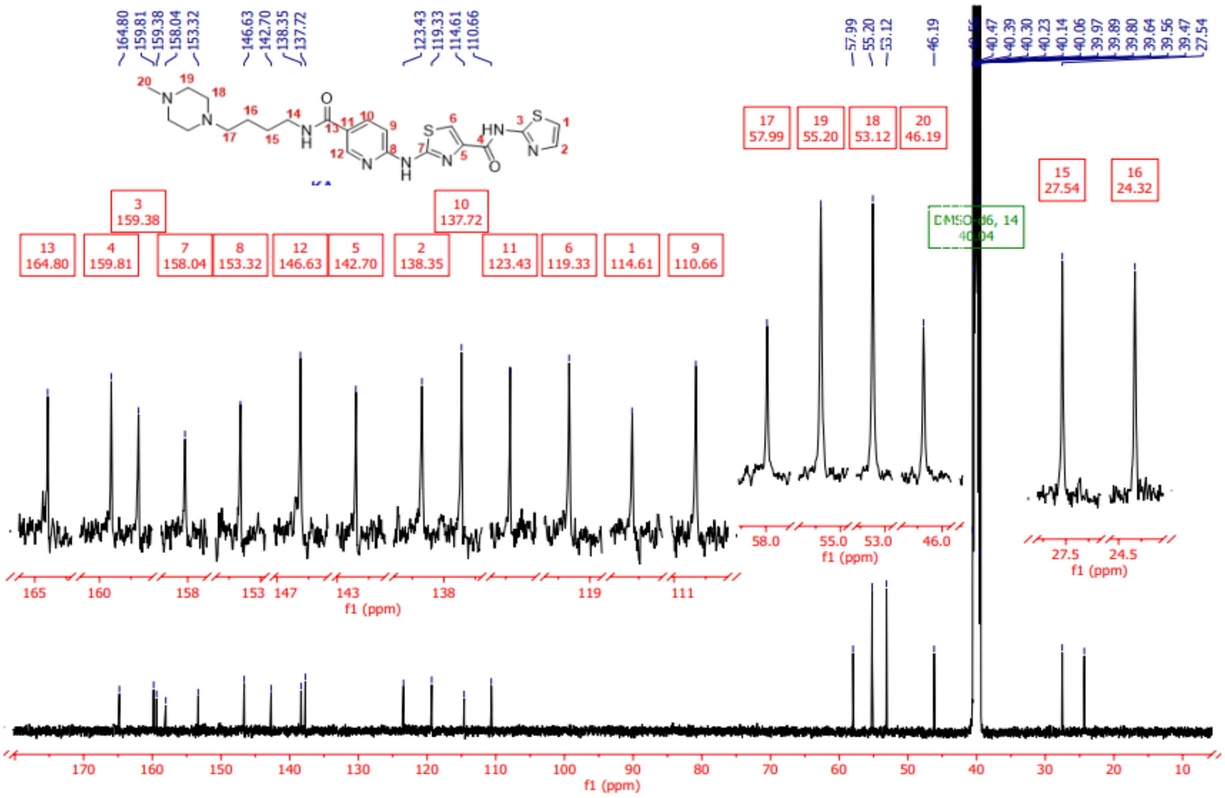

Figure S85. ^{13}C NMRspectrum of compound **C12** (125 MHz, DMSO-d_6)

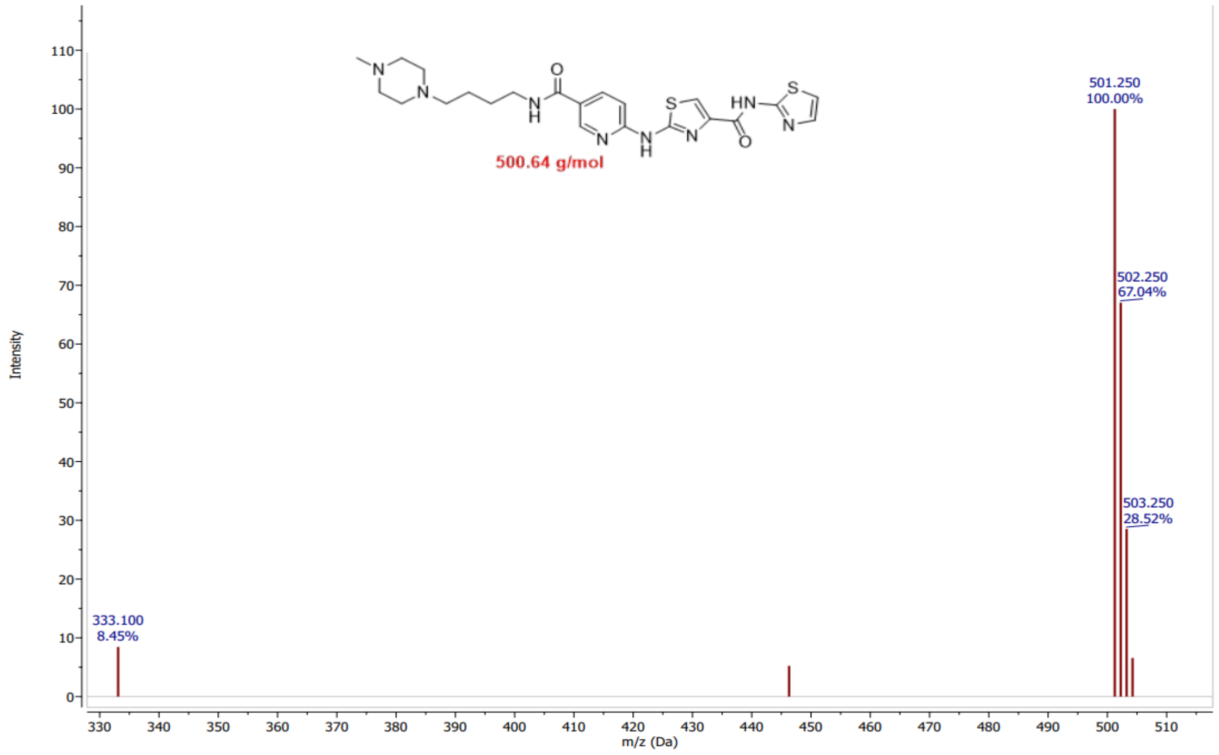

Figure S86. LC-MS/MS spectrum of compound **C12**

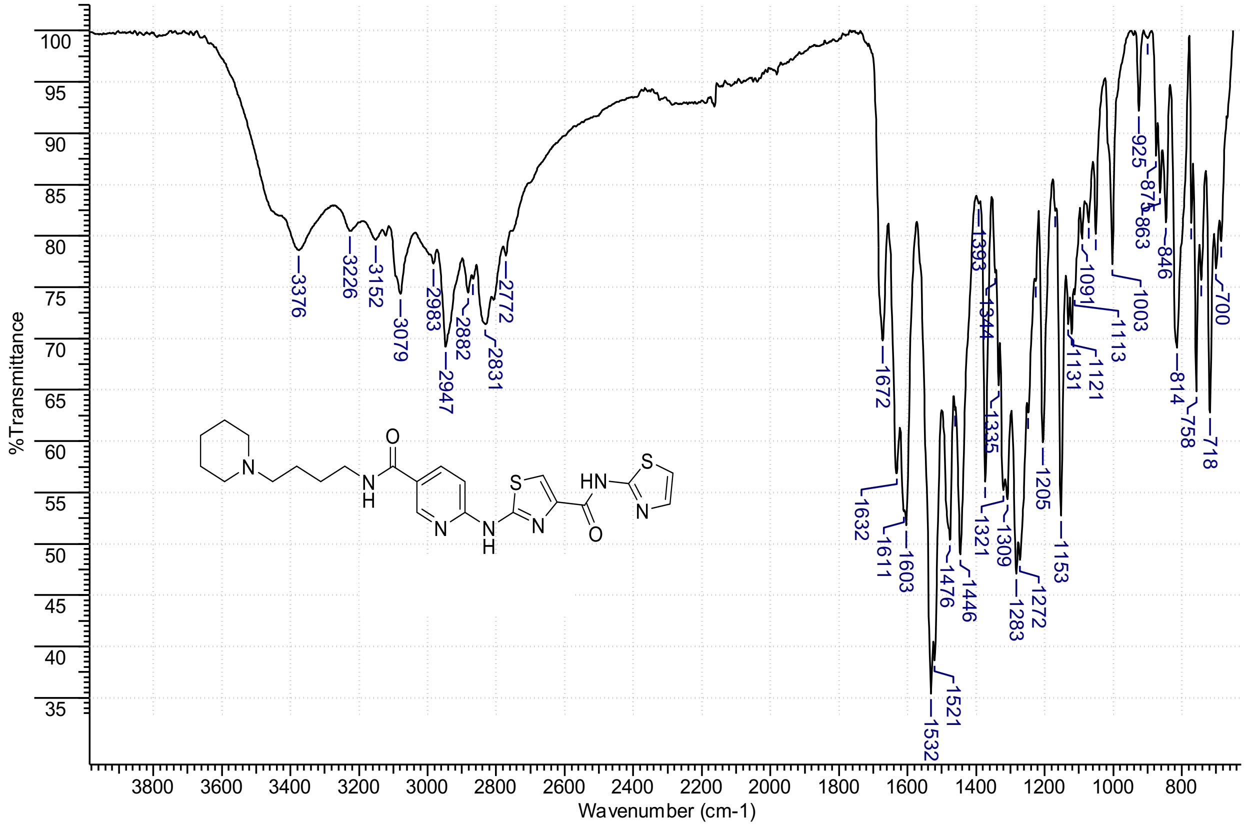

Figure S87. FT-IR spectrum of compound **C13**

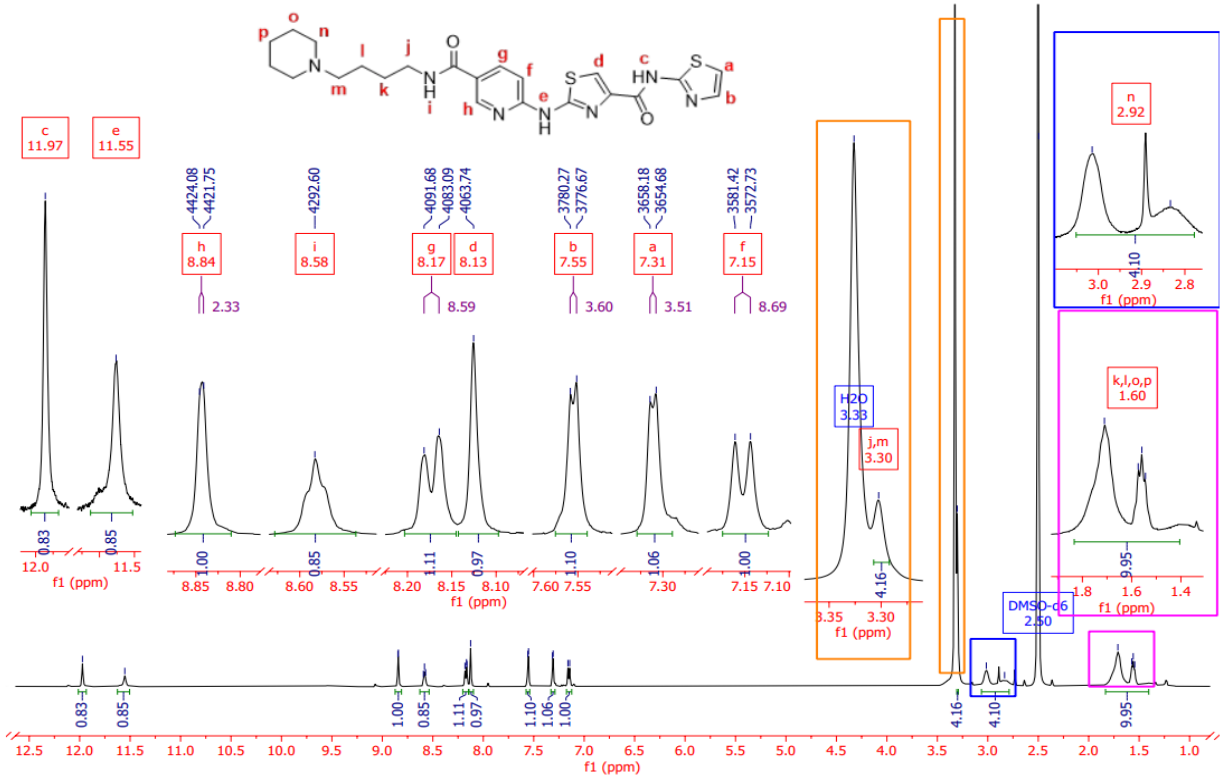

Figure S88. ^{1}H NMR spectrum of compound **C13** (500 MHz, DMSO-d_6)

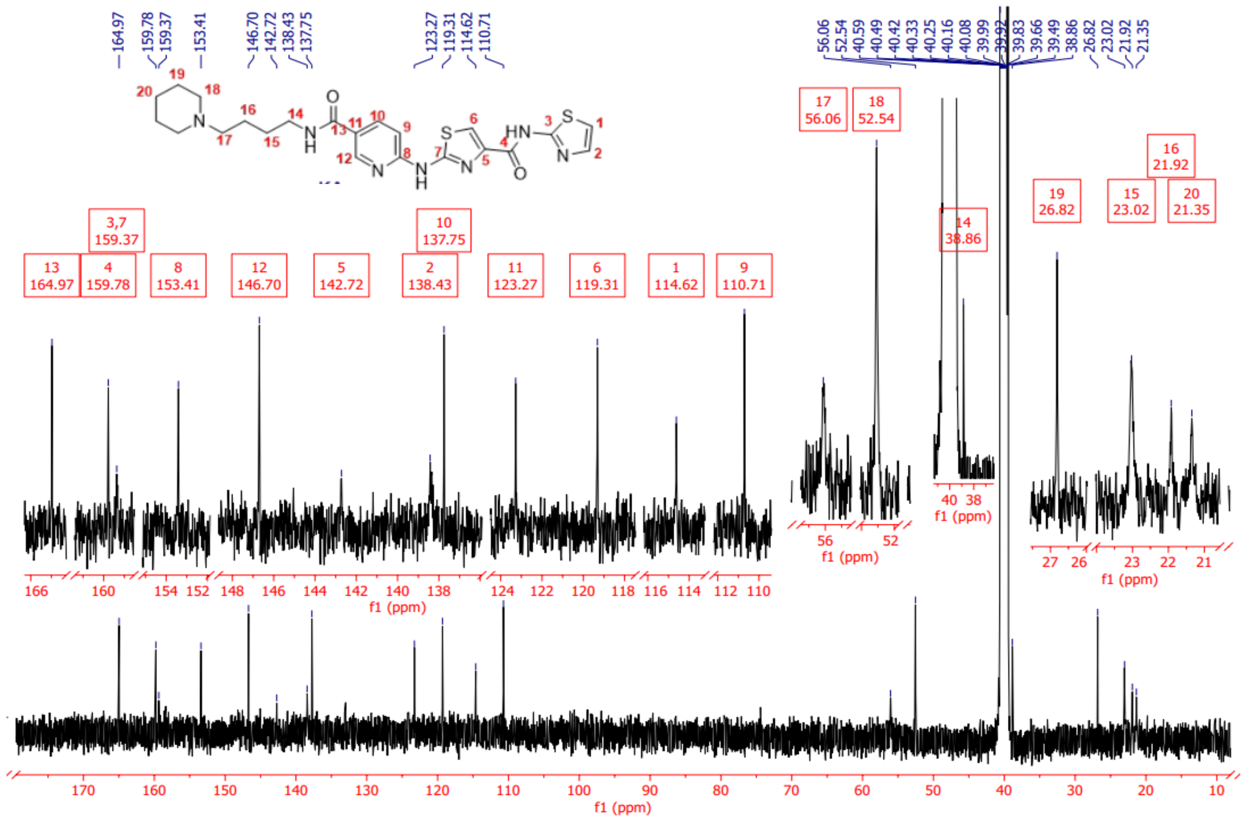

Figure S89. ^{13}C NMR spectrum of compound **C13** (125 MHz, DMSO-d_6)

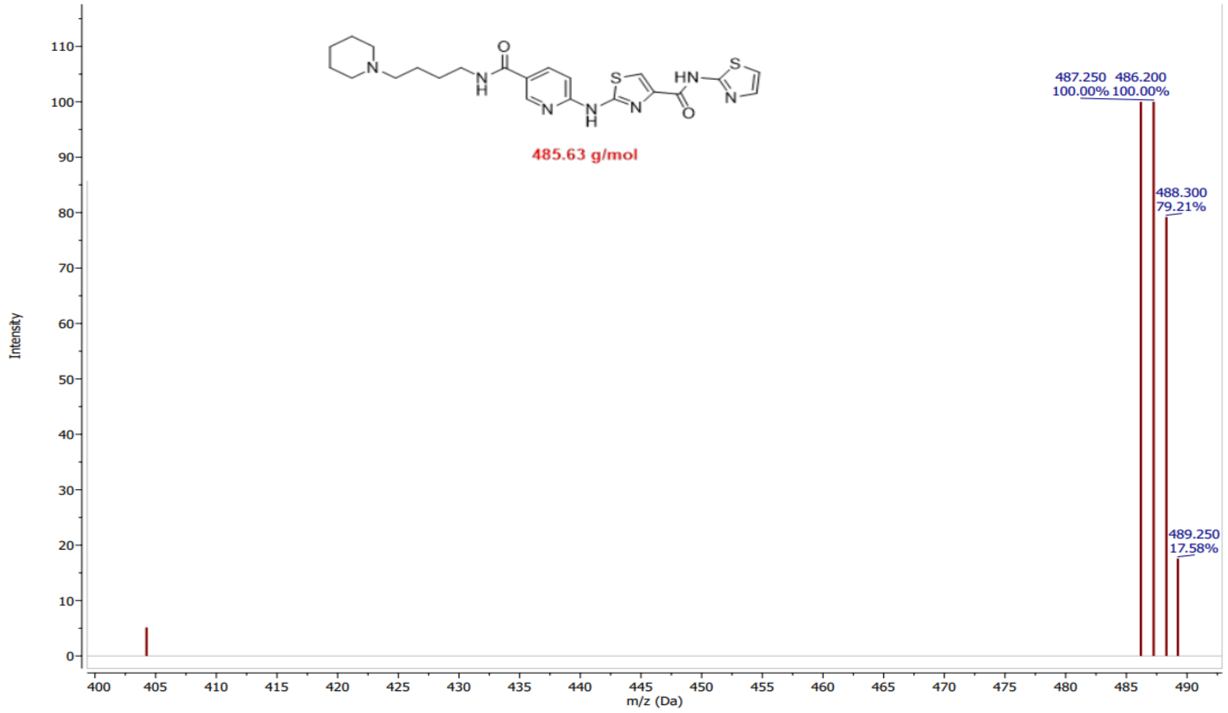

Figure S90. LC-MS/MS spectrum of compound **C13**

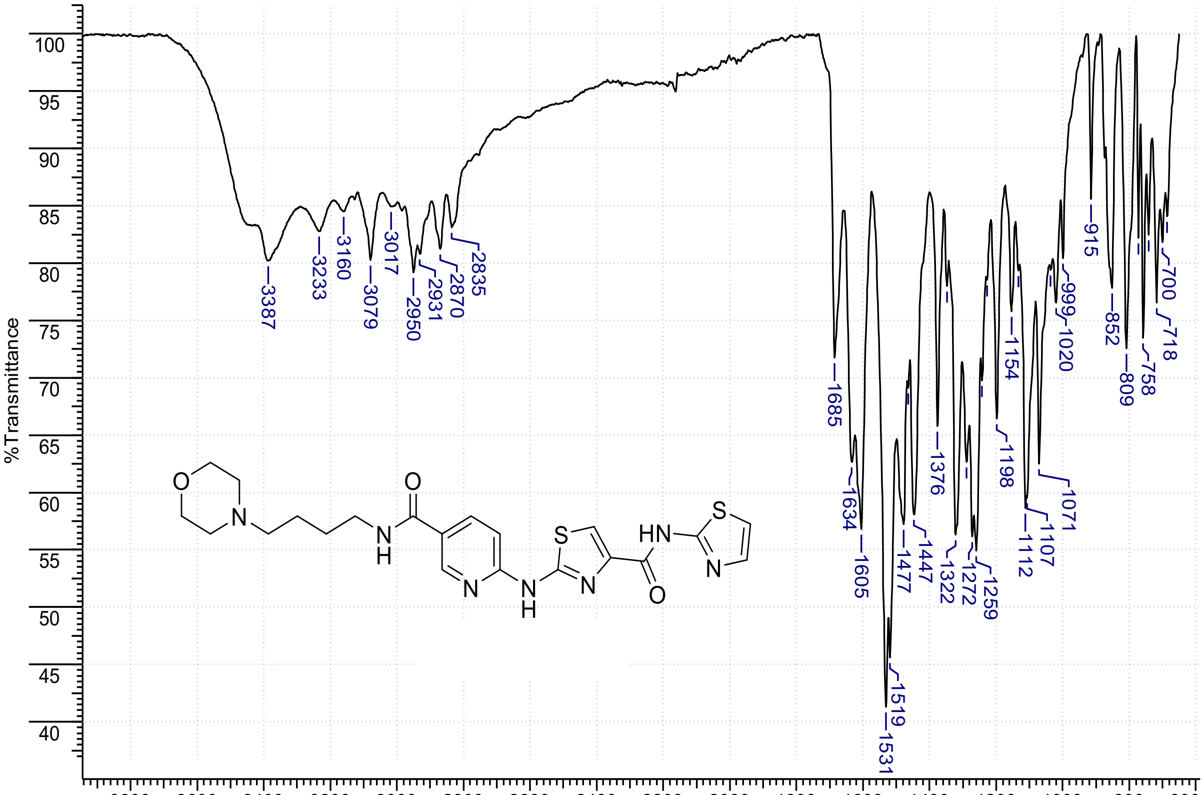

Figure S91. FT-IR spectrum of compound **C14**

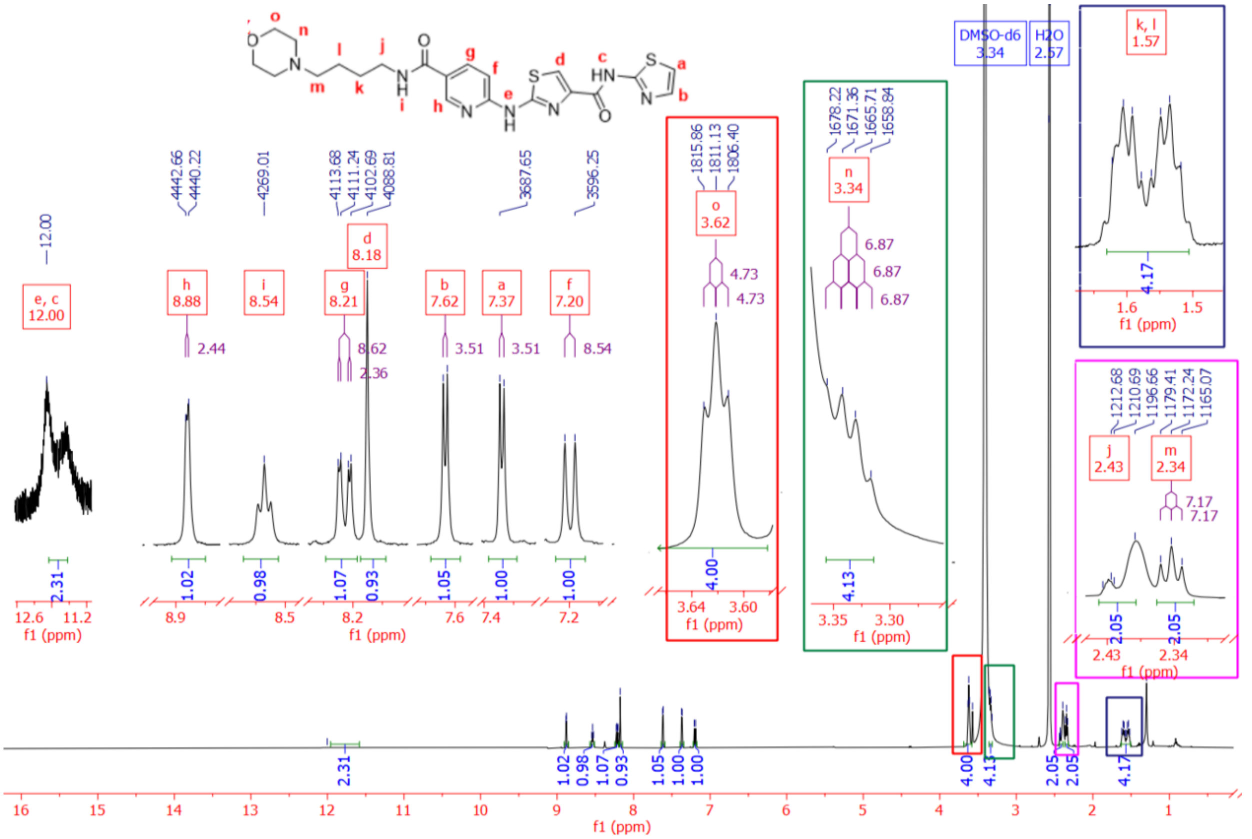

Figure S92. ^{1}H NMR spectrum of compound **C14** (500 MHz, DMSO-d_6)

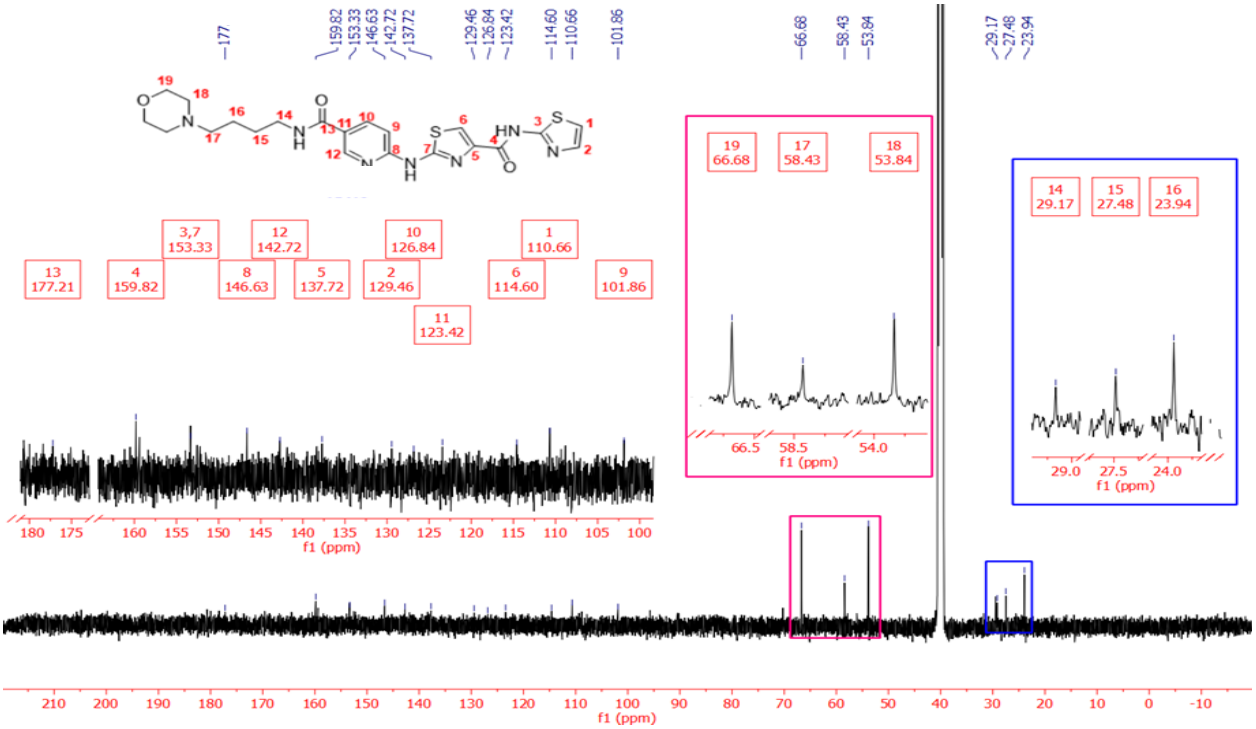

Figure S93. ^{13}C NMR spectrum of compound **C14** (125 MHz, DMSO-d_6)

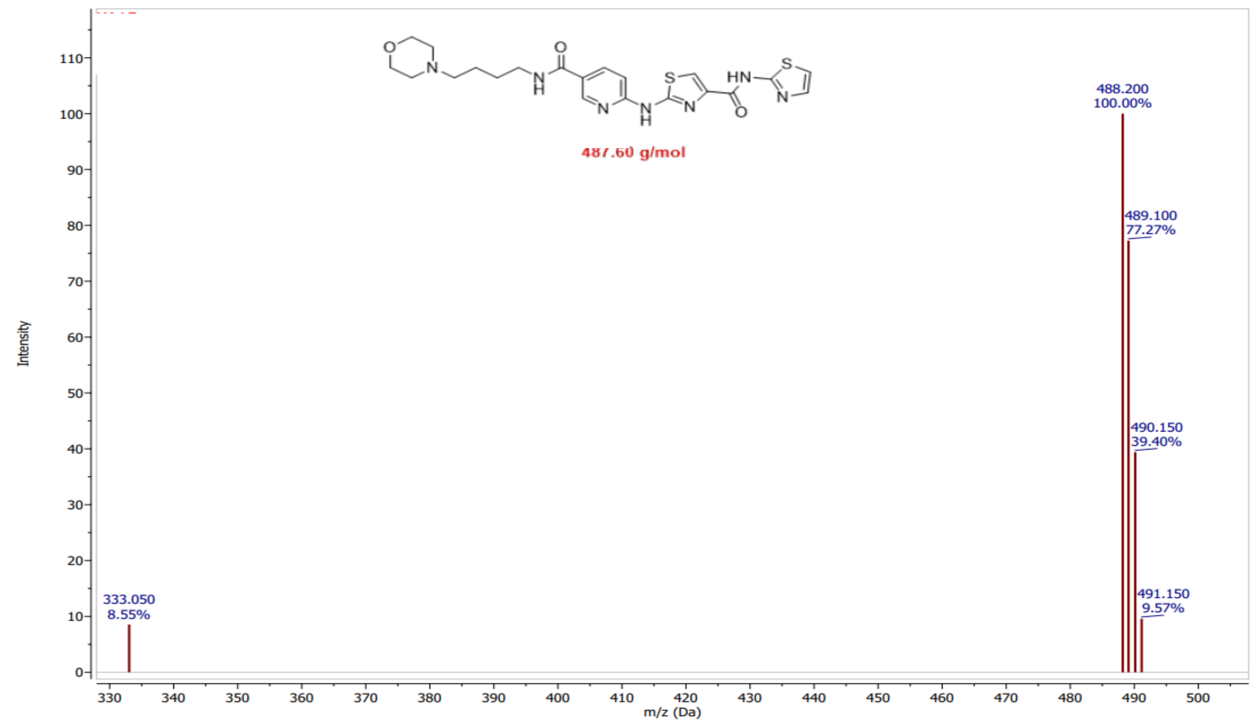

Figure S94. LC-MS/MS spectrum of compound **C14**

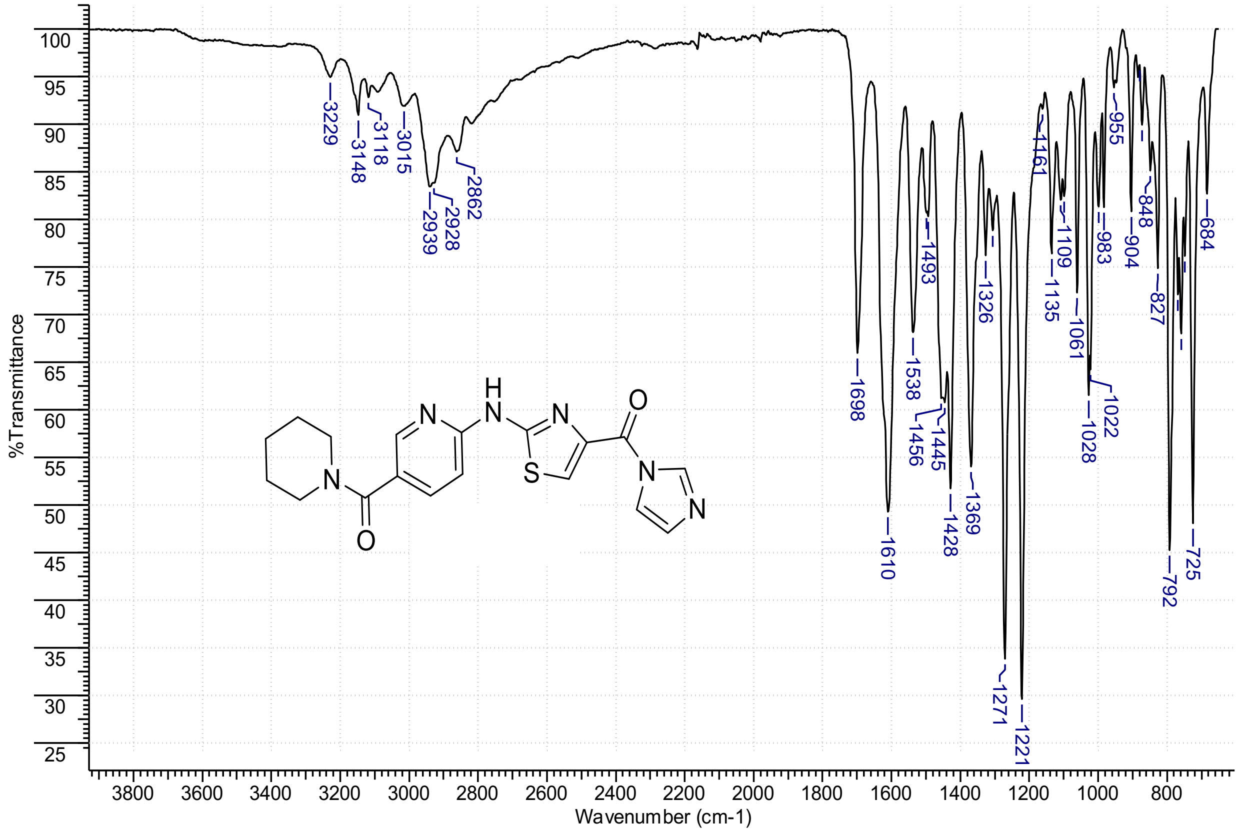

Figure S95. FT-IR spectrum of compound **C15**

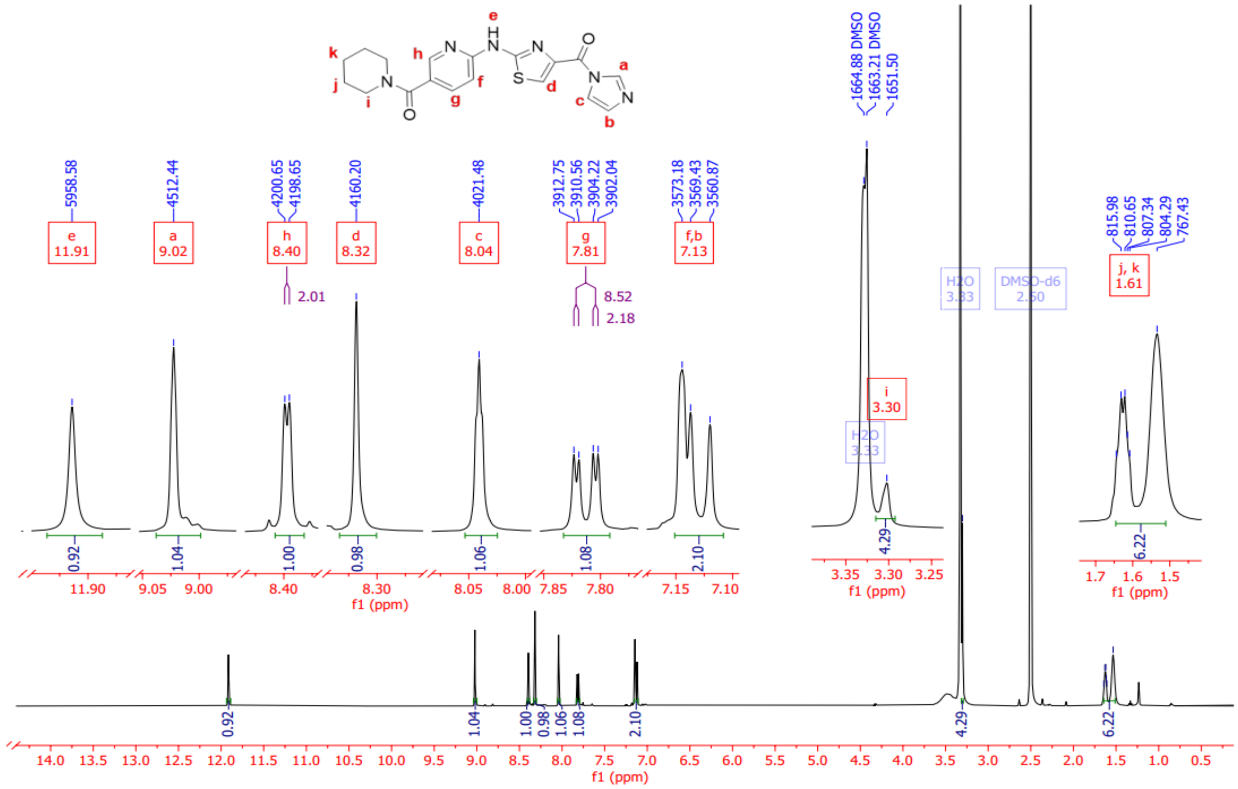

Figure S96. ^{1}H NMR spectrum of compound **C15** (500 MHz, DMSO-d$_6$)

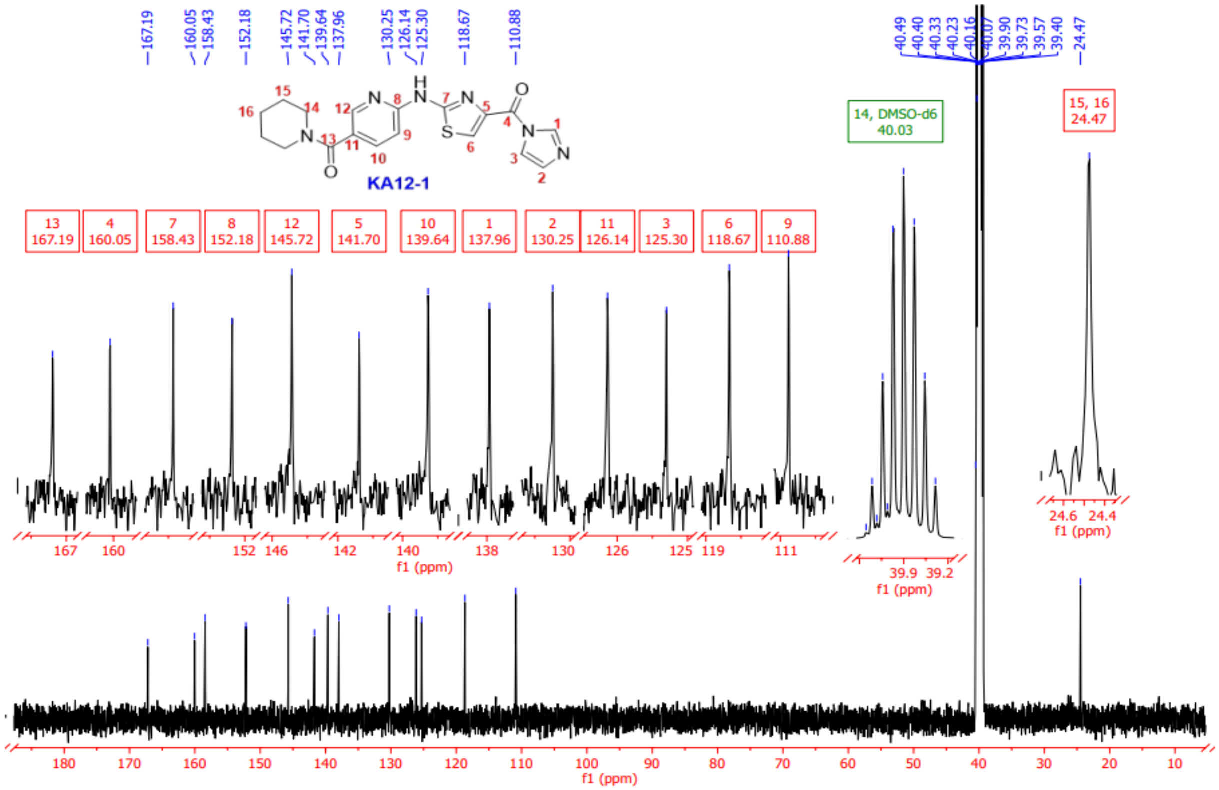

Figure S97. ^{13}C NMR spectrum of compound **C15** (125 MHz, DMSO-d$_6$)

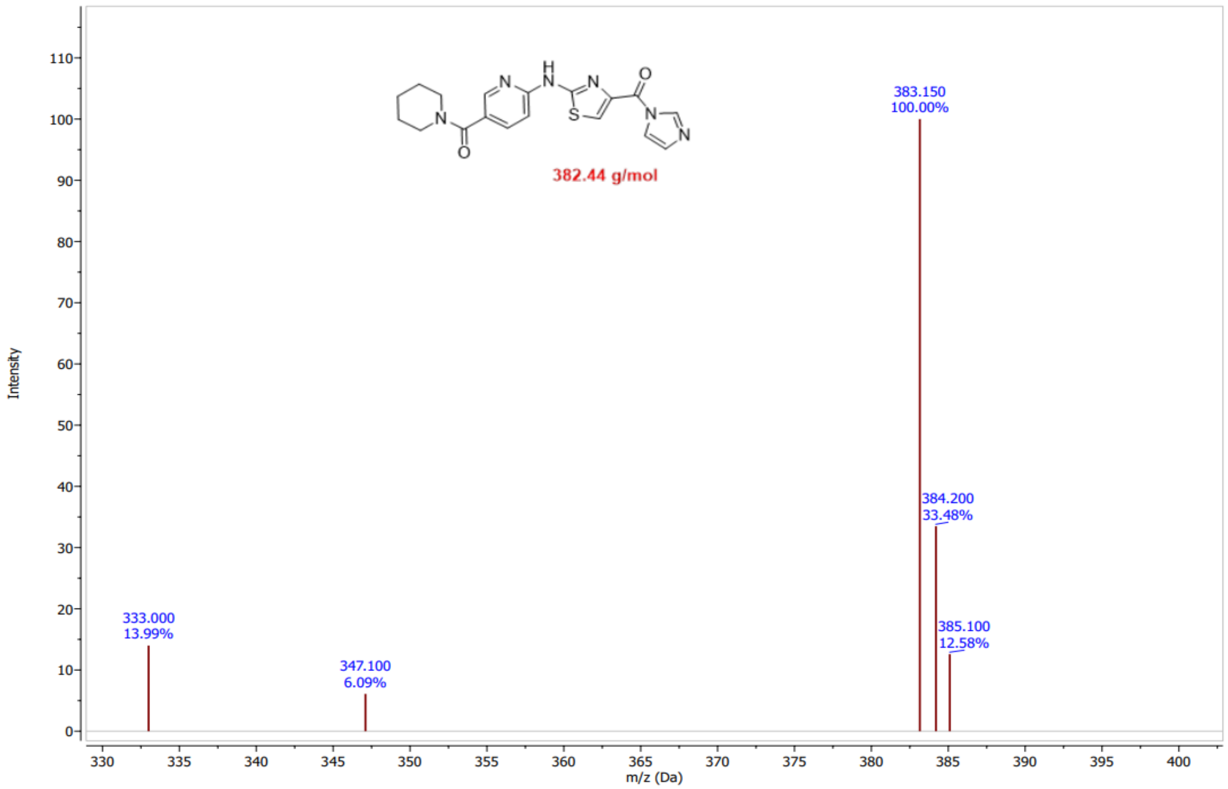

Figure S98. LC-MS/MS spectrum of compound **C15**
